# Zinc finger structure determination by NMR: Why zinc fingers can be a handful

**DOI:** 10.1016/j.pnmrs.2022.07.001

**Published:** 2022-07-15

**Authors:** David Neuhaus

**Affiliations:** MRC Laboratory of Molecular Biology, Francis Crick Avenue, Cambridge CB2 0QH, UK

**Keywords:** Zinc finger, NMR spectroscopy, NMR structure determination, Protein structure, Metallothionein

## Abstract

Zinc fingers can be loosely defined as protein domains containing one or more tetrahedrally-co-ordinated zinc ions whose role is to stabilise the structure rather than to be involved in enzymatic chemistry; such zinc ions are often referred to as “structural zincs”. Although structural zincs can occur in proteins of any size, they assume particular significance for very small protein domains, where they are often essential for maintaining a folded state. Such small structures, that sometimes have only marginal stability, can present particular difficulties in terms of sample preparation, handling and structure determination, and early on they gained a reputation for being resistant to crystallisation. As a result, NMR has played a more prominent role in structural studies of zinc finger proteins than it has for many other types of proteins. This review will present an overview of the particular issues that arise for structure determination of zinc fingers by NMR, and ways in which these may be addressed.

## Graphical abstract

**Figure:**
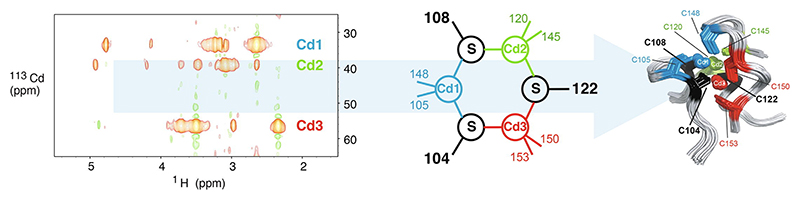

## 1 Introduction

**Figure 1:**
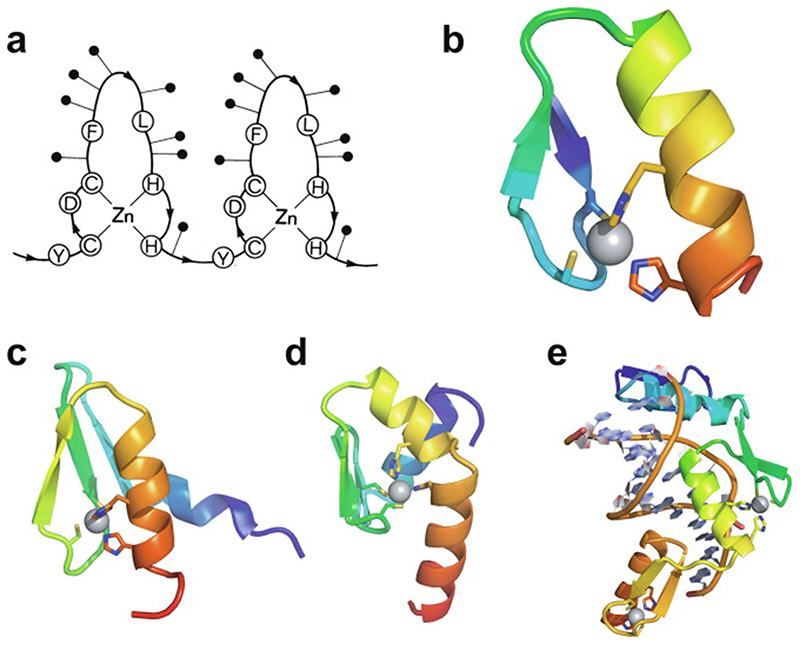
TFIIIA-type (C_2_H_2_) zinc finger structures. **a**) Original schematic proposal of how zinc fingers are organised in the *Xenopus laevis* transcription factor TFIIIA, reproduced with permission from the original paper of Miller *et al.* [1] (© 1985 European Molecular Biology Organization). **b**) NMR structure of zinc finger 31 from the protein Xfin (also from *Xenopus*), which was the first experimentally determined structure of a zinc finger (PDB 1znf)[3]. **c**) NMR structure of zinc finger 1 from the yeast transcription factor SWI5(PDB1ncs)[9];this structure includes an additional *N*-terminal β-strand and α-helix relative to the canonical TFIIIA-type finger. **d**)NMR structure of zinc finger 1 from the *Xenopus* protein Zfa (PDB 1zu1), which in addition to an N-terminal three-stranded β-sheet and α-helix also includes an additional C-terminal helix containing the fourth zinc-binding ligand at its *N*-terminus [10]. **e**) X-ray crystal structure of three zinc fingers of the transcription factor Zif268 bound to their cognate DNA sequence (PDB 1zaa) [13]; this was the first structure to show an example of how zinc fingers recognise specific sequences in DNA (the interactions between protein sidechains and DNA responsible for this recognition are not shown in this figure). Smoothed protein backbone cartoons were produced using the program PyMol [23,24] and are shown in “chainbow” colouring (blue → red, N-terminal → C-terminal), zinc ions are shown as grey spheres and all the NMR structures are illustrated using only the lowest energy member of the deposited ensemble in each case.

In 1985, Miller, McLachlan and Klug discovered that TFIIIA, a transcription factor from Xenopus laevis that they were studying, contained within its sequence a set of nine small, independently folded, zinc-binding mini-domains of about 30 amino acids each, which they proposed acted together to mediate sequence-specific binding of the protein to DNA [1]. In this seminal paper, they proposed that each of these motifs is held together by two cysteines and two histidines in conserved sequence locations, which collectively bind a single zinc ion with tetrahedral geometry (Fig. 1a). A sequence of only 30 amino acids would not generally be expected to be capable of folding independently based just on the non-covalent interactions (hydrogen-bonding, electrostatic and van der Waals interactions, etc.) normally associated with stabilisation of the folded states of proteins, implying that the bound zinc plays a key role in strengthening the structure. The authors suggested that each motif, or “finger” as they termed them (the name “zinc finger” seems to have first been used explicitly slightly later by Berg [2]), recognised and grasped a different short sequence in the DNA; although the selectivity provided by a single finger might be limited, the co-operative effect of several fingers binding simultaneously provides much greater specificity.

The first experimentally determined structure of a zinc finger was published in 1989 by the Wright group [3] (a slightly earlier publication by Klevit turned out to be only a partial structure [4]), whose NMR solution structure of the 31st finger domain from the protein Xfin showed that the two cysteines were located near a turn in a two-stranded β-sheet while the two histidines were on successive turns of an α-helix (Fig. 1b) (a rather similar structural model had been predicted by Berg in the previous year [2]). The cysteines each bound zinc through their Nε atoms, while the histidines each bound zinc through their Nγ atoms. Subsequent NMR studies in the following few years showed very similar structures for a number of other TFIIIA-type zinc fingers from other DNA-binding proteins in their unbound forms [5–8], some of which contained additional structural elements such as *N*-terminal β-strands and/or helices (Fig. 1c and 1d) [6,9,10]. It was also demonstrated that adjacent fingers are usually [11], but not invariably [7], flexibly linked and largely structurally independent from one another in the unbound state, though slightly later studies used ^15^N relaxation data to show that on very short time scales (<10 ns) the motions of adjacent fingers can be highly correlated [12] (see also Section 4.5). Small variations started to turn up amongst the different structures; the sequence separations between the zinc-binding residues sometimes differed slightly (indeed, this was already seen amongst the nine zinc fingers present in TFIIIA), sometimes histidine residues were exchanged for cysteine, and sometimes histidine was found to use its Nδ atom rather than Nε for zinc binding. The first detailed view of TFIIIA-type zinc fingers bound to DNA was achieved in 1991 using X-ray crystallography, when Pavletich and Pabo determined a structure for three adjacent fingers of the transcription factor Zif268 bound to its cognate DNA sequence (Fig. 1e) [13]; further crystal structures of such fingers bound to DNA soon followed [14–16], and two solution NMR structures of TFIIIA-type zinc fingers in complex with their DNA binding sites followed a few years later [17,18]. From these early beginnings a whole field grew up, establishing an “interaction code” through which these proteins achieved sequence-specific DNA recognition [19,20], and eventually extending to artificially manipulating TFIIIA-type zinc fingers effectively to achieve an early form of gene editing (well prior to the development of CRISPER/Cas9) [21,22].

**Figure 2:**
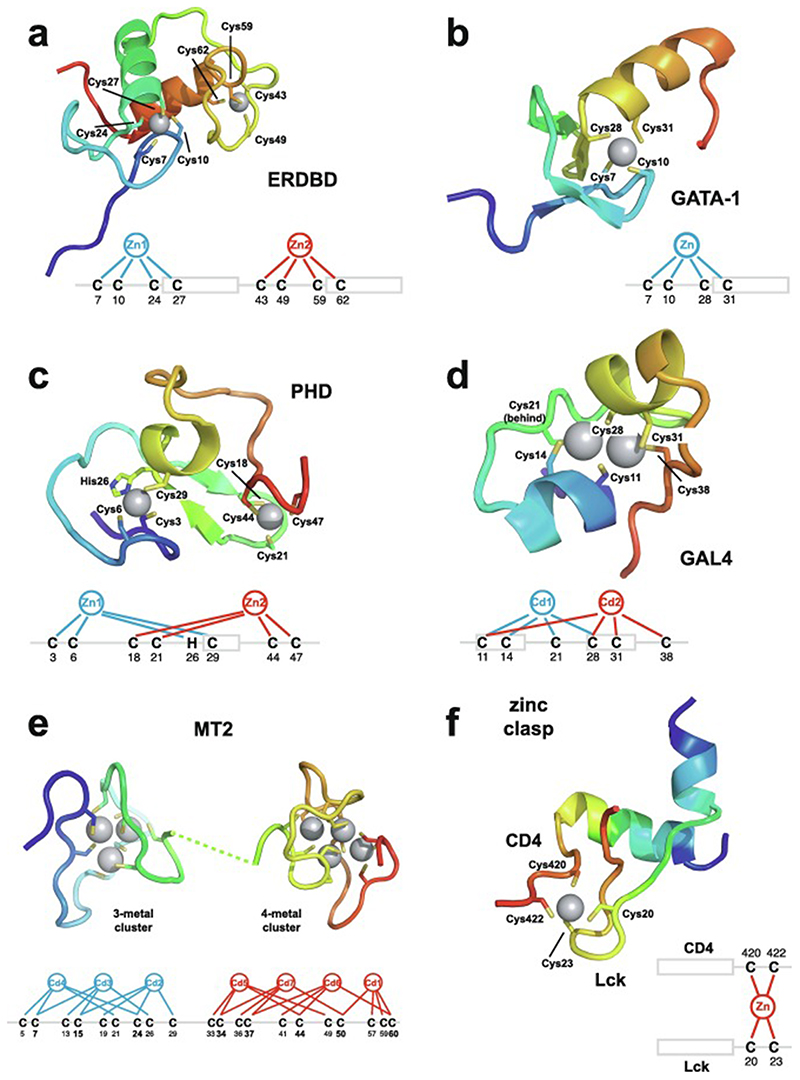
A selection of other types of zinc finger and related structures. The examples shown are chosen to illustrate some of the many different metal-binding topologies that can be found amongst published structures. **a**) NMR structure of oestrogen receptor DNA-binding domain (ERDBD), one of the first non-canonical zinc finger protein structures to be published (PDB 1hcp) [27]. **b**) GATA-1 DNA-binding domain. The protein is a chicken erythroid transcription factor that takes its name from the cognate DNA sequence it recognises, and is an early example of the so-called “treble-clef” motif [32,39]; the structure shown is taken from that of the complex with DNA (PDB 1gat)[33], which was amongst the first protein:DNA complexes to be determined by NMR. **c**) NMR structure of the plant homeodomain (PHD) zinc finger from human Williams-Beuren syndrome transcription factor (PDB 1f62) [40]. This structure illustrates a so-called “cross-braced” or “interleaved” metal-binding topology found in several types of two-metal finger domains, in which one metal is bound by the 1st, 2nd, 5th and 6th ligands and the other by the 3rd, 4th, 7th and 8th ligands; this contrasts with the “sequential” metal-binding topology seen for ERDBD in panel a). See also Fig. 10 for further examples of this important distinction. **d**) DNA binding domain of the yeast transcription factor GAL4, showing how a two-metal cluster is formed with six cysteine ligands, two of which (the “bridging” cysteines) are shared between the two metals. The structure shown comes from a crystal structure of the complex of Cd_2_ GAL4 with DNA (PDB 1d66) [37]; two NMR structures were also independently determined in the same year (1992) [35,36], but are not present in the PDB database. **e**) NMR structures of the three- and four-metal clusters from Cd7 rat metallothionein 2 (MT2) (PDB 1mrt and 2mrt) [38]. Metallothioneins are not generally considered to be zinc finger proteins, but are discussed in this review because much of the NMR methodology for using ^113^Cd substitution to establish metal-binding topology was first developed during studies of metallothioneins (see Section 5.2). **f**) Intermolecular “Zinc clasp” linking the N-terminal tail of the tyrosine kinase Lck to the cytoplasmic tail of the T-cell co-receptor CD4 (PDB 1q68) [28]; this zinc-dependent tethering is required during T-lymphocyte development. Smoothed protein backbone cartoons were produced using the program PyMol [23,24] and are shown in “chainbow” colouring (blue → red, N-terminal → C-terminal), zinc ions are shown as grey spheres and all the NMR structures are illustrated using only the lowest energy member of the deposited ensemble in each case. In each case, the sequence numbers shown are those in the deposited PDB files, which may not necessarily correspond to the numbering of the full-length native protein. In the case of metallothionein, numbering is omitted from the structures to avoid overcrowding; on the schematic, numbers for bridging cysteines are shown in larger, bold type, and the cadmiums are numbered in order of decreasing ^113^Cd chemical shift (see Fig. 19a).

Very soon after the original proposal of the zinc finger motif in TFIIIA, it became clear that structural zinc ions occurred in many other proteins in very different structural contexts (for an excellent, more detailed discussion of the distinctions between structural and catalytic zinc sites, see Lee *et al*. [25]). The first such non-TFIIIA-type zinc finger structures to be determined were those of the DNA-binding domains of two steroid hormone receptors, the glucocorticoid [26]and oestrogen [27]receptors, both published in 1990, which were each independently found by NMR to be structures of around 80 amino acids containing two zinc ions, each tetrahedrally co-ordinated by four cysteines (Fig. 2a). Structures of other domains involving structural zinc started to appear, and it quickly became clear that there was a large repertoire of possibilities. A single zinc could be co-ordinated by different combinations of cysteine and histidine residues (typically denoted C_2_H_2_ or CCHH, CCHC, C_4_, etc., depending on the distribution of Cys and His residues) that could occur in different secondary structural contexts, and in addition many domains contained more than one zinc. Multiple zincs could be present at independent sites (i.e. sites each having their own discrete set of four zinc-binding ligands), or in clusters where one or more zinc-binding ligands (invariably Cys) is shared between two zincs (e.g. Fig. 2d and 2e), while in still other cases zinc could be bound by ligands on two separate protein chains, e.g. in a “zinc clasp” (Fig. 2f) [28]or “zinc hook”[29]. Occasionally, aspartate has been found as one of the zinc ligands [30,31] (N.B. here “occasionally” refers specifically to “structural” zinc sites; co-ordination by Asp and/or Glu residues is common in catalytic sites containing zinc). An excellent review by Krishna, Majumdar and Grishin appeared in 2003 that presented a careful structural classification of the structures known at that time [32], bringing some much-needed order to what might sometimes have seemed to be increasing chaos. Many more structures have appeared since then, but the general principles set out in the Krishna review are still extremely useful. Fig. 2 shows a (very) small selection of structures of non-TFIIIA-type zinc fingers, chosen mainly to illustrate the wide variety of metal co-ordination topologies that can occur [26–28,33–38]. However, this is not intended to be any more than just the tip of the iceberg; to attempt an updated, comprehensive coverage of the great diversity of currently known zinc finger structures would require a review completely different from this one (and from a different author).

In the light of this abundance of very varied structures, it is perhaps worth asking what the term “zinc finger”actually means now. For some time after their initial discovery, it was hoped by some that the term zinc finger would be reserved for TFIIIA-type fingers, thereby preserving the appealing structural analogy with a hand grasping onto DNA for the case of sequence-specific recognition. However, it seems that the name itself was too good to remain tied down in this way. Although views may still differ in private as to exactly what it means, or ought to mean, in practice the name “zinc finger” has now come to be widely used for pretty much any small protein domain that is built around one or more structural zincs (and this is the way in which the name will be used in this review). This also means there is no longer any specific link with DNA binding. Such a link was in any case never absolute; for instance, the prototypical zinc finger protein TFIIIA itself binds to either DNA or RNA as part of its function [1], and GATA proteins contain two zinc fingers, one that binds DNA and a second that binds a protein (intriguingly named FOG, for “friend of GATA”) [41]. Attempts to use the presence of zinc fingers as a diagnostic for DNA binding were always dangerous, and soon had to be largely abandoned in light of the very many different functions of zinc finger proteins that began to populate the literature, involving numerous examples not only of binding to DNA but also to other proteins, to RNA, and to other biological molecules. There has been occasional speculation that the presence of zinc might itself have functional significance, for instance as a redox switch [42,43]. However, a more general principle (in this author’s view) would seem to be that the role of zinc in these proteins is in large part analogous in the intracellular context to that of disulfide bonds in the extra-cellular context. Disulfide bonds are generally unstable under the reducing conditions present within most cells, while zinc coordination by cysteines would not be expected to survive under the oxidising conditions generally found in extracellular space, so it makes sense that the function of structural reinforcement would be carried out differently in the two environments [44]. Indeed, one may make a strong case that tetrahedral co-ordination of zinc provides a still more effective means of structural rigidification than does the single, relatively flexible linkage provided by disulfide bond formation.

It is noticeable that a significantly higher proportion of zinc finger structures have been determined by NMR, as opposed to X-ray crystallography, than is the case for many other types of proteins, at least in their free states. Reasons for this are not hard to find. Many of the structures are small, and in the case of multiple-finger fragments they are very frequently flexibly linked, making them resistant to crystallisation. When studying fingers “excised” from a larger protein context (as is very frequently required for such structure determinations), it may also be difficult to assign domain boundaries a *priori*, meaning that fragments may have significant disordered N- and/or C-terminal tails (see also Section 3.5), which again can make crystallisation more difficult. Also, zinc fingers, particularly the smaller types, may have only marginal stability, potentially causing further difficulties during long crystallisation trials. In the case of crystallography, many of these problems may be alleviated by studying zinc fingers in complex with their binding partner (s), particularly for those that bind sequence-specifically to double-helical DNA, as this may quench flexibility and increase stability; this of course has the huge added benefit that it reveals important details of the binding interface and hence the mechanism of recognition. Sadly, while studying a DNA complex, when possible, may make life easier for crystallographers, the same cannot be said for NMR spectroscopists; there have been a number of NMR structure determinations of complexes of zinc finger proteins with DNA (e.g. [17,18,33]), RNA (e.g. [45]) and other proteins (e.g. [41]), but these have each represented a much more significant undertaking than working with the free proteins. Where NMR often retains a clear advantage, however, is in cases where the binding partner of a novel zinc finger protein remains unknown, or where a complex cannot be crystallised.

In this review I have attempted to draw together the various considerations that apply particularly to determining NMR structures of zinc finger domains, beyond those that apply to any protein structure determination by NMR. These include not only the relevant NMR experiments and their interpretation, but also important related issues that need to be taken into account if planning such a study, such as sample characterisation and handling, as well as structure calculation and analysis. However, in order to limit the scope, I have deliberately not included discussion of zinc finger interactions with their recognition targets.

**Figure 3:**
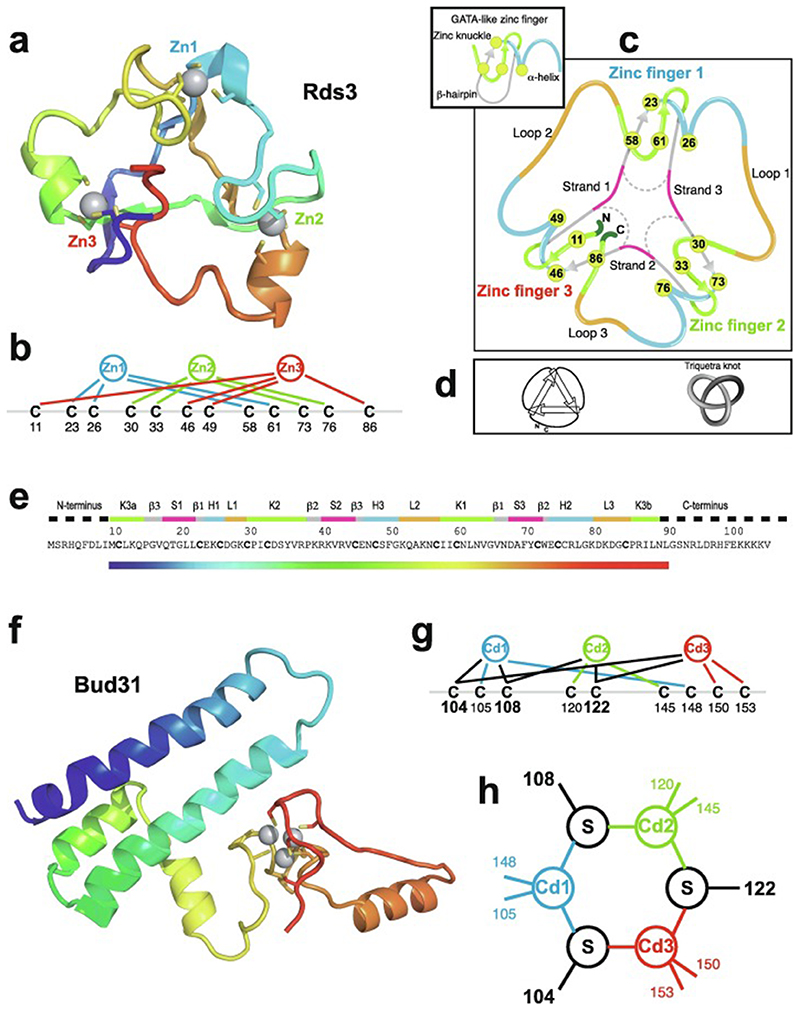
Solution structures of the proteins Rds3 and Bud31. **a**) Cartoon representation of the lowest energy structure of the deposited NMR ensemble of Rds3 (PDB 2k0a). **b**) Metal-binding topology of Rds3; in this structure, the three zinc-binding sites are relatively well spaced apart and are independent (no ligands bridge between zincs). **c**) Schematic showing how Rds3 is built from three GATA-like zinc fingers (each consisting of a β-hairpin, a zinc knuckle and an α-helix, see inset) fused together. In Rds3, each b-hairpin is broken and replaced by a strand (shown in magenta) running from one finger to the next, and a loop (shown in orange) is added from the α-helix of one finger to the zinc knuckle of the next. The zinc knuckle of finger 3 is “interrupted” by the N- and C- termini of the protein chain. **d**) Simplest representation of the origin of the topological knot in the structure of Rds3 (shown in the same orientation as in a) and c)). **e**) Sequence of yeast Rds3; components of the structure are shown above the sequence using the same colour code as in c) (K, zinc knuckle; b, b-hairpin; S, strand; H, helix; L, loop; numbers denote fingers 1, 2 and 3). The colour gradient bar below the structure shows the “chainbow” colouring used in a). **f**) Cartoon representation of the lowest energy structure of the deposited NMR ensemble of Bud31 (PDB 2my1). **g**)Metal-binding topology of Bud31, displayed as function of sequence; as discussed in Section 5, zinc was exchanged by ^113^Cd to help determine this topology. In this structure the three metals form a single cluster bound by nine cysteines, of which three (numbers in bold,connections in black) bridge between two metals. h) Metal-binding topology of Bud31, displayed based on the metal cluster arrangement. Protein backbone cartoons in a) and f) were produced using the program PyMol [23,24] and are shown in “chainbow” colouring(blue → red,N-terminal → C-terminal;a few disordered residues at the N- and C- termini are omitted for clarity in each case),zinc or cadmium ions are shown as grey spheres and the NMR structures are illustrated using only the lowest energy member of the deposited ensemble in each case. Panels a), c), d) and e) adapted from[48] (© 2008 National Academy of Sciences).

An important factor that underlies these NMR studies is that the zinc in zinc fingers is itself essentially NMR silent. Zinc does have an NMR-active isotope, ^67^Zn (see Table 2), but this is a very rapidly relaxing, strongly quadrupolar isotope, and is consequently far from trivial to observe. There have been variable temperature, wide-line, solid-state NMR studies of ^67^Zn in proteins that have been interpreted in terms of the detailed electronic nature of the direct bonds to the zinc ligands [46,47], but such experiments play no role in determining new atomic resolution structures of zinc finger proteins. Sometimes important information can be gained via substituting the zinc ions by other metal isotopes with more amenable NMR properties, most commonly ^113^Cd (see Section 5.2), but more frequently information about the zinc co-ordination has to be inferred indirectly by other means and then incorporated into structure calculations; much of this review is concerned with how this may be done in practice, and with what consequences. While the review is intended to be as general as possible, it has been convenient to use two particular examples from the author’s own laboratory, namely the yeast proteins Rds3 [48] and Bud31 [49], to provide detailed illustrations of some of the points. Both of these proteins are components of the spliceosome, the large and complicated molecular machine responsible for the process of mRNA splicing that removes introns from initial RNA transcripts to form a mature message for protein expression; the structures of Rds3 and Bud31 are shown in Fig. 3. In addition, much of the early work on developing ^113^Cd NMR for determining metal-binding topology was carried out during studies of metallothioneins. Even though metallothioneins are not normally classified as zinc fingers, and were known and studied long before zinc fingers were discovered, their metal-binding characteristics share many features with those of zinc fingers (for instance see Fig. 2e), so where it is relevant for describing the methodology they will also be included in this review. All the work covered in this review concerns solution NMR studies, since to the best of the author’s knowledge, to date no structures of zinc finger domains have been determined using solid-state NMR.

## 2 How do you know if you have a zinc finger?

One of the first questions when planning a structure determination for a suggested zinc finger protein is whether or not it actually contains any structural zinc ions; in other words, is it or is it not a zinc finger at all? The following two sections discuss approaches to this issue from theoretical and experimental starting points.

### 2.1 Sequence alignment

Generally, the first hints that one may be dealing with a zinc finger come from multiple sequence alignment data. Some of the best-known families of zinc fingers have very highly conserved and easily recognised sequence motifs that typically comprise the set of zinc-binding ligands themselves as well as a few other key residues involved in forming elements of the structure such as turns or a hydrophobic core (if there is one). The best known example of this is the TFIIIA-type C_2_H_2_ zinc finger, the canonical form of which is characterised by the sequence motif Cys-X_2-4_-Cys-X_3_-Φ-X_8_-His-X_3-5_-His (see https://prosite.expasy.org/PDOC00028), where Φ represents a hydrophobic residue. Even in cases where there are small deviations from this pattern, such as small changes in ligand separation, one can be reasonably certain that the presence of such an arrangement establishes that the sequence binds a single zinc ion and folds in a similar fashion to other TFIIIA-type zinc fingers. Other well-known examples, though not always so definitively identifiable from sequence data alone, include the GATA (https://prosite.expasy.org/doc/PS00344), RING (https://prosite.expasy.org/doc/PS00518) and PHD (https://prosite.expasy.org/doc/PS50016) finger types, among others. However, in many cases sequence variations, particularly those involving the lengths of the longer intervening sequences between ligands, can be quite large, with the result that classification into one of these families only becomes secure once the structure is known. This has been reflected in trends in the literature over time; while purely sequence-based classification of zinc fingers according to “consensus sequences” was quite a prominent feature of early papers, once a greater variety of structures became known systematic approaches to classification largely shifted to analysing structural features such as metal-binding topology and the locations of secondary structural elements [32].

One consequence of this situation is that it is quite common at the start of an investigation with a protein of unknown structure for sequence analysis to reveal a strongly conserved pattern of Cys and sometimes also His residues within a multiple sequence alignment that suggests these residues may be involved in zinc binding, but does not necessarily establish what type of zinc finger may be involved. In this context, a subtly different question may usefully be asked during analysis of the sequence data: not so much “what structural family of zinc fingers does this sequence alignment most resemble” (a topic that would require a much more extensive discussion than given here), but rather “what can this sequence alignment tell me about how likely it is that this protein really does bind zinc”?

The key point here is that for a structural zinc site to be identified, all four zinc-binding ligands must simultaneously be present in all the aligned sequences. This is a much stronger condition than the usual consideration of “strong conservation”. Firstly, there is the condition that it is only Cys or His (or very occasionally Asp) residues that are permissible as ligands, rather than allowing “near-misses” of residues with approximately similar properties as acceptable matches, as is normal practice in most other contexts during sequence analysis. But it is the second condition, that every zinc site must have a full complement of four ligand residues in every sequence, that provides the most discrimination, since this is very unlikely to arise by chance. Depending on the structural context, it might happen that a ligand residue could occasionally shift its position very slightly between aligned sequences, though given the requirement for all of the ligand residue side chains to be correctly oriented towards the zinc, even this degree of variation likely requires some structural shifts (and sometimes a change between Cys and His for one or more ligands). However, the occurrence of any non-ligating residues at one or more of the proposed ligand positions in one or more of the aligned sequences must in general cast doubt on the hypothesis that a zinc-binding site has been located. Assuming no sequencing errors, such discrepancies likely imply either that the proteins in which they occur do not belong in the alignment (i.e. they have a different structure), or else that the site is not real. That said, it is possible that a protein fold may evolve away from the requirement for a zinc site, instead deriving its stabilisation from non-covalent interactions such as hydrogen bonding, salt bridge formation and Van der Waals inter-actions. Such a situation has been described for natural proteins closely related to the RING domain [50,51] (it also worth noting that small non-natural domains have been designed that mimic the TFIIIA zinc finger fold without binding zinc [52,53]).

**Figure 4:**
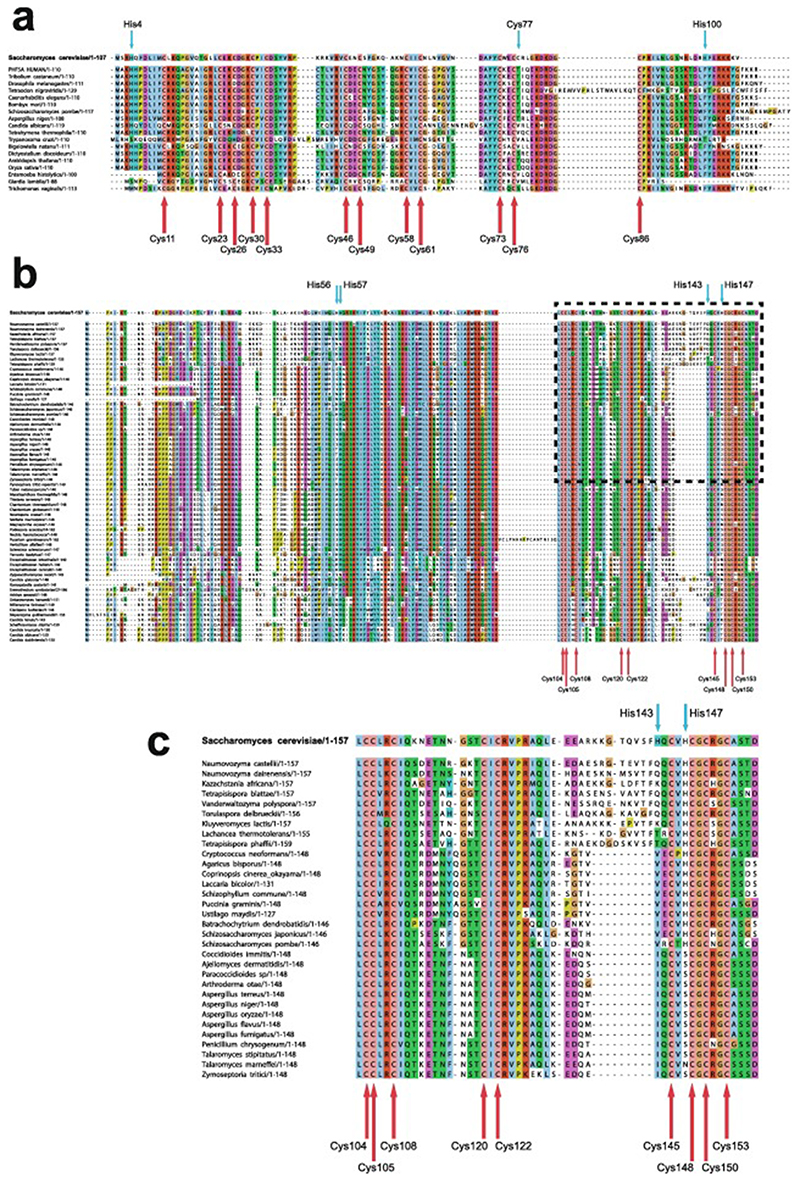
Examples of multiple sequence alignments for zinc finger proteins. Multiple sequence alignments for the yeast proteins **a**) Rds3 and **b**) Bud31; the region inside the dashedboxofpanel **b**)is expanded in **c**).Metal-binding residues,which are all cysteines in both of these examples, are indicated with red arrows beneath, and are absolutely conserved throughout. Other residues that might have appeared to be candidate metal-binding residues, based on inspection of the yeast sequence in isolation, are indicated by smaller cyan arrows above; in each case they may be ruled out by the lack of conservation seen in the alignments (His4 in Rds3 and His57 in Bud31 are considered further in Section 5.1). The alignment shown for Rds3 includes a small selection of very diverse eukaryotic species,while that for Bud31 is restricted to fungi, though a more extensive alignment across eukaryotes (not shown) is also fully consistent with the conclusions regarding metal-binding residues made here. No overall residue numbering scale is shown as each protein has its own distinct native numbering, and as is normal for multiple sequence alignments the sequences are not represented continuously so as to accommodate insertions in a subset of sequences. The alignments were produced in the program ClustalW [54]; panel a) is adapted with permission from [48] (© 2008 National Academy of Sciences), panels b) and c) are courtesy of Dr. Antonina Andreeva, MRC LMB.

In the case that there are four, and only four, completely conserved ligand residues found within a reasonably extensive sequence alignment for a particular domain, then it is quite likely that this indicates that the domain contains one structural zinc at a single binding site; the commonest ligand arrangements in such cases are C_4_, CCHC, CCCH and C_2_H_2_. However, once the number of potential ligands exceeds four then other possibilities may need to be considered, leading to a hierarchy of further questions. How many zinc ions are bound? If more than one, which ligand binds to which metal? Are any of the Cys residues shared between two zincs? Are any of the potential ligands conserved for other reasons, i.e., are they in fact not involved in zinc binding? These questions may or may not be answerable by analysing sequence alignments alone, and more often require additional information from other sources, such as knowledge of the zinc-binding stoichiometry or even preliminary structural information. We will return to these important questions in later sections, particularly Sections 2.2 and 5. Multiple sequence alignments for the example proteins Rds3 and Bud31 are shown in Fig. 4, highlighting some of the points just discussed.

Another useful bioinformatic resource (although not directly linked to protein sequence) is the database “ZincBind” (zincbind.net) introduced in 2019, which identifies and collects details of all known zinc-binding sites in the PDB, and which can be helpful when assessing whether a possible array of potential ligand residues is likely actually to bind zinc, and/or has been seen previously [55]. Sites are included regardless of whether or not they are “structural” as defined here (i.e. four ligands comprising a combination of Cys, His or Asp residues). Thus, the database includes a large number of very diverse but, in many cases, sparsely popu-lated families of mainly “enzymic” sites involving many different types of ligand residues; in contrast, the number of families corresponding to “structural” zincs is quite small, but each is highly populated.

### 2.2 Experimental tests for bound zinc; zinc stoichiometry

Unless sequence analysis unequivocally reveals the zinc-binding arrangement in a new protein thought likely to be a zinc finger, it will usually be necessary to establish whether or not it actually does bind zinc ions, and if so how many, by experiment. This section discusses the main methods that have been used to achieve this (related questions such as the strength of zinc binding, or how zinc is introduced into the protein either *in vitro* or *in vivo*, are included in Section 3.1).

Very often the details, or indeed any mention at all, of the methodology used for zinc content determination are quite deeply buried in the experimental or supplemental sections of a paper mainly concerned with structure determination or other aspects of initial characterisation of the protein. This can make it quite difficult to bring together an overall picture of which methods are popular and/or effective. What follows therefore makes no pretence at being comprehensive; rather than attempting to catalogue all instances of all approaches the discussion is intended to be illustrative, while emphasising those methods that are the most general and useful.

That said, before discussing methods that work well it may be useful first to mention some disadvantages of what might naïvely appear the most obvious method, since this may highlight factors important to the success of other approaches. By analogy with countless NMR-based studies of small molecules binding to proteins, one might suppose that titration of metal ions into a solution of the apo-protein while monitoring chemical shift or intensity changes (depending on the exchange rate between free and bound states) would generate a binding curve from which metal binding could be estimated. However, a number of factors militate against this approach. A key issue would be generation and stability of the apo-protein, from which the native zinc ions have been stripped out, as this may be considerably less stable than the metal-bound form. Depending on the proportion of the total folding energy of the native protein that comes from zinc binding (which in turn depends on the architecture of the structure), the apo-protein often lacks some or all of the folded structure of the native protein, and consequently may be prone to aggregation and precipitation, oxidation of free cysteines, and/or attack by traces of co-purified proteases if any are present in the solution. In the case of an NMR titration the requirement for apo-protein stability would be relatively severe as the experiment would likely last for several hours, and the interpretability of results would be severely compromised if the apo-protein concentration were depleted over time by such processes. Another problem, particularly for proteins that bind more than one zinc, would be that folding by adding metal to apo-protein in vitro may not cleanly yield only the native state, but rather be complicated by potentially irreversible competing pathways involving incorrect metal-binding topologies and leading to varying degrees of misfolding, aggregation and precipitation. Additionally, one would of course need accurate values for the concentrations of both the protein and the metal throughout the titration, which would need to be conducted using a single buffer suitable for both apo- and native proteins simultaneously, with the further requirements that the protein solution be sufficiently concentrated for convenient NMR measurements and completely free of zinc at the start. All this is before any consideration of the influence on the spectra of metal exchange leading to interconversion between the different forms, which is likely to be very slow on the chemical shift timescale as zinc binding in most cases is very strong (see also Sections 3 and 5.2).

Given that NMR titration is likely to be a poor approach, what approaches are actually useful for determining the metal content of zinc fingers? Since its introduction to the study of proteins in the early 1990^0^s, the most commonly used method has been mass spectrometry, using the mass difference between native and apo-protein to establish the number of bound zincs (or cadmiums, when relevant). This is a relatively rapid technique, requires very small sample quantities, establishes not just the presence but also the identity and stoichiometry of the metal(s), does not require long-term stability of concentrated apo-protein samples or accurate concentration measurements, and allows apo- and native protein samples to be handled in independently chosen buffers (though subject to the requirements of compatibility with the mass spectrometric technique, the most restrictive of which is often a requirement for the absence of sodium ions). Amongst the first such applications was a study of zinc and cadmium binding by metallothioneins using electrospray ionisation (ESI) mass spectrometry published in 1993, which demonstrated the crucial point that bound metals are partially or completely retained by the protein in the gas phase during analysis [56]. This approach has been widely adopted since then; a selection of examples can be found in references [10,35,45,48,49,57–59], and applications, including to zinc fingers, have been reviewed [60–62]. Intriguingly, one paper reports an accurate mass study of C_4_, CCHC and C_2_H_2_ zinc fingers, which the authors interpreted as showing that in each case two cysteines are deprotonated in the native protein while any further cysteines involved in the binding remain protonated such that the zinc site retains formal charge neutrality overall (i.e. C_4_ fingers retain 2 SH protons, CCHC fingers retain one, and C_2_H_2_ fingers none) [63]. However, the relevance of this is unclear since a) mass spectrometry cannot establish the actual sites of proton gain or loss, only the net change in number across the whole molecule (which is itself a consequence of maintaining the same charge state across the different peptides chosen for comparison), and b) all the peptides in this study were based on a consensus TFIIIA-type finger (CP-1 [64], see Section 3.1), so the C_4_ and CCHC sites are far from being typical natural sites for such a co-ordination scheme, and zinc binding by the non-canonical cysteines in the α-helix may be compromised by inappropriate geometry. To the best of the author’s knowledge, co-ordinates of protons bound to Sγ of zinc-binding cysteines are never present in deposited NMR structures of zinc finger proteins (of course the issue does not normally arise for X-ray or EM structures of zinc fingers or their complexes, since usually only heavy atom co-ordinates are deposited for such structures).

**Figure 5:**
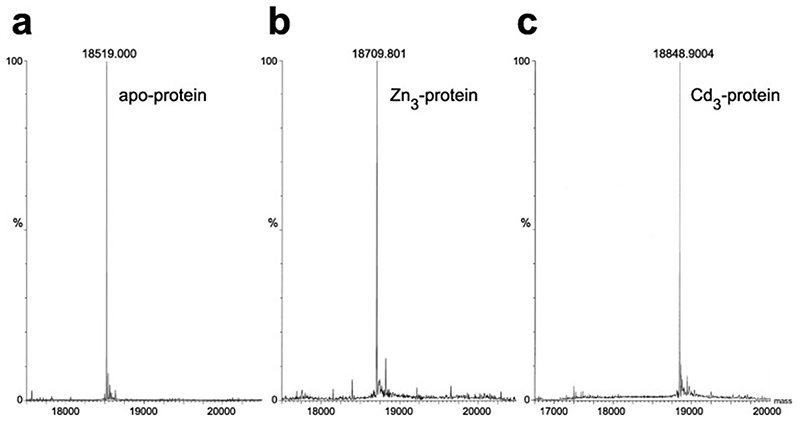
Mass spectra used to determine metal content for a zinc finger containing a three-zinc cluster. Mass spectra are shown for the yeast protein Bud31 under denaturing and non-denaturing conditions, to demonstrate the metal content. **a**) Bud31 under denaturing conditions without metal bound (principal peak 18519.000); **b**) Zn3 Bud31 measured under non-denaturing conditions (principal peak 18709.801); **c**) ^113^Cd3 Bud31 measured under non-denaturing conditions (principal peak 18848.9004). The mass difference between the zinc and the cadmium species proves that all three zincs have been cleanly exchanged for cadmium in this instance, with no intermediate species containing both zinc and cadmium present in the final sample. Adapted with permission from[49] (CCBY licence, ©2015 van Roon et al.).

Fig. 5 illustrates mass spectra used to determine the metal content of Bud31; these measurements were important in establishing the success of metal replacement of Zn by ^113^Cd in this case (see also Section 5.2).

Several other approaches to metal quantification have been used with zinc finger proteins, particularly in the first few years after their discovery. A common method has been to use atomic absorption spectroscopy to quantify the zinc content; this of course also requires accurate knowledge of the protein concentration, to allow calculation of the stoichiometry from the ratio of zinc to protein concentrations. The zinc content of the first described zinc finger protein, TFIIIA, was estimated in this way [1], though it is worth noting that, despite the utmost care being taken as described in the original paper, the zinc content was underestimated in this case. Comparison of the zinc and protein concentrations, both measured for 7S particles (which as isolated from Xenopus laevis oocytes are complexes of the 5S RNA genes with full-length TFIIIA protein), yielded a stoichiometry of 7.0 ± 0.5 Zn per particle, whereas it was clear from the repeating motif in the amino-acid sequence that the actual number of zincs per TFIIIA molecule should be nine. Later studies with other proteins such as glucocorticoid receptor DBD [65], LIM domain [66], RING domain [67] and PHD domain [34] were somewhat more tolerant of errors in the zinc concentration, since the stoichiometric ratios involved were lower (two zincs per protein in these cases) and protein concentration estimation was likely more accurate, being based on amino-acid analysis. More recently, a popular and accurate approach for obtaining the Zn:protein ratio, both for zinc fingers (see e.g. [68]) and metallothioneins (see e.g. [69,70]), has been to use inductively coupled plasma – optical emission spectroscopy (ICP-OES) to determine sulphur and zinc content simultaneously.

A further strategy is to replace the zinc by a different metal that can be quantified using a different form of spectroscopy. For instance, although it binds more weakly than does zinc, cobalt can occupy the metal-binding sites in many zinc fingers, giving rise to a UV/vis spectrum characteristic for Co^2+^ in a tetrahedral ligand field environment; this can be exploited to estimate the stoichiometry either by titrating Co^2+^ into apo-protein [71] or by monitoring loss of the characteristic UV/vis spectrum during displacement of Co^2+^ by addition of Zn^2+^ [4,8]. As mentioned earlier, zinc can often also be replaced by cadmium, which binds with comparable or greater affinity to that of zinc [72], allowing characterisation of the metal binding using ^113^Cd NMR [66,73]. Depending on the NMR experiments deployed, this can potentially reveal much more information than just stoichiometry, as is discussed in Section 5.2.

Other methods employed to investigate metal content include a number based on various X-ray measurements (other than crystallography), though in these cases the information sought is often more to do with characterising the nature of the directly metal-bound ligand atoms than counting the metal atoms themselves. Several early studies used Extended X-ray Absorption Fine-structure (EXAFS) to demonstrate the presence of Zn-S and, where relevant, Zn-N bonds in the structures (e.g. [65,74–77]) and in one case also in intact viruses, thereby demonstrating that they contained zinc finger proteins [76]; such EXAFS studies were also invoked (e.g. [3]) to provide bond length data that were incorporated into the force fields used to calculate NMR structures of zinc fingers (see Sections 4.1 and 4.3). More recently, other X-ray based approaches have been recommended for use in quantifying and characterising protein-bound metals. Micro-beam Proton-Induced X-ray Emission (PIXE) [78,79]can be used to analyse for many ele-ments in protein samples, and has been used to determine zinc content in at least one NMR study of a new zinc finger structure [80,81]. Like EXAFS it works with liquid samples, but unlike EXAFS it has the important advantage of being quantitative. It requires access to a suitable facility based around a proton accelerator, though its advocates state that this is a less restrictive condition than the synchrotron access needed for EXAFS. Others promote the use of Energy Dispersive X-ray Fluorescence spectroscopy (EDXRF) [82], which is also quantitative and works with thin film samples using lab-based equipment, but to the extent of the author’s knowledge applications to zinc finger proteins in structural studies have not yet been reported.

Simpler, more qualitative tests for zinc have also been used, particularly in relatively early studies. For instance, demonstration that treatment with a zinc chelator destroys some aspect of a protein’s function, such as its ability to bind an interaction partner, e.g. DNA, has been used as part of the argument that a zinc finger is involved (e.g. for PARP-1, the protein that acts as the main detector of DNA single-strand breaks in eukaryotes [83,84]), while zinc removal using EDTA followed by reconstitution of the holoprotein by removal of EDTA followed by adding back Zn^2+^ was used to demonstrate zinc binding during structural studies of a PHD finger [40]; simple removal of zinc using EDTA has also been used in a very recent study [85]. However, some zincs are bound more tightly than others (see also Sections 3 and 5.2), and for several proteins (e.g. GATA-1 [86], and a different PHD finger [34]) there have been reports that attempted zinc removal using EDTA failed. 1,10–Phenanthroline has been recommended as a superior chelating agent [87], but in at least one case this too was reported to fail [86]. It is difficult to know to what extent some of these differences reflect genuinely different zinc-binding affinities of the various proteins, or differences in the protocols employed for zinc removal by different research groups. Zinc removal has also been used to determine zinc content more quantitatively, by chemically modifying cysteines with various aromatic organomercury compounds (e.g. *p*-mercuribenzenesulphonate [88], *p*-hydroxy-mercuriphenyl sulphonate [89]or *p*-hydroxy-mercuribenzoic acid [90]) and then estimating the released zinc using a metallochromic indicator, 4-(2-pyridylazo)resorcinol; it is likely that other papers include similar approaches.

Some studies use NMR chemical shift data to identify which Cys and, when relevant, His residues are bound to zinc, although this is not guaranteed always to be unambiguous. This topic will be discussed further in Section 5.1.2.

## 3 Sample stability and handling for solution NMR

Protein samples for solution NMR need to be stable for extended periods of days or weeks, or sometimes even months if one is to be able to carry out a comprehensive set of experiments all using the same sample for complete consistency. This requirement is no different in the case of zinc finger proteins than it is for others. However, there may be additional points that are important in satisfying it where samples of zinc fingers are concerned, and it is these in particular that are discussed in this section.

### 3.1 Metal binding

One key consideration that underlies much of what follows is the strength with which the metal is bound, and while it is not necessary to know the metal-binding affinity in order to carry out an NMR structure determination, it can be very useful to be aware of the generalities of metal binding. Metal-binding affinities for zinc finger proteins are far from trivial to measure, and there is an extensive literature both on the various methods that have been applied and on the considerable variety of values they have yielded for different zinc finger proteins. Excellent recent reviews have been published from the Krężel group covering the area in considerable depth [91,92]; since a detailed account would be outside the scope of the present review, a few of the most relevant points will be summarised here:

i) Reported dissociation constants for zinc in natural zinc fingers cover a huge range, from sub-micromolar to attomolar; the factors that cause this very wide variation are only partially understood.
ii) Some commonly used methods of measurement, particularly in the earlier literature, can severely underestimate the strength of binding for tightly bound zincs, even by several orders of magnitude; it may be desirable to use more than one method to establish an accurate value.
iii) Many natural canonical (i.e. TFIIIA-type) zinc fingers typically have zinc dissociation constants in the approximate range 10^-11^ M – 10^-13^ M (after taking point ii) into account).
iv) Data for other types of natural zinc fingers are more sparse, with less known about the range of dissociation constants.
v) Mutant zinc fingers may bind zinc much less tightly in cases where mutation disrupts the structure, whereas some “designed” zinc finger proteins, in which several stabilising features have been brought together artificially (e.g. the “consensus peptide” CP-1 proposed by Berg [64]) may bind much more strongly, with zinc dissociation constants as tight as roughly 10^-16^ M [93].
vi) In all cases, the strength of zinc binding is very strongly dependent on the solution conditions, particularly pH. In general, protonation of Cys sidechains renders them ineffective in metal binding, so the lower the pH the more likely it becomes that bound zinc will dissociate (see Section 3.2).

As an aside and to give some context, it is interesting to consider briefly how some of these numbers relate to the natural intra-cellular environment in which zinc finger proteins function. Another paper by Krężel [94] presents an interesting account, and includes references to earlier papers by others. To summarise: in the case studied (human colon cancer cells, HT-29), it was reported that the total zinc concentration was 264 μM, with the overwhelming majority of this zinc being bound to proteins, while the concentration of “free” zinc, i.e. zinc available for binding to vacant binding sites on proteins, was tightly regulated in the region of 1 nM (depending also on the state of the cells). This regulation of “free” zinc is achieved, at least in part, by metal-dependent expression of thionein (i.e. apo-metallothionein) [95], which is up-regulated in response to higher levels of metals, thereby providing a buffering sink. Given the affinities discussed above, it is clear at the thermodynamic level that such concentrations of intracellular “free” zinc are compatible with the zinc sites on most natural zinc finger proteins being consistently fully bound inside cells. However, there is probably much yet to be discovered about the kinetic mechanisms that ensure that zinc is appropriately and rapidly delivered to the multitude of protein sites that need to be occupied for those proteins to be functional.

Returning to the hopefully simpler (though not necessarily better controlled!) environment of the NMR tube, the foregoing discussion makes clear some of the factors that determine the stability of zinc finger samples for solution NMR. As initially isolated following overexpression in bacteria or other cells, samples of zinc fingers will generally be fully saturated with zinc, and should remain so during purification unless treated with a strong chelating agent such as EDTA (and even then, some may retain their zinc, see Section 2.2), provided they are not exposed to low pH. It is quite common for NMR samples of zinc fingers to be prepared including an excess of a soluble zinc salt, usually Zn_2_SO_4_ or ZnCl_2_, with the aim of helping to keep the zinc sites on the protein fully saturated. While this presumably does no harm (other than making it important to ensure there are no other buffer components present that would combine with the zinc ions to form precipitates; see also Section 3.4), given the strength of zinc binding by the protein one might also argue that it probably does little good once isolation and purification have been completed. Indeed, avoidance of the need to titrate in zinc ions to saturate the binding site(s) is one key advantage of preparing zinc finger samples using cellular over-expression (although some protocols do employ refolding of over-expressed protein in the presence of zinc ions, e.g. [96]).

A few studies, particularly some of smaller zinc fingers, have instead used chemically synthesised peptides (e.g. [97]), and in these cases it is necessary to add zinc to the isolated peptide in order to reach the native state. This can be tricky, particularly with concentrated samples and for zinc fingers containing more than one metal-binding site; slowly mixing diluted solutions and then re-concentrating may be helpful in encouraging equilibration. Also, uncertainties in concentration estimates mean that it is likely to be difficult to match the concentrations of peptide and zinc exactly, with the result that if saturation of the binding site(s) is prioritised there will likely be a small excess of free zinc in the final sample. This may or may not matter; as just discussed, a small excess of zinc may often be deliberately added even to samples of over-expressed proteins. However, in the case of synthetic peptides the concentration of excess zinc following addition of a soluble zinc salt is likely to be less well quantified or controlled, which could become a problem if, for instance, the zinc finger sample, or perhaps an interaction partner, has one or more further zinc-binding sites (just such a situation existed for one recent study inthe author’s lab [81]).Given the strength with which most zinc fingers bind zinc, excess metal ions could probably be removed using a mild dialysis regime without disrupting the native finger, though the author is not aware of examples where this approach has been followed during purification of a zinc finger sample made from chemically synthesised peptide.

Of course, a key issue with metal dissociation is not merely that whatever proportion of the protein in the sample that is not bound to metal does not contribute to the folded spectrum. The bigger problem is that while the protein is not in the fully metal-bound state it is likely to unfold, and it then becomes vulnerable to other processes that may lead to degradation more quickly than they do for the folded state. While the long-term stability of samples can be highly variable and unpredictable, and much of the evidence around this is anecdotal in nature, it is fairly clear that factors such as proteolysis by traces of co-purified proteases will become worse for zinc fingers the less strongly the zinc is bound. However, there are other factors that can cause the zinc to be slowly, or occasionally not so slowly, released from the protein leading to sample degradation, and which should be taken into account when prepar-ing samples. Most notably these include pH and oxidation, as will be considered in the following two sections.

Finally in this section, it is interesting to note a recent report from Soni et al. that describes what the authors term “ambiguous” zinc co-ordination, whereby the presence of an additional cysteine residue, in excess of the number required for full metal co-ordination and adjacent in the sequence to a canonical metalligating residue, can exert quite profound kinetic effects by partic-ipating in transient, competitive binding to the metal [98]. The RNA-binding protein RBM5 contains a small (26 residue) C_4_ zinc finger domain that binds a single zinc, but which has an additional cysteine immediately following the second canonical zinc-binding Cys residue. Mutation of this additional Cys to Gly causes the rate of metal exchange to become approx. 55-fold slower, as established using a combination of real-time UV measurements and, in the case of the mutant, Zn – ^113^Cd exchange measurements monitored by NMR (as previously reported by Houben *et al.* for the proteins CNOT4 and p44 [72]; see also Section 5.2.1). The mutant was found to give simpler, higher-quality spectra and improved sample stability, which the authors interpret as being due to the removal of transient, destabilising effects caused by the additional cysteine. They suggest such mutations could also be useful in studies of other proteins where similar sequence features imply that ambiguous metal binding could potentially occur, and they speculate on possible reasons why such effects in the WT protein might be evolutionarily advantageous.

### 3.2 pH

**Figure 6:**
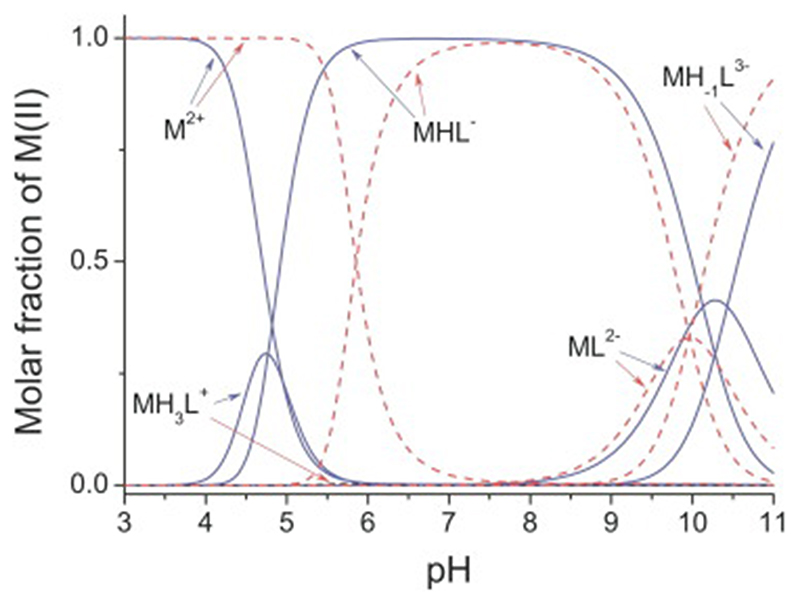
pH Dependence of metal binding for a TFIIIA-type zinc finger. The molar fractional distribution of Zn^2+^ (blue lines) and Co^2+^ (red dashed lines) complexes of the 11th zinc finger peptide of the human transcription factor ZNF133 as a function of pH; this peptide is referred to as ZF133-11 in the original paper, where the derivation of these curves using ionisation constants measured by a potentiometric titration for a 50 lM solution of the peptide is described [91]. The main point to notice in this plot is that the metal-bound complex (MHL^-^, see text) becomes unstable at low pH and releases metal ions as the S^c^atoms of the cysteine residues become protonated. Because cobalt binds more weakly than zinc, this process is complete at higher pH values for Co^2+^ than for Zn^2+^. See main text for further discussion. Reproduced with permission from[91] (© 2014 Oxford University Press).

The importance of pH is illustrated in Fig. 6, which shows the pH dependence of zinc and cobalt binding as measured for a TFIIIA-type zinc finger, the 11th finger from the human transcription factor ZNF133 [91,99]. The peptide sequence used in this instance (Ac-PMV**C**GE**C**GRGFSQKSNLVA**H**QRT**H**SGER) was chosen because it contains a particularly small number of ionisable groups aside from the metal binding site; during the titration, the only groups that change their protonation state are the two cysteines and two histidines of the metal-binding site and in addition just the two glutamates and a single lysine. In the central region of the plot the predominant species is labelled MHL^-^ (here M is Zn^2+^ or Co^2+^ and L is defined as the de-protonated ligand in which cysteines and glutamates are negatively charged and lysine is neutral; MHL^-^ thus corresponds to the complex in which the metal centre is overall neutral, comprising Zn^2+^ or Co^2+^ and 2 x thiolate S^-^, while the lysine is protonated with a charge of + 1 and both glutamates are deprotonated, each with a charge of – 1). The key feature to note here is that as pH decreases towards the left of the plot, protonation of the cysteines occurs and causes free metal ions to be released. This happens sooner, i.e. at higher pH, for the more weakly binding Co^2+^ ion than it does for Zn^2+^. In the case of zinc binding, an intermediate complex MH_3_L^+^ appears at moderately low pH as a result of protonation of both nitrogens on both histidines, which apparently is still compatible with metal binding; in contrast, in the case of cobalt binding, Co^2+^ release is essentially complete at pH values where the histidines become protonated, so the corresponding intermediate complex is barely populated. At high pH values, deprotonation of the lysine and binding of an OH^-^ ion occur, but these high pH values are unlikely to be relevant for most NMR studies. It seems reasonable to suppose that very similar principles would apply more generally in other cases, except that a) usually there would be many other ionisable side-chains that would contribute to the overall profile, and b) in cases where only cysteines are involved in the zinc binding, there can presumably be no “intermediate” complex analogous to MH3L^+^ (where histidines but not cysteines are protonated). Also, just as the pH range over which the cysteine protonation transition occurs differs between the zinc and cobalt complexes in the case shown here due to the difference in binding affinities, it seems reasonable to suppose that it would also vary as a function of differences in zinc-binding affinity amongst different zinc fingers, with metal dissociation occurring at higher pH values (i.e. more readily) for those proteins that bind zinc more weakly.

When selecting a suitable pH value for studying a zinc finger protein by solution NMR, the pH dependence of metal binding discussed above is one of three key considerations, the other two being avoiding a pH value that coincides too closely with the iso-electric point (pI) of the protein (since, as for any solution study of a protein, such a pH value is likely to lead to protein aggregation and/or precipitation), and avoiding too high an amide proton exchange rate with solvent. The importance of amide proton exchange rate arises because of its effect on detectability of amide proton signals from H_2_O solutions of proteins. This is critical for NOE-based determination of their structures by NMR, as numerous NOE interactions that are needed to characterise the structure involve these protons directly (indeed, detectability of amide NH protons is also critical for most other solution NMR studies, e.g. of interactions with other biological molecules). This in turn implies that high pH values are incompatible with NMR structure determination, since the faster solvent exchange of amide protons that occurs at high pH causes intensity losses and/or linebroadening that can render their ^1^H resonances completely undetectable. It follows that for a zinc finger protein, one must use a pH value that lies within a window between two approximate limits; it must be high enough to avoid metal dissociation, yet low enough to avoid excessive amide NH solvent exchange (while also avoiding the iso-electric point). For well-folded proteins with strongly-bound zincs, this window can be quite broad, maybe from around pH 5.5 to 8 or thereabouts, but for less stable proteins the window is likely to be narrower; values around 7 generally represent a good compromise and are very frequently used, but there are no guarantees of what will work. Reducing the temperature may be helpful in difficult cases, but the benefits in terms of solvent exchange rate and sample lifetime must be balanced against the strong probability that signals will be broader at lower temperatures, leading to reduced sensitivity. It is also worth bearing in mind that there is always a spread of values for amide NH solvent exchange rates, so the signal loss is not uniform but rather affects an increasing proportion of the faster-exchanging amide signals as pH is increased; in general, loss of a small proportion of amides at some moderately high pH may well be preferrable to the faster sample degradation that occurs at lower pH.

### 3.3 Oxidation

Another important factor that can limit long-term stability is oxidation. Slow oxidation of zinc finger proteins in solution by dissolved oxygen on a timescale of days has been reported [100], leading to products in which the metal has been released and disulfide linkages formed amongst some or all of the cysteines. In at least one early study of a TFIIIA-type C_2_H_2_ zinc finger (finger 2 from the yeast transcription factor SWI5), it was reported that sample degradation led to predominantly unfolded material in which the two metal-binding cysteines had probably become disulfide linked [11]. This author can also report that in some early studies, before additional precautions were taken to limit oxidation, concentrated zinc finger samples were seen to form a solid precipitate at the meniscus in the NMR tube over a period of weeks, presumably indicative of slow reaction with oxygen.

It is common practice to include a reducing compound such as DTT, TCEP or (less often) BME in the NMR buffer in order to limit, or at least slow down, oxidation. Mostly, the pH of NMR samples is sufficiently low that the redox reaction usually associated with the reducing action of DTT or BME, i.e. thiol/disulfide exchange, would be expected to be ineffective as the thiol groups would not be deprotonated. However, it seems likely that the mechanism of any protection offered by these reducing agents may be different for zinc finger proteins, in that the agents may be acting as scavengers of oxygen-containing free radicals; this has been reported at least in the case of DTT [101]. Another precaution sometimes taken is to replace the air above the sample in the NMR tube with nitrogen or argon, the latter sometimes being preferred due to its higher density in the hope that it would slow diffusion of any residual oxygen that may still be present (though in fact gaseous diffusion rates vary surprisingly little with the composition of a mixture, and are typically fast enough that complete mixing in a space as small as the top of an NMR tube would be expected to be complete within minutes, even in the absence of convection [102]). Subsequently flame-sealing the NMR tube using a dedicated gas-line then gives the longest-lasting protection, but is obviously less convenient than using a removable miniature suba-seal cap, particularly if access to the sample is still needed for any reason.

### 3.4 Choice of buffer

Given these various factors that can cause instability, it is not surprising that concentrated samples of zinc fingers for NMR not infrequently develop precipitates over time, and a key consideration in selecting buffer components is to minimise the chance of this happening (even though a rational approach can be difficult, as it is rarely if ever known what the precipitates actually are!). As with any solution protein study, ionic strength is a crucial parameter, since an inappropriate choice is likely to lead to protein aggregation and/or precipitation; highly charged proteins are generally more soluble at high ionic strengths whereas for hydrophobic proteins the reverse is true, but experimentation is the only truly reliable guide in specific cases. Turning to other buffer components, at least one specific protocol has been published that combines a number of relatively safe choices for zinc finger NMR studies [103], while at the same time something of a “folklore” has grown up surrounding components that might potentially create problems. For instance, zinc phosphate is extremely insoluble [104], and it might be supposed that using phosphate buffer might result in slow removal of zinc from the protein to form a zinc phos-phate precipitate; at the least, one might expect such a precipitate to form if excess of a soluble zinc salt is present. At least one early study employed pyrophosphate buffer to avoid this potential issue [6,9,11], since zinc pyrophosphate is less insoluble than the phosphate (depending also on the pH [104]), although it is slowly hydrolysed to phosphate over time. Tris buffer soon became estab-lished as by far the most popular choice, even though the pH of most NMR samples lies close to the edge of, or below, the optimal buffering range of Tris (approx. pH 7–9). In addition to the empirical widely made observation that solutions of zinc fingers in Tris buffer are usually well-behaved, it has the advantages that its ^1^H NMR spectrum comprises only a single line,it can be easily and rel-atively inexpensively bought in deuterated form, and being based on a large and only singly charged cation it has a relatively low electrophoretic mobility, which is important in maximising sensi-tivity of measurements in high-Q probes such as cryogenically cooled types [105]. That said, not all properties of Tris are desirable; its pK_a_ varies relatively strongly with temperature (by approx. –0.03 pK_A_ units per °C), and its stabilising influence could potentially be due to interactions dissimilar to any the protein would have in its native environment [106]. Although not specifically concerned with NMR measurements, a useful and highly relevant discussion of the biological inorganic chemistry of zinc, covering also the compatibility with Zn^2+^ ions of many buffers and reagents used *in vitro*, has been given by Krężel[107].

In an effort to cut through some of this potential uncertainty, the author undertook a survey of the BMRB database to determine what choices have actually been made for solution NMR studies of zinc fingers in practice, selecting for analysis any proteins that contained at least one zinc or cadmium ion bound to at least two cysteine residues. As at November 2021, 734 protein entries were found in the BMRB for which the protein contained zinc or cadmium, of which 501 match the further criterion of binding to at least two cysteines.^1^ Of these, Tris buffer was used in 310 cases, phosphate buffer in 81 (of which 10 included a small excess of ZnCl_2_ or ZnSO_4_, generally either 0.1 mM or 0.01 mM), while a further 72 were unbuffered (not counting NaCl as a buffer), and a handful of other buffers (HEPES, MES, acetate, MOPS, PIPES and pyrophosphate, mostly undeuterated) made up the remainder. Other common additives, other than NaCl and KCl, included DTT (314 cases), BME (14 cases), TCEP (52 cases) and sodium azide (used as a biocide, 233 cases). Of course, these data do not reveal how satisfactory the long-term stability of each of these samples actually was in practice, but it must have been sufficient for the data reported to have been acquired. Maybe the overall message should be that, although samples of zinc fingers do require particular care during preparation, handling and use, they are perhaps usually more robust than sceptical NMR spectroscopists sometimes give them credit for.

### 3.5 Domain boundaries

In some cases, zinc finger domains occur in the context of very small proteins where the full-length protein is not much, if at all, larger than the ordered zinc finger domain. This is for instance true of Rds3, where the full-length protein has only very short, disordered tails extending beyond the ordered domain, and in the case of Bud31, where there is an N-terminal helical subdomain and a short C-terminal tail but the full-length protein is still only 157 residues. In probably the majority of cases, however, zinc finger domains occur in the context of much larger proteins that may (or may not) contain multiple fingers as well as other unrelated functional domains and/or substantial disordered regions. In undertaking structural work in such cases it is usually necessary to study fragments “excised” from the full-length sequence, to limit the complexity of the system and, at least in the case of NMR, to bring the protein within the technical limits of applicability of the technique. A key question is, then, “how should the sequence for study be designed?”.

This is of course a topic of importance not just for NMR studies of zinc fingers, and I will not attempt to review the topic of bioin-formatic identification of domains (for one recent review, see [108]). However, usually the problem when designing an NMR study is slightly different, in that one may already know the approximate location and boundaries of the domain, but the question remains exactly where is the best place to “cut” the full-length sequence, assuming that the fragment will be obtained either by cellular over-expression or chemical synthesis and thus its N- and C- termini may be freely chosen. Of course, the most important consideration is to avoid cutting within the structured domain, which inevitably means that a few disordered residues will be retained at each terminus. An extreme example of what can happen if fragments are chosen that unintentionally interrupt the natural domain structure is provided by work on domains from the CREB binding protein, where a short fragment that was studied by one group was found to adopt stable structure with no known structural homology to other zinc fingers [109,110] that was sub-sequently shown to be completely different from that of a longer fragment including the whole of a very closely related domain [111]; while the observation of the structure of the shorter form was of theoretical interest, it was presumably not relevant to the biological function of the natural protein. Programs such as PONDR [112] that predict disorder in a given sequence can be useful in refining the definition of the domain boundaries, but probably the most generally reliable approach is to prepare actual samples of a small selection of different length fragments and test them experimentally, including by NMR. Although assignments would presumably not be conveniently available at such an early stage in a project, the distinctively narrow lines and random coil chemical shifts of disordered residues are likely to allow relatively easy identification of their signals and in some cases assignment to specific amino-acid spin-system types, so that the exact lengths of the disordered tails may be determined even without any large-scale assignment work. Another advantage of such tests is that fragments with tails of slightly different lengths may well differ in their solution properties (e.g. if a change in overall charge brings the isoelectric point closer to, or further from, the pH of the solution) or in their expression levels, so that testing a small panel of candidates allows an optimum fragment length to be chosen. Time spent on such optimisation early on is likely to be rewarded later by clearer results and better-behaved samples.

One other point concerning sequence design concerns the use of nickel-affinity His tags, which are very widely used throughout structural biology to facilitate purification of over-expressed proteins. Some workers, this author included, have avoided inclusion of His tags in sequences of zinc fingers for NMR on the precautionary principle that a tag designed to work by tightly binding a small, divalent transition metal might affect zinc-binding properties. However, there are many examples of zinc fingers where a His tag was used and remained in place during NMR structure determination (see e.g. [113–119]), so it seems that view may be over-cautious.

## 4 Calculating structures

There is quite a wide variety of methods that are used to calculate protein structures from predominantly NOE-based solution NMR data, and it would be beyond the scope of this review to attempt to describe them in any depth. However, in each case there are a number of specific issues that are quite particular to zinc finger proteins, and it is these that will be discussed in this section.

Purely for the purposes of this section it will be assumed that it is already known which residues, and which atoms in those residues, actually co-ordinate the zinc. This is of course absolutely crucial information that in large part defines the outcome of the structure determination, and methods for obtaining it will be covered in depth in Section 5. The main reason for structuring the discussion in this way is that sometimes identification of the zinc-binding ligands, or of the binding topology (i.e. the arrangement of which ligands bind which zinc in cases where more than one zinc is bound), is intimately coupled with the structure calculation, so it is convenient to have dealt with the nature of the calculations themselves before tackling the wider questions of ligand identification and metal-binding topology.

### 4.1 Forming the metal-binding site(s)

**Figure 7:**
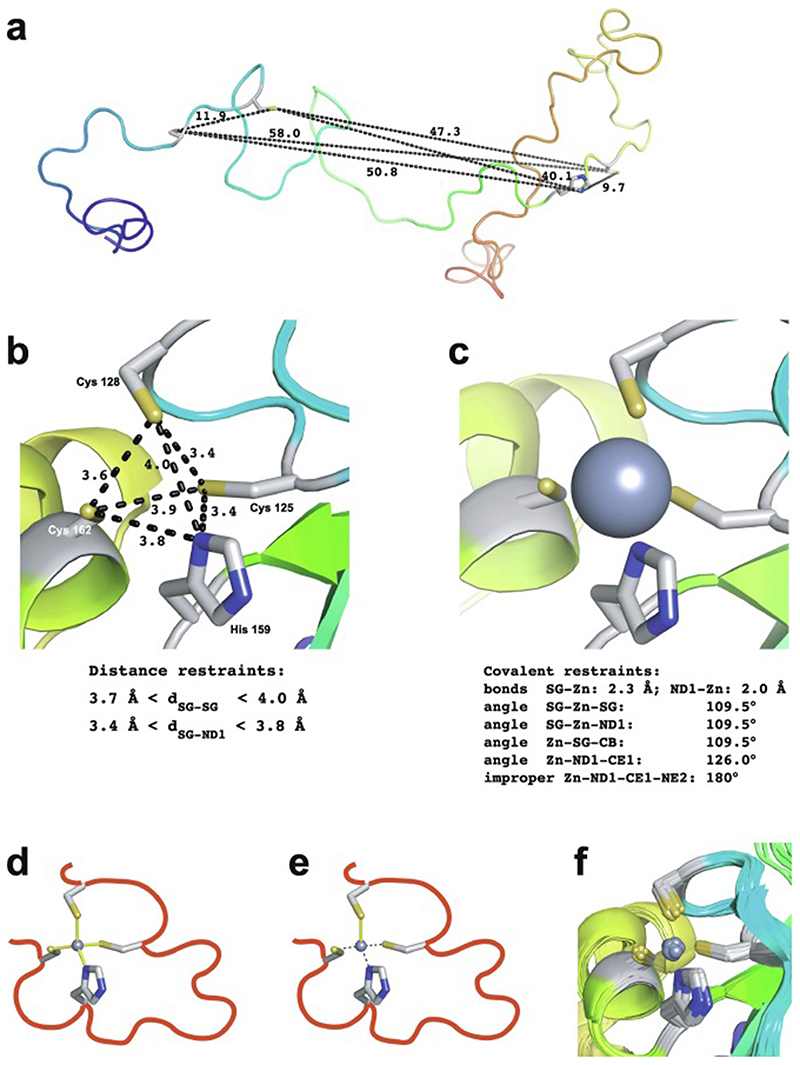
Incorporation of zinc ions during structure calculations of zinc fingers. The example shown is of the second zinc finger (residues 109–200)of human PARP-1 [120](PDB 2l31),calculated with the program XPLOR-NIH [121].The atoms that bind the single zinc in this case are Cys125S^γ^,Cys128S^γ^,His159N^δ1^and Cys162S^γ^,and all distances shown are in Å. **a**) Backbone cartoon of one randomised starting structure, showing initial values of the inter-ligand distances. **b**) Structure after first stage of calculation protocol. Only inter-ligand restraints (black dashed lines) have been applied to supplement the NMR-based experimental restraints at this stage and no zinc ion has been included in the calculation. Since these restraints act by adding an energy penalty if the relevant distances violate the restraints (rather than by imposing an absolute limit), some distances can lie slightly outside the restraint ranges. **c**) A zinc ion is introduced at the geometric average of the four ligand atom positions with covalent binding restraints as listed (the improper restraint acts to keep the zinc in the same plane as the histidine aromatic ring), and then further molecular dynamics acts to relax the structure in the modified force field. **d**), **e**) Steps needed to calculate a structure including explicit zinc using torsion angle dynamics. Panel d) shows how connection of the zinc ligands through the protein backbone (schematically shown in red; zinc ion shown at 30 % size for clarity) forms rings within the overall structure of the protein, the presence of which is incompatible with torsion angle dynamics calculations; panel e) shows how this issue is overcome, by “cutting” three of the four bonds to zinc, and replacing their restraining effect on the structure by using distance restraints (black dashed lines). See text for further discussion. **f**) Ensemble view of the same calculation shown in c). All cartoons were produced using the program PyMol [23,24] and are shown in “chainbow” colouring (blue → red, N-terminal → C-terminal) while zinc ions are shown as grey spheres.

Essentially all methods for calculating protein structures from NMR data use multiple, repeated instances of the calculation that each start from a randomly different starting state. In the ensemble of final structures that results, regions that have converged to a consistent conformation can thus be distinguished from poorly converged regions where the data are insufficient to define the structure to high resolution. Calculations based mainly on NOE-derived distance restraints generally use starting structures with randomised backbone conformations, and this immediately raises a problem for calculations with zinc finger proteins since the zinc-binding ligands will, in all probability, be far apart in space in such starting structures (Fig. 7a). For any calculation that uses a force field, defining zinc-ligand bonds in these structures would then lead to impossibly high energy terms that would immediately cause the calculation to fail.

The obvious solution to this problem is to divide the calculation into two parts. In the first, no zinc ion is defined and the structural restrictions resulting from zinc-binding in the immediate area of the binding site are instead represented approximately by imposing ligand–ligand distance restraints in much the same way as if they were NOE-based restraints. The resulting structures, though perhaps of limited resolution or accuracy around the binding site, should bring the four ligand atoms of each zinc into at least roughly the correct locations (Fig. 7b), and these structures can then be used as the starting point for the second stage of calculation in which an explicit zinc ion is introduced at the position of the geometric average of the ligand atoms. During this second stage, the zinc-ligand bonding and geometric terms in the force field, in conjunction with all the terms representing the rest of the structure, enforce the detailed structure of the binding site (Fig. 7c). This approach has been widely followed, although often details of implementation are only very briefly mentioned.

Of course, the major task of forming the overall architecture of the binding site, in conjunction with the rest of the protein structure, falls to the bulk of the NMR-based restraints, most particularly the NOE-derived distance restraints. If these form a sufficiently dense and self-consistent network, this will lead to a well-defined and converged structural ensemble, and addition of restraints specifically concerned with ligand–ligand atom dis-tances and/or zinc-binding geometry may make only relatively minor local differences (this is for instance the case in the example of Fig. 7). However, if the NMR data are more sparse, then the work done by the zinc binding restraints in imposing the structure may become more critical; such structures are probably of lower resolution, but would be of much lower resolution still were it not for the zinc restraints. Of course, in any NMR structure determination of a zinc finger, if the ligand atoms or the binding topology have been wrongly identified this may well lead to major conflicts between the inter-ligand restraints and the NOE-based experimental restraints, depending on the degree of structural adjustment that would be necessary to fulfil the incorrect inter-ligand restraints (and on the quality of the NMR restraints), and this could provide an important clue to identifying such problems.

It is important to note in this context that 𝓧^1^ torsion angle restraints for the sidechains of the metal-binding residues assume a particular significance in determining zinc finger structures, well beyond their importance for other residues. Effectively they have the same significance as backbone torsion angle restraints, and it is generally worth investing time in measuring data that can define such restraints, at least at the level of establishing which rotameris populated for each of the metal-binding sidechains. This usually has the added benefit of simultaneously generating stereoassignments for the H^β^ protons of these and some other sidechains, which helps improve the resolution of the final structure [122]. One useful way to obtain such restraints is by analysing the results of a pair of HNHB [123] and HACAHB [124] spectra.

A somewhat different situation to that described above applies in the case of distance-geometry calculations (see e.g. [125] for a description of this method), but although this approach was quite widely used in the early days of protein structure determination by NMR (including for the very first zinc finger structure, that of Xfin 31 [3], which used the program DISGEO [126] for the first stage of calculation), it has since fallen essentially completely out of use.

### 4.2 Torsion angle dynamics and ring closure

Many methods for calculating structures using NMR-based restraints employ so-called “torsion-angle dynamics” during the protocol. In such calculations, motions within the structure are no longer calculated independently for individual atoms or groups using their three-dimensional (i.e. Cartesian) co-ordinates, but rather using a hierarchical scheme based on torsion angles about the rotatable bonds within the structures as the effective variables [127–129]. The Cartesian co-ordinates are still used to calculate the energies at each time step, meaning that distance terms in the force field such as NOE-based restraints can still be used to influence the calculation, just as in purely Cartesian-space calculations. This implies that there is frequent interconversion between Cartesian and torsion-based representations of the structure, but the much greater speed of calculations of velocities and accelerations in torsion space still makes the torsion dynamics calculations much more efficient than purely Cartesian-space calculations, and this is the main motivation for using them. However, a significant limitation of torsion angle dynamics calculations is that they cannot straightforwardly describe structures containing rings. The method adopted to deal with such situations is to “cut” one bond in the ring during those stages where the calculation is working in torsion angle space, using a distance restraint to maintain the close proximity of the previously bonded atoms, and then to restore the bond once the calculation returns to Cartesian space; this approach was first developed to deal with sugar rings in RNA [129], but the same principle applies, for instance, to disulfide bonds in proteins.

In the case of zinc finger proteins, every zinc has four bonds to protein sidechains, each of which closes a ring (Fig. 7d), so it is necessary to “cut” three of them during torsion dynamics stages (Fig. 7e). This is generally not something that users would have to implement themselves, and for instance at the time of writing a working solution with files and instructions is available online for use with ARIA 2 [130], which is one of the most commonly used programs for NMR structure determination that employs torsion dynamics (see https://aria.pasteur.fr/documentation/tutorials/how-to-run-aria-with-a-zinc-ion-bound-to-cys-and-his). In some other cases, for instance sometimes when using CYANA [131],no zinc ion is defined during the torsion angle dynamics stages, instead using inter-ligand distance restraints to approximate the structural consequences of zinc binding just as for the first stage calculations described in Section 4.1. Sometimes such structures are directly deposited without including co-ordinates for zinc (such cases were of course not identified in the author’s search of the BMRB database, precisely because they lack zinc, but one such example is PDB 2jsp [132]). More commonly, an explicit zinc ion is introduced at a later stage during refinement in Cartesian space, generally using a different program such as AMBER [133].

Similar considerations to those just discussed would apply to other calculations that work mainly or entirely in torsion angle space, such as the early program DISMAN [134], but rather like distance geometry methods this is rarely if ever used any more.

### 4.3 Detailed geometry around zinc

As pointed out in Section 1, the zinc in zinc fingers is generally NMR silent. In the absence of direct information available from NMR data, the detailed nature of the zinc-binding geometry (and indeed even the presence of zinc in the structures at all) is therefore based on other external information and is largely or entirely imposed by the force field during the structure calculation. The parametrization of the force field in turn reflects expectations for the nature of zinc binding as seen in protein crystal structures and, at an earlier stage before crystal structures were available for zinc fingers, EXAFS data specifying bond lengths [74,76]. Different methods and software packages specify the zincbinding geometry to widely differing levels of detail. As previously noted, some calculations simply omit the zinc ion altogether, relying on inter-ligand distance restraints to reproduce the arrangement of ligands at the binding site at least approximately. Sometimes inter-ligand restraints are used together with an explicit zinc ion and zinc-ligand distance restraints so as to reproduce the expected bond lengths (approx. 2.3 Å for Zn-S and approx. 2.0 Å for Zn-N) (see e.g. [90]). More commonly, a more complete set of covalent geometry restraints is employed, such as those shown in Fig. 7b which were used for calculations in XPLOR-NIH [121]in the author’s group [120]. Typically, in addition to bond lengths, these will include a number of bond angles set to the value for tetrahedral co-ordination (109.5°), as well as parameters to keep the zinc in plane and appropriately oriented with respect to any histidine rings to which it is co-ordinated (i.e. approximately aligned with the expected direction of the appropriate nitrogen sp^2^ lone-pair orbital). A particularly widely used program for refinement is AMBER [133], which is very powerful not just for structure determination but also in many other areas where molecular dynamics simulations are applied, and which offers different possibilities for parametrizing zinc bound to proteins (see e.g. [135,136]); as generally used for NMR structure refinement the parameters are sim-ilar to those in Fig.7b but may also require inclusion of a number of dihedral angles around zinc.

It is quite difficult to form an accurate overall picture of program usage and outcomes across the field, particularly since two or more programs are often used in sequence in the same study. For instance, programs such as CYANA [131], UNIO [137], XPLOR-NIH [121]or ARIA [130]may be used first for iterative, automatic or semi-automatic NOE cross-peak assignment that does most of the “heavy lifting” of defining the overall protein structure but may or may not include treatment of the zinc, after which a second program such as AMBER [133] may be used for refinement, including explicit or implicit representation of solvent and explicit representation of the zinc. But it may also be that these tasks are handled in separate stages within a single program package, or additional steps (such as manual refinement of the NOE restraint list) may be added, and without a detailed reading of every relevant “materials and methods” section it is not necessarily clear whether every program used is listed in every deposition. Nonetheless, some generalities are clear. Of the 501 BMRB entries identified in November 2021 as containing one or more zinc or cadmium ions bound by at least two cysteines (see Section 3.4), 269 are associated with a deposited co-ordinate set in the PDB database. Of these, a high proportion (approximately 160) were determined by the Riken Structural Genomics Initiative (RSGI [138]), and these used a combination of the programs KUJIRA [139] for NOE cross-peak assignment and CYANA for structure calculation and refinement. Unfortunately, details of how the zinc was handled in these cases are not at all easily to find, since there are no detailed publications or descriptions associated with these depositions. It seems likely (to this author) that appropriate parameters for zinc may have been added to the CYANA force-field in much the same way as for other dynamics programs, but alternatively it could be that the zinc was positioned simply using distance restraints. However, in cases where CYANA (or UNIO, which most frequently uses CYANA for its molecular dynamics calculations) or its predecessor DYANA [129] was used by other groups and where publications are available, it seems that it is common practice to omit the zinc during the CYANA stage and then use a second program, commonly AMBER, to introduce the zinc and refine the structure. When XPLOR [140], CNS [141] or XPLOR-NIH [121] is used for the first (zinc-free) stage of calculation, the zinc may be added and refined within the same package or by using a second package such as AMBER, while some other calculations use AMBER throughout.

Given that the exact geometrical nature of the bonding arrangement around zinc in NMR structures of zinc fingers depends so heavily on generalised data that do not themselves come from the protein under study, it follows that analysis of any specific deviations from regularity in individual NMR structures is unlikely to be justified. As noted earlier, formation of the ligand-zinc bonds closes rings within the structure, constituting a very powerful structural constraint. If there are any errors elsewhere, such as sidechains in the wrong rotamer, local distortions of the peptide backbone, or minor errors in the NMR-based restraints such as incorrect distance calibration of individual NOE restraints, such errors will in general be more difficult to accommodate in the context of a closed ring. The way in which such issues will reach a balance with the constraints of covalent bonding will then depend strongly on the relative values used for different force constants. While there have been some detailed studies using quantum calculations to improve the parametrization of zinc in molecular dynamics calculations for refining X-ray structures of zinc proteins (e.g.[142]), there is little or nothing to go on when attempting to set “correct” values of force constants for zinc parameters for use in NMR structure calculations, and it seems likely they are often set according to personal opinions of individual authors (i.e. they are either set the same as for other bonds within the structure, or deliberately made somewhat weaker). Overall, it seems likely that if there are problems elsewhere in an NMR structure of a zinc finger, their influence may accumulate in distortions around the zinc(s), and such distortions may have little relation to the (essentially unknowable) reality. On the other hand, it should also not be a surprise to see very regular and well-defined geometries of zinc-binding sites in NMR structures of zinc fingers where problems such as those mentioned above are largely absent, as this simply reflects the regularity specified in the force field (see e.g. Fig. 7f).

Notwithstanding the above, at least one study has combined NMR structure determination with EXAFS data for the same protein and reported specific deviations from regular geometry around zinc, in this case for histidine in the C-terminal zinc- binding domain of SecA ATPase [97], co-ordinates for which were deposited for calculations carried out both with (PDB 1sx1) and without (PDB 1sx0) the zinc. This particular case is unusual in that two of the zinc ligands, the histidine and one of the cysteines, are immediately adjacent in the primary sequence, which unfavourable arrangement probably largely accounts for the distortions. More recently, a useful web resource called “CheckMyMetal” (CMM, https://cmm.minorlab.org) has been introduced that can validate a wide range of metal-binding sites in proteins, including zinc sites in zinc fingers; the original publication cites examples of both “good” and “bad” zinc sites in NMR structures [143].

### 4.4 Zinc binding chirality

**Figure 8:**
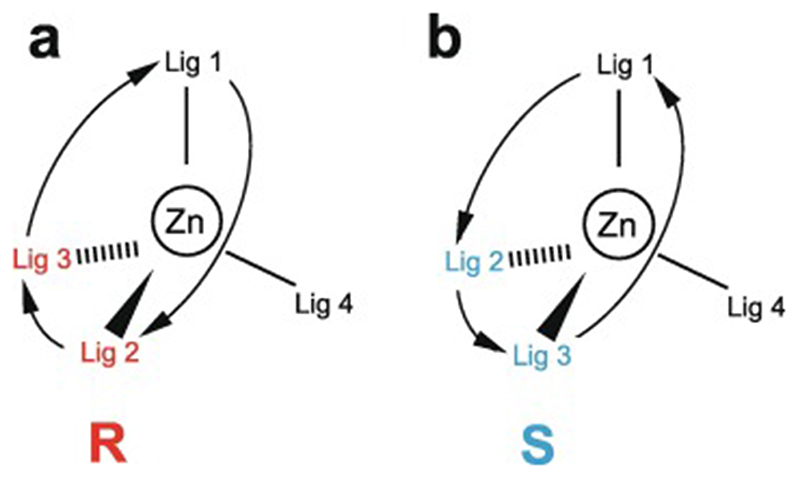
Chirality of zinc-binding sites. Just as with tetrahedral carbon, tetrahedral zinc sites are chiral and can therefore exist in two enantiomeric forms. Following the convention defined by Berg[2], the ligands are assigned a priority order running from N-terminal (highest priority, “Lig 1” in the figure) to C-terminal (lowest priority, “Lig 4” in the figure). The chirality is then defined as R (for rectus) ifligands 1, 2 and 3 increase in a clockwise sense when viewed along the bond from Zn towards ligand 4, as shown in **a**). The opposite arrangement, shown in **b**), is defined as S (for sinister) and can be reached from Rbyswapping anytwo ofthe ligands, as shown here for swapping 2 and 3. As a real example, for the site shown in Fig. 7b the ligands 1, 2, 3 and 4 correspond to respectively Cys125, Cys128, His159 and Cys162, and the site has an S configuration.

If one considers aspects of the structure a little further removed from the zinc itself, a very useful criterion is to check the chirality of each zinc-binding site, a concept originally introduced by Berg [2]. Just as for a tetrahedral carbon atom bearing four different substituents, the four ligands around a tetrahedral zinc form a chiral set with two possible enantiomeric arrangements being possible (see Fig. 8a and b), and because the zinc is embedded in an intrinsically chiral environment (the protein structure), these two arrangements correspond to different stereoisomers.

The priority ordering of the ligands needed in order to define which arrangement is R and which is S is simply based on their sequence position, running from N-terminus (highest priority) to C-terminus (lowest priority); this rule fulfils essentially the same function as the “Cahn-Ingold-Prelog” convention[144,145] used to prioritise the four substituents on a chiral carbon atom. In practice, identifying whether the arrangement around a particular zinc is R or S is made much easier by using a general computational approach to assigning chirality published by Cieplak and Wisniewski in 2001 [146], which can be straightforwardly scripted to work with PDB files. The key equations are given below:

$$\begin{array}{l} \text{Determinant}\;(1)=((B_{x}-D_{x})(C_{y}-D_{y}))-((B_{y}-D_{y})(C_{x}-D_{x}))\\ \text{Determinant}\;(2)=((A_{x}-D_{x})(C_{y}-D_{y}))-((A_{y}-D_{y})(C_{x}-D_{x}))\\ \text{Determinant}\;(3)=((A_{x}-D_{x})(B_{y}-D_{y}))-((A_{y}-D_{y})(B_{x}-D_{x}))\\ \text{Determinant}\;(4)=((A_{x}-C_{x})(B_{y}-C_{y}))-((A_{y}-C_{y})(B_{x}-C_{x}))\\ \text{Result}\;=A_{z}\text{Determinant}\;(1)\\ -B_{z}\text{Determinant}\;(2)\\ +C_{z}\text{Determinant}\;(3)\\ -D_{z}\text{Determinant}\;(4)\\ \text{If}\;\text{Result}\;>0\text{arrangement}\;\text{has}\;\text{chirality}\;S\\ \text{If}\;\text{Result}\;<0\text{arrangement}\;\text{has}\;\text{chirality}\;R\\ \text{If}\;\text{Result}\;=0\text{arrangement}\;\text{is}\;\text{achiral}\; \end{array}$$

where A_x,y,z_, B_x,y,z_, C_x,y,z_ and D_x,y,z_ represent the Cartesian co-ordinates of the four ligating atoms in decreasing priority order (i.e. N-terminal to C-terminal).

There are no specific rules as to which chirality any given zinc site should have, though the fact that TFIIIA-type fingers (and some other common types, such as GATA) are all S ensures that S chirality is the more common. However, determining the chirality can provide useful tests. For instance,if a new structure is to be consid-ered homologous to an existing structure, clearly their chiralities should be the same. Another point is that one hallmark of a well-determined NMR zinc finger structure is that, for each zinc site, all the ensemble members should have the same zinc chirality. This might seem obvious, but if any of the ligating residues are located in even slightly poorly defined regions of the structure it can be surprisingly easy for inconsistent chiralities to be present in a calculated ensemble; sometimes only quite small structural shifts are needed to interconvert the enantiomeric forms. One might perhaps wonder if this (sometimes) represents genuine mobility. The author knows of no study that addresses this point specifically, but subjective experience strongly suggests such cases instead reflect poor restraint density. It is worth noting that usually the most powerful restraints that act to define the chirality uniquely are 𝓧^1^ torsion angle restraints for the ligating residues, the importance of which was already highlighted in Section 4.1.

### 4.5 Interactions between finger domains

**Figure 9:**
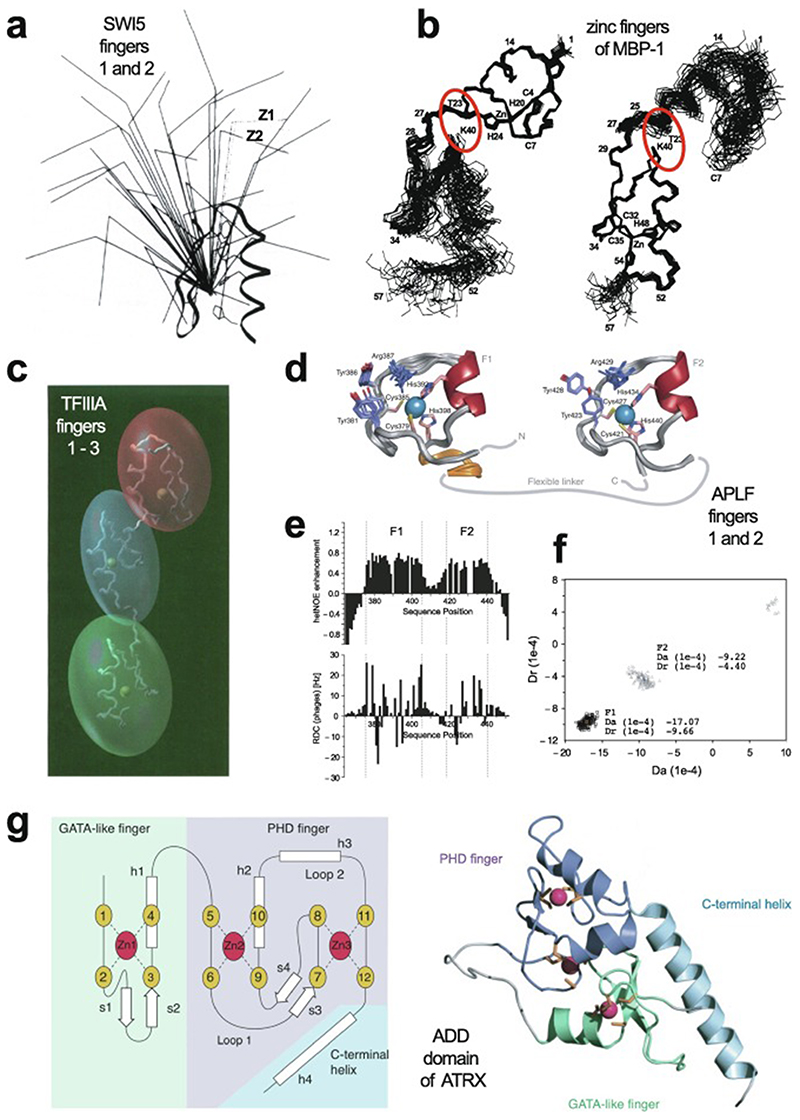
Inter-finger interactions as seen by NMR. **a**) Schematic representation of the distribution of inter-finger orientations within a calculated ensemble of the first two TFIIIA-type zinc fingers in the transcription factor SWI5, showing the steric restriction on the available conformation space sampled by finger 1 when finger 2 is fixed. No inter-finger restraints were applied during these calculations, as no inter-finger NOEs were assigned in the corresponding NOESY spectra. Each ofthe 30 two-finger structures is represented by two lines, one joining the two zinc ions and the other joining the zinc ion of finger 1 to the tip of finger 1 (Arg 25 C^α^). The structures are superposed on the lowest energy structure of finger 2 (using N, C′ and C^´^ofresidues 42 –66). The dotted lines labelled Z1 and Z2 represent the mutual orientations seen in the crystal structure of Zif268 bound to DNA (Z1 shows the position of Zif268F1 when Zif268 finger 2 is superposed on SWI5 finger2, and Z2 shows the position of Zif268 F2 when Zif268 finger3 is superposed on SWI5 finger 2) Adapted with permission from[11] (©1992 Elsevier). **b**) Structural ensemble of the two TFIIIA-type zinc fingers from the protein MBP-1, superposed on either the N-terminal finger (left; residues 2–28) or the C-terminal finger (right; residues 27–55). In this case, the relative orientations ofthe two fingers were largely, but not completely, constrained by a cluster of 14 long-range NOEs (|i-j| > 4) in roughly the position marked by the red ellipse, linking residues Thr23 or Val27 on finger 1 with residues Phe39, Lys40 or Thr41 on finger 2. Adapted with permission from [7](©1992 American Chemical Society). **c**) Model for the averaged overall alignment of the first three zinc finger domains in TFIIIA itself, derived by analysis of anisotropic rotational tumbling effects present in ^15^N relaxation data for this system. The ellipsoids (red for F1, blue for F2 and green for F3) superposed on the backbone structures of the fingers represent the rotational diffusion tensors of each finger (see text for further explanation), and characterise their differential mobilities. Overall, the molecule is highly elongated. The ellipsoid on the central finger is the smallest and most anisotropic, reflecting its relatively slow and restricted rotational tumbling, while the largest ellipsoid is that on F3, which has the greatest motional freedom. Adapted with permission from [12] (© 1995 The American Association for the Advancement of Science). **d**)Structural ensemble (25 lowest energy structures shown from 50 total), **e**) ^15^N relaxation and RDC data, and **f**) fitted alignment tensors for a fragment containing both of the PAR-binding zinc finger domains from the protein APLF ordered using Pf1 phage (12 mg/ml). These data show that the two domains do not mutually interact; the axial (Da) and rhombic (Dr) components of the fitted alignment tensors shown in f) show well-defined clusters for each finger that are very distinct from one another (see text for further discussion). Finger 1 is more strongly ordered than is finger 2, as shown both by the larger spread of measured RDC values for finger 1 and correspondingly by the larger magnitudes of the alignment tensor. The ^15^N{^1^H} heteronuclear NOE data show that the linker between the fingers is highly mobile. Panels d, e and f adapted with permission from [58] (© 2010 Eustermann et al.). g) Structure of the ADD domain of ATRX protein, which is an example of a single domain containing two zinc fingers of different types fused together. Adapted with permission from [157](© 2007 National Academy of Sciences).

As discussed in Section 1, many proteins contain multiple individual zinc finger domains connected by linkers, or in some cases separated by other intervening domains. This is particularly true for the best-known class of zinc fingers, the TFIIIA-type of C_2_H_2_ fingers, where sometimes many fingers may be present within a sin-gle chain(even as many as 37 in the case of Xfin),but other classes of finger domain can also frequently occur in twos or threes within the same protein. The bulk of this review is concerned with NMR structure determination of the individual domains, but it is also of interest to know whether there are defined structural relation-ships *between* domains, and if so how NMR can help to characterise these. In the majority ofcases the linkers between domains are largely disordered and flexible; this is clear either from predictions using programs such as PONDR [112]or in some cases from ^15^N relaxation data (see for instance Fig. 9e and refs. [10,147,148]). Thus, the issue of whether there are defined inter-finger structural relationships mainly comes down to the question, “do the individual zinc finger domains mutually interact?”.

Of course, the situation is likely to be completely different between the free and bound states of the protein. Again as discussed in Section 1, binding of a multiple zinc finger protein to its cognate partner will frequently impose order on the relative arrangement of at least some of the domains. For example, in the case of the DNA complex of Zif268 (Fig.1e) crystallography showed that adjacent C_2_H_2_ fingers follow the track of the DNA major groove [13], while in the more complicated case of the DNA complex of fingers 1–6 of TFIIIA different fingers bind in different ways with some jumping the DNA minor groove [149]; many other cases are known for different complexes of multiple zinc finger proteins with their various binding partners. Such changes in the degree of order upon binding are of course of absolutely crucial importance to understanding function, and in some cases analysis of complexes may suggest inter-finger interactions in the bound state, the status of which in the free protein is simply unknown (see e.g. [150]). However, such points are not the topic of this review; the purpose of this section is restricted to consideration of how and whether inter-finger interactions that occur within the free protein can be seen by NMR.

Just as with any other interdomain interactions within proteins, a full range of possibilities exists, depending on the extent and energetics of the interaction interface. These can range from strong interactions that effectively “glue” domains together so that they behave as a single structural unit with little or no remaining inter-domain flexibility, through weaker interactions that leave differing degrees of remaining flexibility between domains and may be intermittent in nature, right down to very weak, transient interactions involving very few contacts that merely bias, perhaps only slightly, the distribution of relative domain orientations in the overall conformational ensemble.

These different situations strongly influence the types of NMR data that can be used to characterise inter-finger interactions. The most direct evidence, when available, comes from detection of NOE connectivities seen between protons in different fingers. The simplest interpretations arise in cases where there is a reasonably extensive interface that largely quenches interdomain motions, as there are then likely to be a sufficient number of detectable NOEs to yield an interlocking mesh of restraints across theinterface,capable of defining the resulting arrangement with comparable accuracy and precision to those for the domains themselves. However, in cases where interfaces are smaller and weaker the number of detectable NOEs may be much smaller, and calculated structural ensembles may show only a partial restriction of the relative orientations of the finger domains. In the limit that there are only a very few detectable inter-finger NOEs, the outcomes of structure calculations may be expected to be quite strongly dependent on the parameterization of any corresponding distance restraints that are used, i.e. the exact distance ranges, functional forms and force constants chosen for the NOE energy terms in the structure calculations. A related complication for such weak inter-finger interactions is that conformations in which the interface is present may only be partially populated, attenuating the few inter-finger NOEs that exist to an unknown extent (relative to intra-finger NOEs) that would be difficult to reflect in the definition of distance restraints.

Two nearly simultaneous, early studies of fragments each containing two TFIIIA-type fingers serve to illustrate different situations that can occur in such cases. The yeast transcription factor SWI5 contains three fingers near its N-terminus, and comparisons of data from one- and two-finger fragments showed that in this case no interfinger NOEs were detectable, while chemical shifts of individual fingers were near-identical regardless of the presence or absence of adjacent fingers, strongly suggesting that the two domains, although tethered, are structurally independent[11]. This interpretation led to calculated structural ensembles (just the finger 1–2 case was calculated) in which the linker was completely flexible and the relative orientations of the domains were limited only by steric exclusion (Fig. 9a). In contrast, studies of a two-finger fragment from the human enhancer binding protein MBP-1 showed a cluster of NOEs linking the tip of the C-terminal finger to the base of the N-terminal finger; calculations that included distance restraints based on these NOEs led to structural ensembles in which the relative orientations of the fingers were largely, though not completely, restricted (Fig. 9b) [7]. Subsequently, examples of multiple finger fragments have appeared where interactions are present between some pairs of fingers but not others[151–153]; however, at least for TFIIIA-type fingers, it seems most commonly to be the case that inter-finger interactions in the free protein are minimal [154].Examples of interfinger interactions involving different classes of zinc finger domain have also been published, for instance in the RNA-binding proteins MBLN-1 [155] and Nab2 [156].

In cases where inter-finger interactions are minimal, a different approach has sometimes been taken to characterising average inter-domain orientations independently of NOEs, by interpreting detailed measurements of NMR relaxation parameters that reflect anisotropic tumbling. Simple treatments of relaxation treat the tum-bling of a molecule using a single correlation time, but for molecules that are sufficiently asymmetric, tumbling will occur at different rates about different axes; the fastest tumbling will be about the axis corresponding to the longest dimension of the molecule, while tumbling about shorter axes will be slower. If a sufficient quantity and quality of relaxation data can be measured and the molecular structure is known, then a detailed interpretation can yield the dimensions and orientation of the rotational diffusion tensor [158,159], which can then conveniently be displayed as a three-dimensional ellipsoid; the lengths of the ellipsoid along its three principal axes represent the different rotational diffusion coefficients for tumbling about the correspondingaxes of the molecule, and its orientation relative to the structure (if the structure is known) is defined by the data. This approach was first applied to a fragment of TFIIIA corresponding to the first three zinc finger domains [12]. In this study, ^15^N relaxation data were used and the analysis was carried out independently for each of the finger domains on the assumption that the structure of each finger remains relatively rigid, thus yielding a separate ellipsoid for each finger as shown in Fig. 9c. While this treatment does not necessarily yield a formally unique outcome (the orientations of the individual finger domain structures could be swapped through 180° about any axis, but possible arrangements are limited by the linked nature of the chain), the results show that the average structure is most likely to be a highly elongated arrangement, implying that motions of the individual domains are correlated on the timescale of overall molecular tumbling (i.e., < ~10 ns). Similar approaches have been used in studies of the six zinc fingers in the transcription factor MTF-1 [160], and to show that an interaction between fingers 3 and 4 in the thirteen finger transcriptional activator protein Miz-1 can be abolished by a mutation at the inter-finger interface [151]. It is worth adding here that long-range, short-timescale order of the type revealed by these relaxation mea-surements has little or no interpretable effect on other types of NMR data used in structure determination, so incases where no such analysis was carried out (as, for instance, in the case of SWI5 described above), such order could well have been present but gone undetected.

Residual dipolar couplings (RDCs) measured in a dilute nematic phase represent another type of NMR data intimately associated with molecular anisotropy [161,162], and they can be used as a valuable test for whether or not domains are rigidly constrained relative to one another [163]. Fitting measured RDC data for a known structure yields details of the particular strength and nature of the order imposed by the measurement medium employed (e.g. phages, lipid bicelles, etc.). In cases where two domains in a molecule are rigidly linked, such fitting should give essentially identical results when carried out over either of the two domains individually or over the two as a pair in a combined fit, but if the domains undergo relative motions then the alignment tensors found for each individual domain will be different (and an attempted combined fit would be poor). Importantly, this approach is sensitive to relative motions on essentially any timescale, irrespective of molecular tumbling rates in solution. Fig. 9d – 9f show an example of this method, demonstrating that the two PAR-binding zinc fingers of the protein APLF are mutually independent in the free state [58].

All the examples described above involve cases of adjacent fingers in a chain, but it is also possible for more than two domains to combine in a more intimate fashion to create a new, enlarged structure. Rds3 can be said to be an example of such a case, as the structure of its single domain is formed from three GATA-like fingers joined together, albeit in this case the individual fingers are still further constrained by the topological knot formed by the protein backbone [48]. Another example is provided by the ADD domain of the human chromatin associated protein ATRX [157], which is formed by fusion of a GATA-like finger, a PHD finger and a C-terminal α-helix to form a single enlarged structure (see Fig. 9g).

In closing this section, it is worth making a specific point about structural predictions made by the program alphafold2 [164].As dis-cussed in Section 6, this program represents a remarkably impressive breakthrough in the field of protein structure prediction using artificial intelligence, the significance of which to the whole field of structural biology, and indeed all biology, is immense. However, such predictions are not without limitations, and in the case of multiple zinc-finger proteins it is important to make a distinction between the prediction of structures of *individual domains* as opposed to the apparent prediction of structural relationships between domains. Some further discussion of the former is presented in Section 6; essentially, it is likely that predictions of structures of individual zinc finger domains are substantially correct in the majority of cases (except perhaps where both homology and secondary structure content are severely limited). However, the situation for relationships *between* fingers in multi-finger proteins is quite different, since very often in reality there may simply not be any defined, persistent, relative arrangement of the domains. In such cases, predicted structures can give a strongly misleading impression about the overall architecture and degree of compaction. For example, in a C_2_H_2_ TFIIA-type zinc finger protein chosen at random (Q96IT1), alphafold2 appears to show all of the zinc fingers clustered together in a relatively compact core, but there is essentially no reason to expect that this relationship exists in reality (which is why I have not included an illustrative figure at this point). The prediction results do perhaps give a clue to this issue that would likely be recognised by structuralists, in that the conformations of the linkers all have the lowest possible category of confidence in the prediction. However, the impression of a single overall structural arrangement that could result from a naïve interpretation in such a case should definitely not be taken at face value in the absence of concrete, experimental evidence that any predicted interfinger interactions are real.

## 5 Identifying ligands and metal-binding topology

As pointed out at the start of Section 4, the identification of the specific residues and atoms that co-ordinate each zinc in a zinc finger constitutes one of the most important and powerful inputs to an NMR structure determination of a zinc finger protein, and establishing these linkages and their topology correctly is absolutely essential to the outcome. Having earlier deferred the topic of how these are established, it will now be addressed in this section. It is assumed that the number of bound zincs has already been established (see Section 2.2).

**Figure 10:**
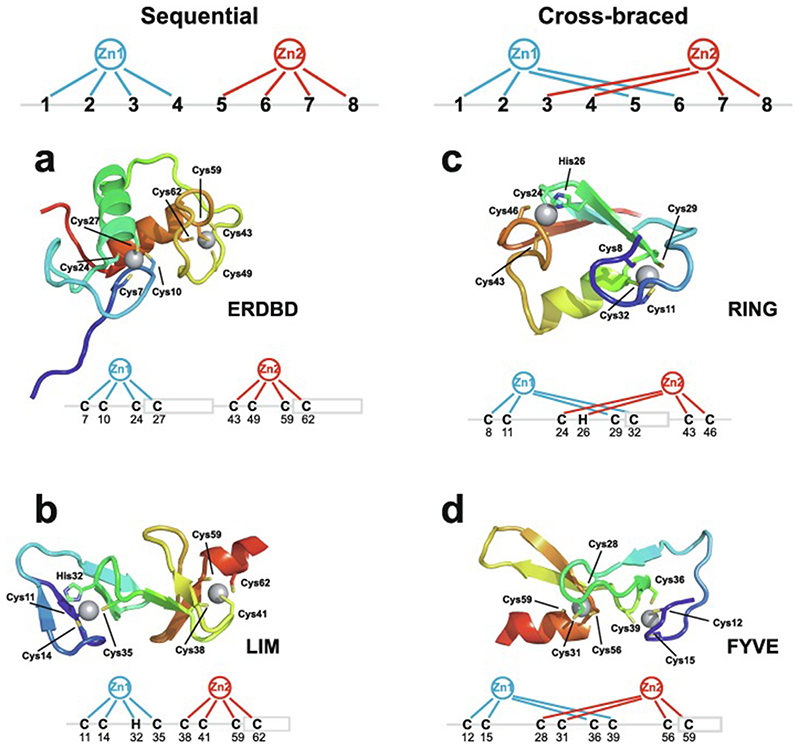
Examples of “sequential” and “cross-braced”metal-binding topologies for zinc finger domains binding two zinc ions. Domains with “sequential” topologies include **a**) steroid hormone DNA-binding domains, exemplified here by the ERDBD [27], as well as **b**) LIM domains, exemplified here by the C-terminal LIM domain of cysteine-rich protein-2 (CRP-2) [165]. Domains with “cross-braced” binding topology include **c**) RING domains, exemplified here by that from equine herpes virus-1 immediate early gene product (EHV-1) [166], and **d**) FYVE domains, exemplified here by that from human EEA1 protein [167]; other examples ofcross-braced topologies include PHD fingers (e.g. [40], see Fig. 2c) and MYND [168,169] domains.

As pointed out earlier, there is a hierarchy of questions that need to be answered:

i) Which are the residues that bind zinc?
ii) For zinc fingers that bind more than one zinc, which ligand binds to which zinc (i.e. what is the metal-binding topology)? A relatively frequently encountered example of this type of ambiguity is the distinction between so-called “sequential” and “cross-braced” binding topologies, illustrated in Fig. 10.
iii) For any histidines that bind zinc, which ring nitrogen (N^δ1^ or N^ε2^) is co-ordinated to the metal?

### 5.1 Working without exchanging zinc

As will be seen, if it is possible to substitute the bound zinc(s) with an NMR-active isotope, normally ^113^Cd, this has the potential to provide definitive answers to these questions. However, this may not always be possible or practicable, so the following section discusses approaches that can be used while retaining the native, but NMR-silent, zinc ions of the natural protein, and the possible limitations on the conclusions that can then be drawn.

#### 5.1.1 Using multiple sequence alignments

The starting point for identifying the zinc-ligating residues in a zinc finger protein is almost invariably consideration of multiple sequence alignment data. In the case that there is only a single zinc bound and only four candidate Cys or His ligands in the sequence, then there is essentially no ambiguity to be resolved (excepting the rare occurrence of zinc co-ordination by an Asp residue), but as soon as more than four potential ligands exist then it becomes necessary to establish which actually bind the metal. The latter situation will almost always be the case, since free cysteines and histidines are very commonly present in addition to the metal-binding residues in zinc finger proteins, and these too may sometimes be well conserved across multiple sequence alignments.

As described in Section 2.1, the condition that all four candidate ligands for each zinc should be present across all members of a multiple sequence alignment is particularly powerful, as is illustrated by the cases of the Rds3 and Bud31 multiple sequence data shown in Fig. 4. In both cases the true metal-binding ligands (all Cys residues in both these cases) are absolutely conserved across all sequences, whereas several of the other Cys or His residues that appear in the yeast sequences actually studied are very poorly conserved or not conserved at all (Cys77 and His100 in Rds3, and His56, 143 and 147 in Bud31) and can thus easily be discounted. The remaining potential candidates, His4 in Rds3 and His57 in Bud31, while not absolutely conserved, show a much higher degree of conservation, such that they might be mistakenly thought to be fully conserved on the basis of a less extensive alignment. It is still relatively easy to discount His4 in Rds3, since there are three zincs bound (as determined by mass spectrometry [48]) and twelve absolutely conserved cysteines, strongly suggesting the presence of three C_4_ zinc sites, and in addition the NOE restraint list clearly shows that His4 lies in a disordered *N*-terminal tail. In contrast, the case of His57 in Bud31 is less clear-cut, and illustrates the limitations of what can be deduced from the sequence and stoichiometry alone. Mass spectrometry shows clearly that the protein binds three zincs (see Fig. 5), but there are only nine absolutely conserved cysteines in the sequence. This could be sufficient to bind three zincs if three of the cysteines each bridge between two zincs (as seen in the structure of the three-metal cluster of metalloth-ionein), but a priori it could also be that fewer cysteines bridge zincs if one or more histidines participate in zinc binding; from these data alone one cannot tell these possibilities apart.

#### 5.1.2 Using chemical shift data

**Figure 11:**
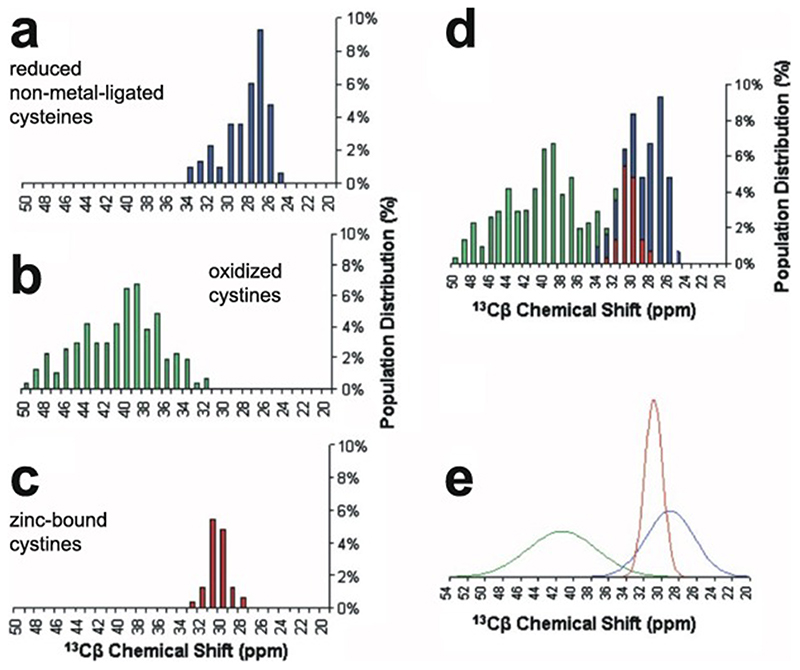
Distribution of C^β^chemical shifts for metal-binding, free (reduced, SH) and oxidised (disulfide, SAS) Cys residues. (**a–d**) Histograms and (**e**) normal distribution curves for 311 cysteine/cystineC^β^ shifts grouped into three categories based on three states of the thiol;(**a**)102 from reduced non-metal-ligated cysteines,(**b**)166 shifts from oxidized cystines, and (**c**) 43 from Zn- ligated cysteines. Adapted with permission from [172] (© 2006 Springer).

Potentially, chemical shift information can sometimes provide another source of information that can help identify metalbinding ligands, once the spectrum has been fully assigned. Although some early papers on TFIIIA-type fingers identified an extensive chemical shift fingerprint for this particular structural family [170,171], and other publications (e.g. on the TAZ2 domain of CBP [111]) used comparative chemical shift data to help build a case for assignments, it took some time before a sufficient number of studies of different types of zinc fingers were available that an informative overview of the information available from chemical shifts could be reached. In 2006 Kornhaber *et al.* published a paper on the diagnostic possibilities of analysing ^13^C shifts in Cys residues to detect which of them bound zinc, based on a survey ofdata in the BMRB database at the time [172]. Fig. 11 shows the distribution of C^β^ chemical shifts seen for different oxidation states of Cys in that study, showing that although the oxidised form (i.e. Cys in disulfide bonds, which as discussed in Section 1 are very unlikely to be present in the same protein as zinc-bound Cys) had a distinctive shift range, the range for metal-bound Cys overlapped strongly with that for free, reduced Cys (i.e. Cys with a free SH group, which are very frequently found in the same protein as zinc-bound Cys). Thus, no simple and robust test for metal binding is available from inspection of C^β^ chemical shifts alone, but the authors did propose a more complex multivariate test based on analysing both C^β^ and C^α^ shifts in conjunction with knowledge of local secondary structure that allowed a reasonable degree of prediction (the authors stated that for predicting zinc ligation and cysteine oxidation state, their method had a recall, precision and F-measure of 83.7 % and an accuracy of 95.1 %). Overall, it seems (at least to this author) that reliable identification of cysteines that participate in zinc-binding in a novel zinc finger of unknown structure based on chemical shift data alone would be risky, at best.

**Figure 12:**
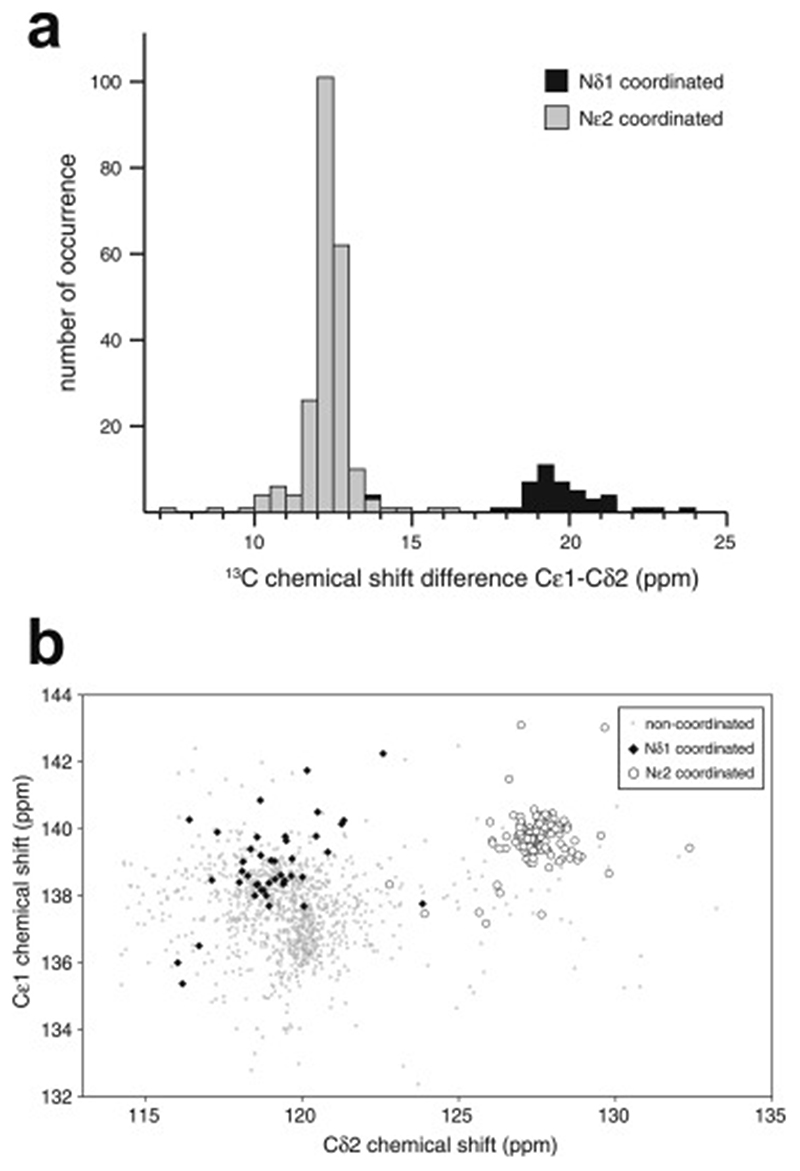
Distribution of ^13^C^δ2^ and ^13^C^ε1^ chemical shifts for histidines co-ordinating zinc through either N^d1^ or N^e2^, compared to free histidine. **a**) Number of occurrences of each histidine–zinc coordination mode as a function of the chemical shift difference δ(^13^C^ε1^)–δ(^13^C^δ2^),plotted in 0.5 ppm intervals. **b**)Two dimensional plots of ^13^C^δ2^ and ^13^C^ε1^ chemical shifts for non-coordinated histidines (small open circles), N^δ1^ zinc-coordinated histidines (black diamonds) and N^ε2^ zinc-coordinated histidines (large open circles). Adapted with permission from [173] (© 2012 Springer Science Business Media BV).

In contrast, some quite clear-cut distinctions can be drawn in the case of histidine ligation, provided that reliable, sequence-specific assignments have been made for the imidazole ring ^1^H^δ2^, ^13^C^δ2^, ^1^H^ε1^ and ^13^C^ε1^ signals. In 2012 Barraud *et al.* published a paper showing that the difference between the ^13^C chemical shifts of the two proton-bearing ring carbons, C^δ2^ and C^ε1^, was characteristically different for histidines that bind zinc through their N^ε2^ atom as opposed to their N^δ1^ atom (Fig. 12a) [173]. This is important information, as it is vital to know which of these atoms isused by each zinc-binding histidine in a zinc finger protein when determining its structure by NMR. Unfortunately, however, these particular shifts do not allow such a definitive conclusion in terms of distinguishing zinc-binding from non-zinc-binding histidines, as the distribution of ^13^C chemical shifts for non-zinc-binding histidines is significantly wider (Fig. 12b).

**Figure 13:**
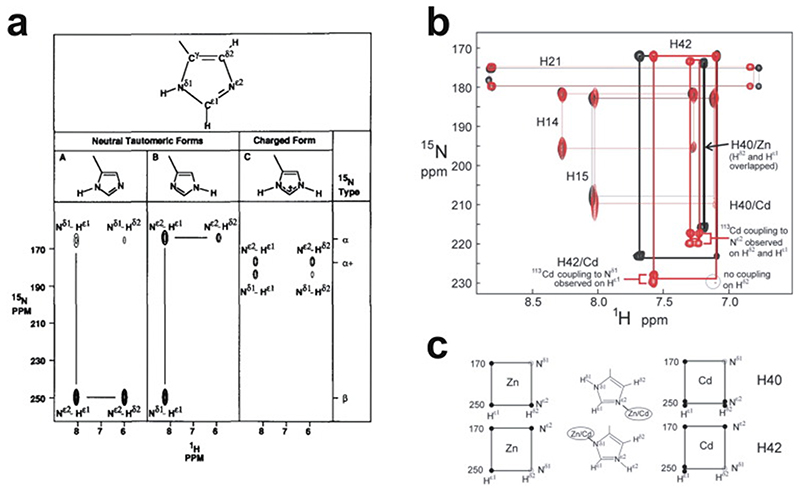
Long-range HMQC experiments for distinguishing tautomeric states and zinc-binding sites in histidines. **a**) Schematic diagram showing the three possible protonation states ofhistidine rings,and the expected long-range ^1^H-^15^N HMQC spectrum ofeach species. The diagramswere constructed using^15^N chemical shift, ^2^JNH and ^3^JNH coupling constant data for histidine taken from reference[174]. For the charged species, the spectrum shows the N^d1^ signal slightly downfield ofthe N^e2^ signal, but in practice either nitrogen could resonate downfield of the other, and the relative assignment must be made through comparison of cross-peak intensities. Reproduced with permission from[175] (© 1993 The Protein Society). **b**) The 500 MHz ^1^H-^15^N HMQC spectrum of the ZZ domain of murine CREB-binding protein (CBP) complexed either to zinc(black)orto^113^Cd(red).**c**)Schematic of the connectivities for the two zinc-ligand histidine residues of CBP,His40 and His42,showing how interpretation of the spectrum in **b**) confirms the identity of the ligand histidine residues and the location of the metal coordination on the histidine ring in each case. Panels c and d reproduced with permission from[176] (© 2004 Elsevier).

An alternative diagnostic for this latter distinction may come from analysing the ^15^N chemical shifts of the N^δ1^ and N^ε2^ atoms, but these generally require a dedicated, long-range-optimised HMQC experiment for their detection and assignment. Early stud-ies using ^15^N direct detection for concentrated samples of isolated histidine established characteristic patterns of ^15^N chemical shifts for the different tautomers and protonation states of histidine as a function of pH, as well as the pH-dependent values for the small two- and three-bond ^15^N-^1^H *J*-couplings within the histidine aromatic ring [174]. In 1993, Pelton *et al.* used these data when studying the (rather strangely named) protein III^Glc^ to establish fingerprints for the different tautomeric forms of histidines as seen in ^15^N-^1^H HMQC spectra (Fig. 13a) [175], and in 2004 Legge *et al.* used a similar approach to determine the tautomeric states and zinc-binding status of histidines in the ZZ domain of CBP (Fig. 13b) [176]. The key points when interpreting such spectra are that:

i) Provided the H^δ2^ and H^ε1^ signals are not overlapped, the N^δ1^and N^ε2^ signals can usually be distinguished in the long-range HMQC spectrum since the N^ε2^ resonance shows reasonably strong cross-peaks to both H^δ2^ and H^ε1^, whereas the N^δ1^ resonance generally only shows a strong cross-peak to H^ε1^; this is because the three-bond coupling between N^δ1^ and H^δ2^ is much smaller than any of the two-bond couplings responsible for the three relatively strong cross-peaks;
ii) A ^15^N atom bearing a proton gives a much more shielded signal (in the range roughly 160–190 ppm) than does a ^15^N atom with no proton (in the region of 250 ppm);
iii) A ^15^N atom bound to zinc gives a signal at a shift (approx. 230–215 ppm in this example) much more similar to that of a non-protonated ring nitrogen than that of a protonated ring nitrogen, but distinct from both.

In the event, the authors used ^113^Cd substitution to establish definitively which histidines bound zinc, and for those that did which ring nitrogen was directly attached to metal, since the presence of the spin ½^113^Cd causes a new splitting in the directly-bound ^15^N signal as a result of the one-bond ^113^Cd-^15^N J-coupling (Fig. 13b). Experiments with ^113^Cd are considered in Section 5.2.2, but these spectra are included here because they also show how an assignment of His40 and His42 as the zinc-binding histidines could have been made in this case, albeit slightly more tenuously, even without data from the ^113^Cd substitution experiments, given that the histidines that do not bind zinc (His14, His15 and His21) are partly or completely in the fully protonated state under the experimental conditions used (pH 6.8) and therefore show patterns with much smaller ^15^N shift differences between the N^d1^ and N^e2^ signals. This is not to advocate disregarding the possibility of using ^113^Cd substitution to make definitive assignments, but rather to point out that in cases where ^113^Cd substitution fails or is impractical, ^15^N-^1^H long-range correlation experiments may still be useful in identifying the zinc ligands in a zinc finger protein based on the observed ^15^N chemical shifts. An example where exactly this approach was used in practice is provided by NMR structure determination of the embryonic neural inducing factor Churchill, for which ^15^N-^1^H long-range correlation data clearly established which histidine binds zinc through N^d1^, which through N^e2^, and which does not bind zinc at all [57]; the last of these assignments was further corroborated by the large pH-dependent shift varia-tions seen for the^1^ Hand ^15^N ring signals of this particular histidine but not the others.A similar approach was used by Malgieri et al.to determine which histidines bind zinc, and through which nitrogens, in the Ros87 DNA-binding domain [177,178], and also by Massiah et al. to determine which nitrogens are used by the zinc-binding histidines in the B-box-1 and B-box-2 domains of the E3 ubiquitin ligase MID1 [179,180].Likewise,long-range^15^N-^1^H HSQC spectra were used analogously to determine the nitrogen-zinc connectivities in two unusual metallothioneins whose structures include C2H2 centres, the bE domain of wheat Ec-1 protein [181]and the bacterial metallothionein PflQ2 [182].

#### 5.1.3 Using preliminary structures

None of the methods discussed in Sections 5.1.1 or 5.1.2 (or indeed 5.1.4) can provide any information at all concerning the metal binding topology, that is to say the question that arises for any zinc finger that binds two or more zincs: “which ligand binds to which zinc?” Examples of zinc-binding topology are shown beneath each of the structures in Figs. 2 and 3, and as was discussed in Section 4, it is absolutely essential to know the correct topology when setting up structure calculations that include explicit representation of the zinc ions in the co-ordinates. The most conclusive approach, when it can be brought to bear, is to substi-tute the bound zinc ions in the protein with a spin ½ nucleus such as ^113^Cd, and this approach will be discussed in Section 5.2. However, in favourable cases it may be possible instead to use preliminary structures calculated without explicit zinc or any input related to identification of the zinc ligands, in order to deduce what the zinc-binding topology must be. One ofthe first examples where this approach was employed was for the “cross-braced” topology of the RING finger domain [67,166].

**Figure 14:**
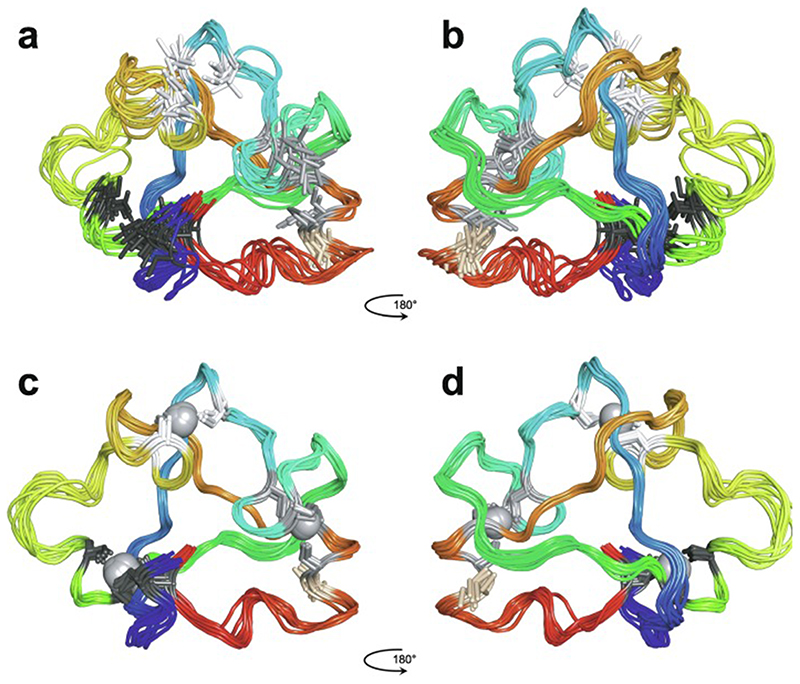
Ensembles of structures of the yeast protein Rds3 calculated without (a and b) and with (c and d) zinc-binding and inter-ligand restraints. **a**) and **b**) Two views (related by a 180°rotation) of the 10 lowest energy structures from an ensemble of 20 calculated with no zinc-ligand or inter-ligand constraints, and no NOE cross-peak assignments in the input data (NOE assignments were made automatically during the calculations, which employed the program ARIA [130]). Smoothed protein backbone cartoons are shown in chainbow colouring from residue Met10 (blue) to residue Leu90 (red), and the cysteines involved in metal binding are coloured white for binding site 1 (Cys23, 26, 58 and 61), light grey for binding site 2 (Cys30, 33, 73 and 76) and dark grey for binding site 3 (Cys11, 46, 49 and 86). The binding sites are most clearly seen in **a**); panel **b**) shows that Cys77 (shown in wheat), although sequentially adjacent to binding site 2, projects away from it. Adapted with permission from[48] (© 2008 National Academy ofSciences). Views **c**) and **d**) show corresponding views ofthe ten lowest energy structures from the final ensemble of 100 calculated NMR structures, in which all zinc binding (and inter-ligand) restraints were applied. Disordered tails at the N- and C-termini are omitted for clarity in all views. These views were produced using the program PyMol[23,24] and are shown in “chainbow” colouring (blue → red, *N*-terminal → C-terminal), zinc ions are shown as grey spheres.

To illustrate this approach, Fig. 14 shows the results of such calculations for the case of Rds3, as compared to the final structures calculated with all the zinc-related restraints included. Even though the “zinc-free“ structures have quite low precision in parts, and many of the cysteines are highly disordered and not oriented towards where the zincs “ought” to be, the three zinc coordination sites are clearly separated and easily identified. The point is reinforced by the compilation of C^β^-C^β^ distances shown in Table 1; even for a structural ensemble calculated with no inter-ligand or zinc-binding restraints at all, distances between Cys C^β^ atoms associated with different metal sites are all significantly longer than any distance associated with a single metal site, thereby establishing the metal-binding connectivity pattern unambiguously. Distances to Cys C^β^ atoms are preferred for use in this analysis since, unlike those of the Cys S^γ^ atoms, the C^β^ atom positions are relatively unaffected by sidechain disorder, so yielding a much clearer result.

Clearly, the reasons why this approach succeeds in the case of Rds3 are that the three zinc-binding sites are well separated from one another in the structure, and in addition that the NOE restraint set is sufficiently dense that the structure is reasonably well-formed even in the absence of zinc-binding restraints. In general, if either of these conditions is not met then one can expect the outcome of such preliminary structure calculations to be inconclusive or ambiguous. It is also important that the restraint set used for the “zinc-free” calculations should be directly derived from the experimental NMR data, and not include, for instance, assignments for ambiguous NOEs based on preliminary interpretations, as these could bias the outcomes. The approach is clearly not risk-free, and indeed has on at least one occasion led to an erroneous struc-ture (the MYND domain from the transcriptional regulator DEAF-1) that was subsequently withdrawn[183,184], though it should also be pointed out that the same group later published a corrected structure, which they were able to determine using significantly improved spectra from a shorter construct of the same protein domain [169]. The method is likely to be unsuited to cases involving metal clusters (as in Bud31), since here the distances between individual metal-binding sites within a cluster are very much smaller than those in the example shown here, and it would likely be impossible to distinguish the binding topology within a cluster (however, as mentioned in Section 5.1.4, the protein Churchill rep-resents an exception to this statement[57]). Nonetheless, in cases where the metal-binding sites are independent and the structure is well-formed in solution, there is a reasonable chance that preliminary structures will have sufficient resolution to establish the topology. Indeed, in cases where metal substitution fails there is no other method available.

#### 5.1.4 .Using mutations

Once one or more hypotheses as to which ligands are involved in zinc binding have been reached using some combination of the approaches described in Sections 5.1.1 – 5.1.3, a very commonly used method to test or confirm these is to mutate specific residues to see if the resulting proteins retain the metal-bound, folded structures of the wild type. If residues thought to bind the zinc(s) are mutated, one would normally expect this to result in partial or complete unfolding, or at least substantial destabilisation of the folded state. This can be tested, for instance using CD spectroscopy or in a binding assay with an interaction partner (unless the protein fails to express, which may in itself be evidence for instability). However, it may be more efficient to mutate Cys or His residues that are proposed *not* to be involved in some suggested metal-binding topology, so as to show that in these mutants the properties of the folded domain are substantially unaffected. This is particularly true for cysteine residues in cases where there are more cysteines present in the sequence than are required for metal binding. Non-metal-binding cysteine residues, i.e. Cys with a free -SH group, are not uncommon in intracellular proteins, but if present in zinc finger domains would most often be greatly out numbered by metal-binding cysteines; probably the commonest situation is for there to be just one additional Cys, as seen for Rds3 (see Section 5.1.1). Mutation of Cys to Ala is oftenused(cho- sen because free cysteines are hydrophobic in nature and often occur in the core of a protein structure), but other mutations have also been used (see e.g. [57]). In the case of His residues the situation is rather different, as the number of metal-binding His (if any) is very often much lower than the number of metal-binding Cys, and they are often outnumbered by non-metal-binding His residues.

In order to test a proposed binding scheme as completely as possible it is necessary to find compelling evidence relating to each proposed ligand residue individually, to establish that it actually does bind metal, implying that it may be necessary to mutate every proposed ligand residue separately unless the availability of other evidence makes this unnecessary for some specific cases. In contrast, if candidate Cys or His residues that are proposed not to be involved in metal binding are mutated, mutating more than one, or possibly even all of them, at once may give a clear result. However, there are clear potential caveats for either approach: it is not impossible that mutating non-metal-binding Cys or His could perturb a structure sufficiently for the interpretation to be unclear, while in structures of some larger zinc finger domains, where a lower proportion of the total folding energy may come from metal binding, it could be that mutation within one metal-binding site may be insufficient to destabilise the protein sufficiently to yield a clear outcome.

Publications where mutations have been used to test proposed metal-binding schemes are not trivial to search for as the mutational analysis is often only mentioned extremely briefly in the text or as “data not shown”, while in many cases it may have been carried out in the original biochemical studies for a given domain rather than as part of a structural study. However, a couple of brief examples serve to illustrate the main points made here. In their study of the embryonic neural inducing factor Churchill, Lee *et al.* used point mutations of individual metal-binding Cys and His residues (not listed in full in the original publication) together with preliminary structure calculations to establish the zinc-binding topology of this highly unusual zinc finger domain, which is the only example known to this author where the binding topology of a zinc finger involving a metal cluster has been determined without using ^113^Cd substitution and heteronuclear correlation experiments. In an example of mutating non-metal-binding residues, de Guzman et al. simultaneously mutated four cysteines thought not be involved in zinc binding, out of a total of 13 present in the TAZ2 domain of cyclic-AMP response element binding protein (CBP), and showed that the resulting quadruple Cys → Ala mutant still bound three zinc ions and maintained its structural integrity [111].

All this said, it must be remembered that mutation cannot establish the actual topology of metal binding in cases where more than one metal is bound. In common with the methods discussed in Sections 5.1.1 and 5.1.2, it is fundamentally incapable of distinguishing which metal binds to which ligand.

### 5.2 Substituting zinc by ^113^Cd

Given the fact that the zinc in zinc finger proteins is effectively “NMR-silent”, one of the most powerful strategies (when it works) for defining metal-binding topology in cases of ambiguity is to replace the zinc with a different metal that has more amenable NMR properties. The obvious choice is cadmium, which lies immediately below zinc in group 12 of the periodic table; using cadmium substitution for NMR work with metalloproteins was first suggested by Armitage in 1976 [185]. Of all metals it is the one with chemical properties most similar to those of zinc, and in addi-tion it has two isotopes with spin ½ that are capable of generating high-resolution NMR data. Table 2 collects a number of relevant parameters for isotopes of zinc and cadmium.

Historically, ^113^Cd has been the isotope most widely used to substitute for zinc in heteronuclear studies of metal binding in zinc finger domains, presumably because it has a slightly higher sensitivity than the other spin ½ isotope ^111^Cd. It is commercially available, most conveniently in the form of soluble cadmium salts such as CdCl_2_ and CdSO_4_ (which as heavy metal compounds of course require careful handling, being highly toxic). Although in principle natural abundance cadmium could be used for some experiments, the roughly 8-fold sensitivity advantage of using purified ^113^Cd for heteronuclear NMR generally justifies the extra expense. Using ^113^Cd also avoids any potential complications due to the second spin ½ isotope ^111^Cd, which at natural abundance would be present at roughly equimolar concentration to ^113^Cd and which resonates at a sufficiently different Larmor frequency that simultaneous pulsing of both isotopes (e.g. for decoupling from ^1^H) is not conve-niently possible. Useful reviews of the early literature of ^113^Cd NMR of proteins and co-ordination compounds have been published by Ellis [192]and Summers [193].

The chemical similarity of zinc and cadmium stems from their similar electronic configurations (both d^10^) and oxidation and charge states (both M(II), i.e. M^2+^, when in zinc finger proteins). However, there is a difference in that cadmium is slightly larger than zinc. Atomic and ionic radii can be defined in many different ways, often based on crystal data for relatively simple ionic compounds, and the apparent differences between zinc and cadmium radii can vary considerably according to which definition is used. Probably the most directly relevant measure where zinc finger structures are concerned is the difference in average metal-sulphur bond lengths reported for Zn_7_- and Cd_7_-metallothionein measured using EXAFS [191], which differ by approx. 8.5 % (2.34 Å for Zn, 2.54 Å for Cd, see Table 2). This is not a particularly large difference, but it is sufficient that one would in general expect some small atomic movements in the structure of a zinc finger domain would be needed in order for cadmium to be accommodated in place of zinc, in turn resulting in some decrease in thermal stability of the folded structure (see e.g. [49]), as well as some changes of ^1^H, ^13^C and ^15^N chemical shifts for nearby parts of the protein structure (see e.g. [59]). This is indeed the case, and typically a proportion of the NMR resonances of a protein would need to be re-assigned if zinc has been replaced by cadmium. However, it is worth recalling that the purpose of substituting the metal is usually not to determine a high-resolution structure of the cadmium-bound protein itself, but rather to determine the correct metal-binding topology so that this can be used as input when calculating a high-resolution structure of the *zinc*-bound protein. For this purpose, the assignments actually needed for the cadmium-bound protein are likely to be just those of nuclei that interact with ^113^Cd and their nearest neighbours, i.e. those of the metal-binding residues themselves. Of course, to make these assignments securely it would probably be necessary to reanalyse a significantly larger number of resonances from nearby parts of the structure and/or overlapping signals, but this task would likely still be well short of having to re-assign the entire spectrum.

The following two sections deal first with the difficult issue of how suitable samples in which native zincs have been exchanged for ^113^Cd can be made, and subsequently how ^113^Cd NMR experiments can be used to define the metal-binding topology.

#### 5.2.1 Preparing ^113^Cd-exchanged samples

While cadmium substitution is undoubtedly an extremely powerful approach to determining metal-binding topology in zinc fingers, it seems that preparing suitable metal-exchanged samples for NMR may have presented something of an obstacle to its more widespread application. However, this is not necessarily well-reflected in the literature, since if a cadmium-based approach was either not attempted or did not succeed this is unlikely to be reported or discussed. One reason for difficulties is that clear guidance on how best to achieve cadmium substitution is quite elusive, with different papers sometimes appearing contradictory. For instance, useful information sometimes comes from publications investigating how treatment of zinc fingers with xenobiotic (i.e. non-native) metal ions such as Cd^2+^, Pb^2+^, Cu^2+^, Ni^2+^, Co^2+^, Mn^2+^, etc. can disrupt their biological function via metal exchange, but it can be less than obvious how to reconcile such data with descriptions in other papers where preparations of cadmium-exchanged fingers are reported, including for ^113^Cd NMR studies.

A case in point is provided by studies of the transcription factor Sp1 from HeLa cells, which contains three TFIIIA-type C_2_H_2_ zinc fingers and was the first case in which zinc was successfully exchanged for cadmium in fingers of this type (though not as part of an NMR study in this instance). Kuwahara and Coleman used dialysis of zinc-bound Sp1 against 50 μM Cd(II) at 4 °C to achieve complete replacement of the metal, and went on to use an electrophoretic mobility shift assay (EMSA) to show that the DNA-binding properties of cadmium-bound Sp1 were essentially identical to those of the zinc-bound protein [88]. These results clearly imply that cadmium-bound protein produced in this way has a near-native conformation with cadmium bound in the same topology as zinc in the native protein, although some experimental details of how this was achieved are unclear (e.g. protein concen-tration and duration of dialysis). In contrast, other experiments in which Sp1 was treated with cadmium in different ways led to loss of DNA-binding function. Kuwahara and Coleman note in their original paper that starting with Sp1 apoprotein is less effective in generating natively folded, cadmium-bound protein, and slightly later Thiesen and Bach [194]showed that pre-incubation of Sp1

apoprotein with a substantial excess of various metal chlorides (1 μM metal chloride vs 1 lM apoprotein), including either CdCl_2_ or ZnCl_2_, results in protein with much reduced ability to bind Sp1′s cognate DNA sequence. Later, Kothini *et al*. showed that treatment of zinc-bound Sp1 with Cd-EDTA complex, or with EDTA-free Cd (II),alsoresultsinlossofDNA-binding ability [195], and in another paper they describe *in vivo* experiments showing that treatment with Cd(II) resulted in dose-dependent inhibition of binding of Sp1 to DNA in mouse kidney cells[196].

Various contrasting interpretations of these results appear in these and other papers, but a plausible view overall would seem to be that the different protocols used to generate the cadmium-bound protein produce varying proportions of material having non-native metal-binding topologies, which consequently lack much or all of the native protein conformation and are biologically inactive. To make matters more complicated still, each of the *in vitro* overall approaches that either partly or completely failed with Sp1 has succeeded for other proteins. For instance, examples of successful cadmium substitution proceeding via addition of Cd (II) to apo-protein include metallothionein [197] and more recently Bud31 [49], while examples of substitution via treatment of zinc-bound protein with Cd-EDTA include CNOT4 and p44[72]. Table 3collects some examples of protocols that have been used to replace native structural zincs in proteins with ^113^Cd in published NMR studies.

One contributory cause for some of these differences lies in the differing relative affinities of zinc and cadmium for cysteinyl sulphur. According to “hard-soft acid-base” (HSAB) theory [214], cadmium ions, being “softer” than zinc ions, are expected to have a stronger preference for relatively “soft” ligands such as cysteinyl sulphur than do zinc ions. This in turn could result in cadmium finding additional Cys binding partners by adopting non-native topologies; for instance, in multiple C_2_H_2_ finger proteins, a cadmium might attach itself to four Cys residues from across more than one finger domain rather than staying within the native C2H2 arrangement of a single finger. Such non-native rearrange-ments have been suggested to underlie xenobiotic metal toxicity involving multi-C_2_H_2_-finger proteins such as TFIIIA [215–217], Sp1 [195,196]and Tramtrack [218], and it has also been suggested that “free”cysteines, i.e. those that do not bind zinc in the native structures, might complicate the picture further [215](see also the discussion of “ambiguous” zinc binding at the end of Section 3.1).

Another consequence of the greater preference of cadmium for sulphur ligands is that the relative affinities of zinc and cadmium for a particular type of binding site should be dependent on the number of Cys residues involved, increasingly favouring cadmium binding in the order C_2_H_2_ < C_3_H < C_4_ [92]. This indeed seems mainly to be reflected in practice; according to the review by Kluska *et al.* [92], C_2_H_2_ fingers generally bind zinc more strongly than cadmium, whereas for C_4_ fingers binding to cadmium is generally stronger than for zinc [72,92]. There can clearly be other sequence-dependent differences in addition, as illustrated by the exceptionally strong zinc affinity of the designed C_2_H_2_ finger CP-1 [64]mentioned in Section 3.1, so this ordering of affinities by class may not hold in every case (see for instance Petering et al. [216]), but it remains a useful guide.

**Figure 15:**
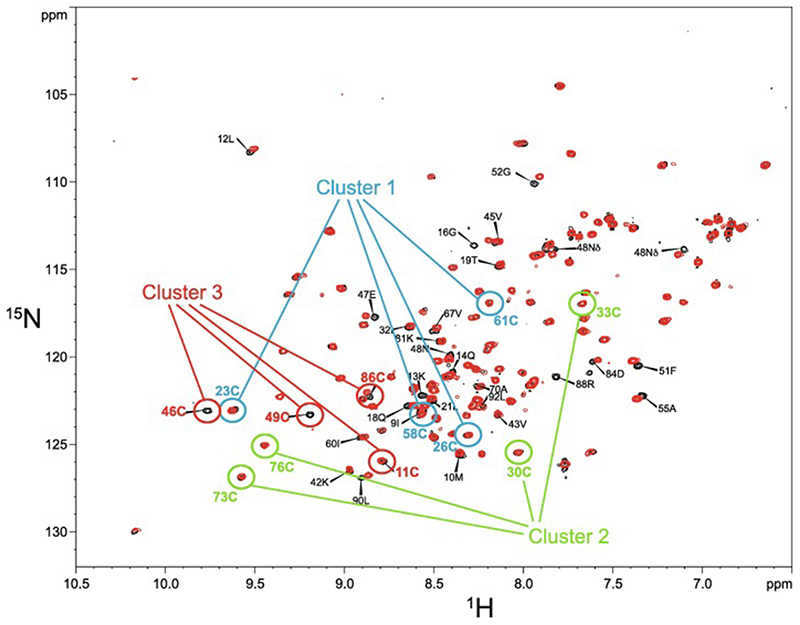
Results of attempted metal exchange for the yeast protein Rds3. Superposition of[^15^N,^1^H] HSQC spectra of zinc-bound Rds3 before (black spectrum) and 1 h after (red spectrum) addition of a fivefold excess of ^113^Cd EDTA complex. Backbone amide correlations corresponding to the metal-binding cysteines are indicated with blue, green and red ovals for binding sites 1, 2, and 3, respectively. Only for the peaks of binding site 3 are there significant chemical shift changes or disappearances, strongly suggesting that it is undergoing metal exchange whereas binding sites 1 and 2 remain substantially unaffected. Some further assignments for peaks that shift are indicated in black, almost all of which are close in space to binding site 3. At later times, the protein partially precipitated and the spectra became somewhat more complicated and much less intense, rendering the intended application of [^113^Cd, ^1^H] correlation experiments essentially impossible. Adapted with permission from[48] (© 2008 National Academy of Sciences).

Replacement of zinc by cadmium was attempted for both Rds3 and Bud31, and the differing outcomes help to illustrate some of the points in this section. In the case of Bud31, ^113^Cd was introduced by slow removal of Zn(II) with EDTA to generate the apoprotein, followed by incubation with excess ^113^CdCl_2_ at low temperature and subsequent removal of the excess (see Table 3); the mass spectra shown in Fig. 5 show that this protocol was successful, and material generated in this way was used in heteronuclear NMR experiments (see Section 5.2.2). In contrast, in the case of Rds3 attempts to produce cadmium-bound protein suitable for heteronuclear NMR by extended incubation of the native zinc-bound protein with excess ^113^Cd EDTA complex (as described by Houben *et al.* for different proteins [72]) failed. Fig. 15 shows the early stages of such an attempted exchange experiment with Rds3. This did demonstrate that one of the three zinc sites of Rds3 exchanged significantly faster than the other two, which was itself of some interest from a structural standpoint, but at longer times complicated mixtures resulted, accompanied by substantial precipitation. One may speculate that the difference between these cases might arise at least in part from a degree of co-operativity during the metal exchange in the case of the metal cluster in Bud31, which might reduce the possibility of multiple, diverging, metal-binding pathways. However, it is entirely possible that a more extended investigation of exchange protocols in the case of Rds3 might have led to a successful outcome; this was not pursued mainly because the metal-binding topology for Rds3 could be unambiguously established using preliminary structure calculations (Section 5.1.3) without the useofcadmium.

Unfortunately, what all of the above makes clear is that there is no fail-safe, general recommendation that can be made for the best way to approach replacing zinc by cadmium in the case of a novel zinc finger. However, there are a few important principles.

1) Exchange is slow, taking place on a timescale of hours. This is implicitly clear from many of the papers referred to in this section, but one in particular demonstrates the point explicitly by measuring exchange kinetics. Houben *et al.* [72] show that for the two C_4_ zinc sites in the RING domain protein CNOT4 the zinc-cadmium exchange rates are roughly 8.7x10^–5^s^–1^ and 4.1x10^–5^s^–1^ (measured at 27 °C and pH 7.0), while for another RING domain, p44, the two rates are roughly 2.1x10^-4^ s’and 3.5x10^–5^s^–1^ ’(measured at 20 °C and pH 7.0).
2) The on-rate for metal binding is proportional to the concentration of free metal ions, whereas the off-rate for dissociation of metal from the protein is concentration-independent. Therefore, working with a low concentration of free metal is likely to favour thermodynamic equilibration, leading (hopefully) to the native binding topology. In contrast, if higher metal concentrations are used, faster initial binding may lead to at least partly stochastic, non-native binding topologies which may then take a much longer time to re-arrange, if indeed they ever do.
3) Metal exchange might continue for as long as appreciable concentrations of excess free metal are present, potentially generating further non-native material. It is therefore advisable to remove excess metals and it may be necessary to purify the desired product after exchange.
4) The greater the proportion of His residues in a native binding site, the more likely it is that addition of cadmium may lead to non-native binding topologies that maximise the number of Cys residues involved in binding after metal exchange. Generating native-like cadmium-bound protein may well be intrinsically more difficult (or sometimes even impossible) for such cases.
5) In this author’s view, papers that interpret impaired biological function, e.g. DNA binding, of zinc finger domains after treatment with cadmium (or indeed other xenobiotic metals) as being due to differences in the protein conformation caused solely by 1:1 metal substitution at the native zinc-binding site should require a high standard of proof that the native metal-binding topology has in fact been retained.

#### 5.2.2 NMR experiments with ^113^Cd

The most useful property of the ^113^Cd isotope where NMR struc-ture determination of zinc finger domains is concerned is that, being itself a spin ½ nucleus, ^113^Cd has scalar couplings with other spin ½ nuclei nearby in the structure. Despite being partially ionic in character, the Cd-S or Cd-N bonds to ligating residues are able to transmit such couplings efficiently, often resulting in coupling constants of up to several tens of Hz and thereby facilitating correlation experiments that link ^113^Cd to its coupling partners. Such experiments are extremely useful in helping to establish metal-binding topology and structure, and they form the main topic of this section.

Much of the development work on these experiments took place during the 1970^0^s and 1980^0^s in the context of structural studies of the protein metallothionein (MT; see Fig. 2e). Although not usually classified as a zinc finger protein, MT has much in com-mon with zinc finger proteins, particularly those that contain zinc clusters entirely bound by cysteine residues, and the experiments developed to characterise metal-binding topology in MT are directly transferrable to studies of such zincfingers. The early work on MT pre-dated the widespread use of global labelling of proteins with ^15^N or^13^C by several years, and the heteronuclear, twodimensional NMR experiments used for correlating ^113^Cd with ^1^H represented some of the earliest applications such methods to proteins, particularly those employing ^1^H detection.

##### 5.2.2.1 Using ^113^Cd-^113^Cd correlations

**Figure 16:**
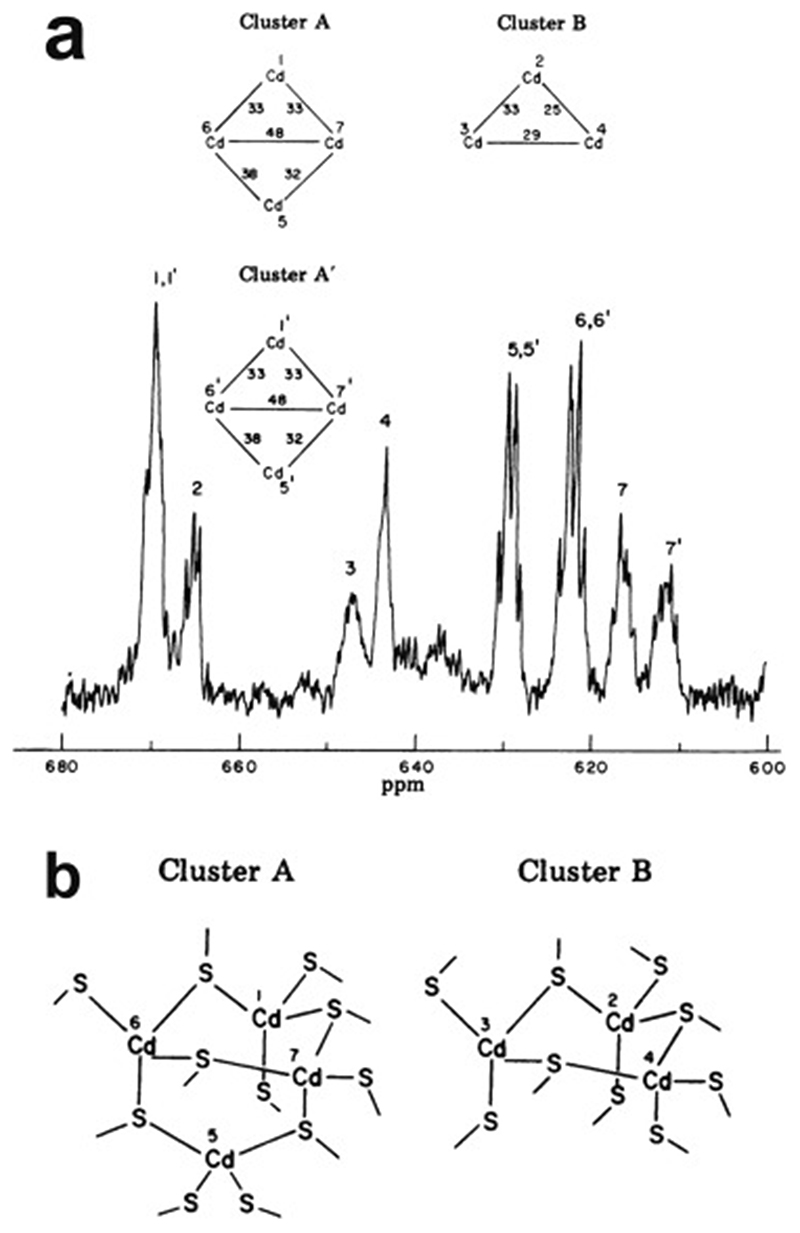
**a**) Rabbit metallothionein isoform MT1 is a small (6.9 kDa), rapidly tumbling protein that gives mostly narrow lines in its ^113^Cd NMR spectrum. Homonuclear J-couplings are clearly visible in the majority of the signals, demonstrating the existence of metal clusters. The cluster topologies shown here were established using a series of selective, homonuclear, one-dimensional ^113^Cd decoupling experiments, and the values (in Hz) ofthe corresponding J-couplings are indicated (the authors attributed the presence oftwo sets of ^113^Cd signals forcluster A to the presence or absence of “ ^3^Cd in cluster B). Conditions: ~8 mM protein in a 10 mm o.d. tube, 10 mM Tris buffer, 100 mM NaCl, pH 9, recorded at 44.4 MHz and 23 °C using 9500 scans and an 8 s recycle time; “^3^Cd chemical shifts are reported in ppm downfield from external 0.1 M Cd(ClO_4_)_2_. **b**) Schematic structures of the four-and three-metal clusters (A and B) in metallothionein, as proposed by Otvos and Armitage[198]. Panels a and b reproduced with permission from Otvos and Armitage[198] (© 1980 Otvos & Armitage).

The first experiments to detect and exploit couplings involving ^113^Cd in proteins were carried out by Otvos and Armitage in 1979. Fig. 16a shows onedimensional ^113^Cd spectra of rabbit metallothionein MT1 in which resolved multiplet structures are visible in most of the resonances. Given that these spectra were acquired with broadband proton decoupling and the only isotope labelling is that with cadmium, it is clear that these splittings must be due to homonuclear ^113^Cd-^113^Cd couplings. This was confirmed by a series of selective, homonuclear decoupling experiments that established the patterns of connectivities and coupling constant values summarised on the figure; such experiments were the method of choice for defining coupling connectivity networks prior to the introduction of two-dimensional experiments such as COSY (later studies did use COSY experiments to detect coupling between cadmiums; see for example [49,200,219,220]). This work provided the first indication that the metals in metallothionein formed clusters, one containing four metals and one containing three, and consequently represented a breakthrough for the area at the time. The structures proposed for these clusters are shown in Fig. 16b; they involve bridging cysteines that simultaneously bind two metals, and it is these that provide the two-bond pathway for transmitting the homonuclear ^113^Cd-^113^Cd couplings through sulphur. However, the spectra also illustrate another feature, particularly of early NMR studies of metallothioneins, that preparation of homogeneous samples with native metal-binding topology was challenging. The existence of a second set of signals attributed to protein containing ^113^Cd only in the four-metal cluster was a problem that was later overcome by improving the preparation protocol (see Table 3).

**Figure 17:**
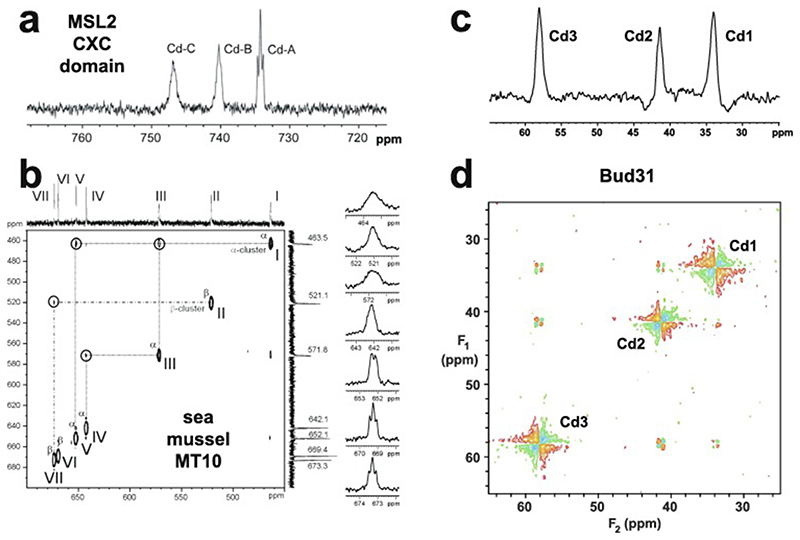
Examples of directly observed ^113^Cd spectra of proteins containing metal clusters substituted with ^113^Cd. **a**) CXC domain of MSL2. In this small (6.0 kDa), single-domain, three-metal cluster zinc finger, significant differences amongst the lineshapes of the ^113^Cd signals are clearly visible, and the ^113^Cd-^113^Cd J-couplings are well- resolved only for signal Cd-A. Conditions: 0.5-1.5 mM protein in 50 mM potassium phosphate, pH 6.0, recorded at 89.13 MHz and 25 °C; ^113^Cd chemical shifts are reported in ppm downfield from external 1 M Cd(CH_3_CO_2_)_2_. Reproduced with permission from Zheng *et al.* [59] (CCBY licence, © 2012 Zheng et al.). **b**) *Mytilus galloprovincialis* (sea mussel) metallothionein isoform MT10. This metallothionein has an atypical four-metal cluster topology, missing one bridge relative to canonical metallothioneins[220]. The ^113^Cd-^113^Cd COSY spectrum shown here reveals only a subset of the expected ^113^Cd-^113^Cd connectivities, presumably at least in part because several of the ^113^Cd signals (shown right) are very broad. Conditions: 2.8 mM protein, 17 mM Tris buffer, 16 mM DTT, pH 7.0, recorded at 133.7 MHz and 25 °C; the COSY data (phase-insensitive) comprised 2 K ×80 time-domain data points and a spectral width of 400 ppm in each dimension, with 1024 scans per increment and a 2 s recycle delay; ^113^Cd chemical shifts are reported in ppm downfield from external 0.1 M Cd(ClO_4_)_2_ in ^2^H_2_O. Reproduced with permission from Digilio et al. [220] (© 2009 SBIC). **c**) Bud31 one-dimensional ^113^Cd spectrum. In this protein the three-metal cluster is fused to a four-helix bundle, resulting in an 18.9 kDa protein that gives much broader ^113^Cd signals than most ofthose in a) or b), and for which the ^n3^Cd-^n3^Cd J-couplings are unresolved. Conditions: 2.6 mM protein in ^2^H_2_O, recorded at 111.0 MHz and 25 °C using 2048 scans and a 1 s recycle time with a cryogenically cooled probe; ^113^Cd chemical shifts are reported in ppm downfield from external neat CdMe_2_ [221]. **d**) Bud31 ^113^Cd-^113^Cd phase-sensitive COSY spectrum (positive contours scaled red to yellow, negative scaled green to blue, phased with cross peaks in double-absorption mode and diagonal peaks in double-dispersion mode). Despite the lack of resolution of the ^113^Cd-^113^Cd couplings in the one-dimensional spectrum, all of the expected connectivities were detectable following a long experiment using a spectrometer equipped with a highly sensitive cryogenically cooled probe. Conditions and chemical shift referencing as for d); the COSY data comprised 256 ×64 complex time-domain data points (t1max 9.51 ms, t2max 614.4 ms), with 1024 scans per increment and a 1 s recycle delay resulting in a 59 h total acquisition time. Panels c and d adapted with permission from van Roon et al. [49] (CCBY licence, © 2015 van Roon et al.), where further experimental details may be found in the supplementary material.

Fig. 17 shows a selection of further spectra illustrating the use of ^113^Cd NMR to detect and analyse cadmium-cadmium connectivities in zinc finger proteins and metallothioneins. A notable feature of all the spectra in Figs. 16 and 17 is the wide variety of linewidths seen for the ^113^Cd signals. This is a matter of some importance, as clearly the sensitivity of any correlation experiment will be significantly degraded for those signals that are broad. For instance, in the COSY spectrum of sea mussel MT10, many of the possible cross peaks are missing (particularly those below the diagonal), and in the COSY spectrum of Bud31 the weakest cross-peaks are those linking the two broadest signals (Cd1 and Cd2). Similar issues arise for the ^113^Cd-^1^H correlation experiments described in Section 5.2.2.2, and in general the variable nature of ^113^Cd linewidths constitutes a major limiting factor on the applicability of the approach of cadmium substitution.

Few if any studies seem to have addressed the origins of such differences in ^113^Cd linewidths in zinc finger proteins or metalloth-ioneins in any quantitative sense. As with any solution NMR application, one obvious consideration is the overall tumbling rate of the molecule studied, and clearly the greater size of Bud31 (18.9 kDa, as opposed to approx. 6–7 kDa for the other proteins shown here) must be a major factor in causing the much broader ^113^Cd resonances. Indeed, it is clear that this particular study only became feasible due to the increased sensitivity available from recently developed, cryogenically-cooled probes suitable for (^113^Cd, ^1^H) double resonance experiments [49]; this does now make it realistically possible to carry out such studies on proteins that are at least somewhat larger than the extremely small domains that featured in earlier studies.

However, the examples shown in Fig. 17 also make clear that different ^113^Cd signals from the same protein can have widely different intensities and linewidths. Pure intensity differences could arise from differing levels of occupancy of metal sites, for instance resulting from incomplete metal exchange during sample preparation (e.g. as seen in Fig. 16a); in principle, this could be tested by other means, e.g. using mass spectrometry (see Section 2.2). On the other hand, ^113^Cd linewidth differences must presumably result from site-specific differences in either relaxation or exchange properties. Dipolar relaxation of ^113^Cd in zinc fingers is unlikely to show particularly significant site-specific differences between different metal sites in the same zinc finger protein, as the proximity to the nearest H^β^ protons of ligating cysteines, which will always be the dominant source of such relaxation, can only vary over a limited range constrained by covalent structure; typi-cally, the combined influence of nearby protons sums to be approximately equivalent to that of a single proton 2.4 Å from the cadmium (this figure was obtained from calculations using the PDB structure of Bud31 and assuming an *r*^-6^ distance dependence). A more plausible origin for site-specific differences in ^113^Cd relaxation properties could be from chemical shift anisotropy (CSA) con- tributions, and this possibility has been discussed [220,222]. CSAis well-established as an important relaxation mechanism for cadmium in general[192,193]. However, in zinc finger proteins if one considers only the directly ligating atoms then the environment is likely to be highly symmetric, at least where all four ligands are cysteine (which is the case for every example shown in Figs. 16 and 17). A perfectly symmetrical tetrahedral environ-ment would result in the complete absence of CSA relaxation, so what CSA contributions there are must arise due either to distortions in the tetrahedral arrangement or to effects from more distant parts of the structure (or, in other proteins, the presence of a mixture of cysteine and histidine ligands). The fact that the range of isotropic ^113^Cd shifts in zinc fingers is relatively limited (as illustrated by those shown in Fig. 16, once large differences in the chemical shift standards used in each case are accounted for) perhaps suggests that such symmetry-breaking perturbations may also be quite limited, so while there seems to be little definite data on this point, the proposal that differences in CSA relaxation account for the site-specific differences in ^113^Cd linewidths seems less than obviously secure.

An alternative origin could be from exchange effects. Exchange of metal between the free and bound states is very unlikely to cause linebroadening, since where the rate of such exchange has been characterised it is on a timescale of hours (see Section 5.1). However, one may speculate there could easily be highly localised conformational exchange processes that are much more rapid, for instance involving concerted small rotations of metal-binding cys-teine sidechains or maybe even exchange between rotameric states coupled with movements of nearby backbone atoms [223]. A distinction between exchange and CSA contributions to ^113^Cd lineshapes would presumably be relatively straightforward to establish experimentally, for instance by measuring temperature and *B0* field dependencies, but to this author’s knowledge no such study has yet appeared.

##### 5.2.2.2 Using ^113^Cd-^1^H correlations.

The previous section discussed how homonuclear ^113^Cd-^113^Cd couplings can unambiguously establish the presence of metal clusters in zinc finger proteins. However, the most powerful structural constraints result from analysing ^113^Cd-^1^H couplings, since these allow sequence-specific identification of the individual residues, particularly cysteines, that bind to each individual cadmium (provided, of course, that reasonably complete and reliable resonance assignments are available for the protons). In other words, they can reveal the metal-binding topology directly.

**Figure 18:**
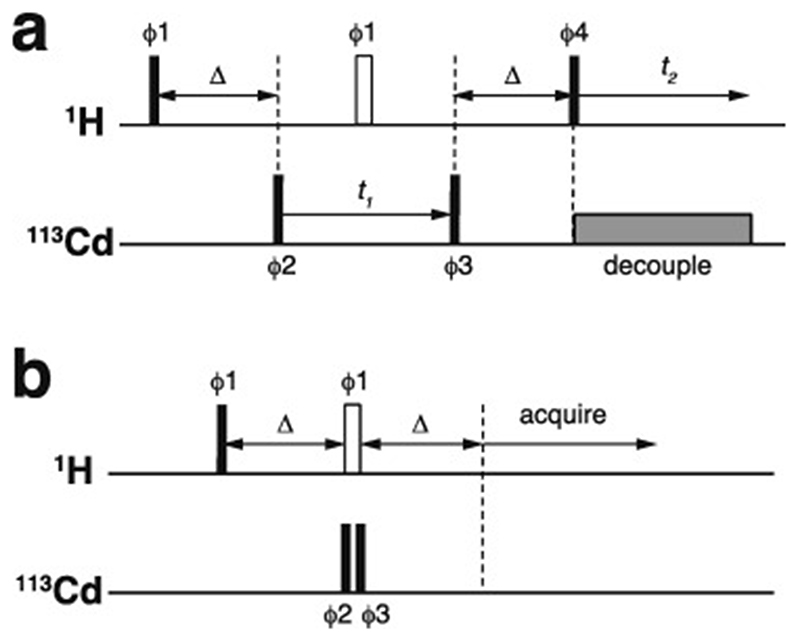
HMQC-related experiments for identifying ^113^Cd-^1^H correlations. **a**) ‘H-”^3^Cd HMQC-RELAY pulse sequence. Filled bars represent 90°pulses, open bars 180°pulses, and the delay **Δ** is tuned for a specific value of J(^113^Cd,^1^H); typical values range between about 50 ms (for J = 10 Hz) and 10 ms (for J = 50 Hz). In the original experiments published by Frey et al.[219], suppression of signals having anti-phase splittings due to (^1^H,^113^Cd) couplings was achieved by a phase- alternated 90° ^113^Cd purge pulse at the start of *t_2_* because on the hardware in use at that time continuous decoupling on the ^113^Cd channel during acquisition was not available. Some other variations of the pulse sequence, for instance replacing the final ^1^H 90°pulse by a z-filter, were also described, though these gave less transfer to the H^α^protons. The version of the sequence shown here is that used by van Roon et al.[49]; the phase cycles used were φ1=x; φ2 = x, -x; φ3 = x, x, -x, -x; φ4=y; receiver = x, -x, -x, x. **b**) Heteronuclear Spin-Echo Difference (HSED) experiment. This one-dimensional experiment suppresses signals not coupled to the heteronucleus, since only the heteronuclear-coupled proton signals change sign when the two X-nucleus pulses reinforce to form an effective 180°pulse; conceptually, it can be looked on as an HMQC experiment from which the indirect frequency dimension has been collapsed. The experiment was originally published as a method for isolating ^13^Cor^15^N satellite signals in natural abundance materials [226].

The most useful ^113^Cd-^1^H couplings are those linking cysteinyl H^β^ protons to ^113^Cd. Experiments to detect and analyse these were published by Frey *et al.* in 1985, based on pulse sequences related to HMQC [219]; other publications using the HMQC sequence itself also appeared at almost the same time[224,225]. These three-bond couplings vary very widely in size over the approximate range 0–75 Hz, which complicates considerably the optimisation of any relevant tuned delays in NMR pulse sequences. Consequently, sometimes multiple datasets are acquired, each one being tuned for a different value of ^3^J so as to maximise the number of correlations that can be detected overall (see for instance [59]). Another issue is that the cysteinyl H^β^ proton signals can be quite crowded in cysteine-rich proteins, potentially making resolution of the cross-peaks in a ^113^Cd-^1^H correlation spectrum difficult. Mainly for this reason the HMQC sequence is often combined with some pulse sequence element that further relays the signal from the Cys H^β^ protons to the Cys H^α^ proton of the same residue; the H^α^ multiplets are much narrower than those of the H^β^ protons since they lack a geminal ^1^H–^1^H coupling, and also there are only half as many, resulting in a much less overlapped spectral region. The experiment most commonly used is the HMQC-RELAY sequence introduced by Frey *et al.* [219], in which a 90°^1^H pulse is added immediately prior to acquisition, thereby achieving an additional coherence transfer through the H^α^-H^β^*J*-coupling without lengthening the pulse sequence (Fig. 18a). As a bonus, this sequence can also transfer coherence between the H^β^protons via the two-step pathway ^113^Cd → H^β^ → H^β^, potentially revealing a second H^β^proton in cases where the direct ^113^Cd-^1^H *J*-coupling to the second H^β^proton is too small for a correlation to be detected. However, all these advantages come at the price of some reduction in sensitivity.

**Figure 19:**
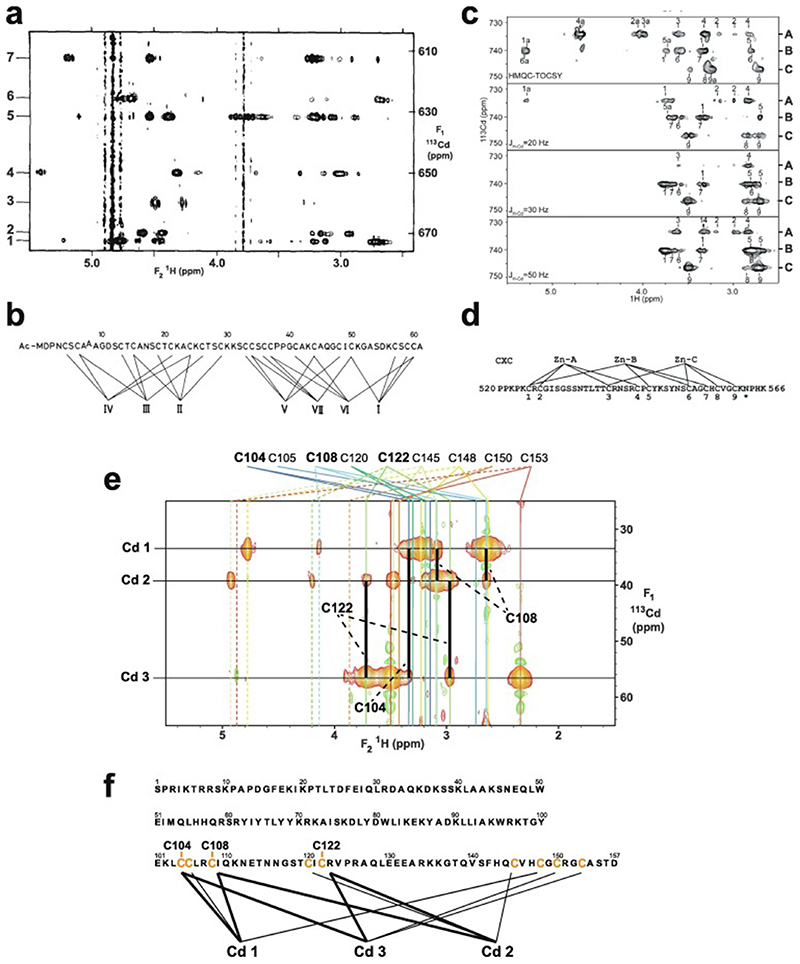
Examples of determining metal-binding to pologyusing^1^H-^113^Cd correlation spectra. **a**)HMQC-RELAY spectrum(Δdelay 30ms)of rabbit metallothionein MT2 [219]. Conditions: 10 mM protein in ^2^H_2_O, 20 mM Tris buffer, 20 mM KCl, p^2^H 7.0, recorded at 360 MHz for ^1^H (80 MHz for ^113^Cd) and 25 °C; ^113^Cd chemical shifts are reported in ppm downfield from external 0.1 M ^113^Cd(ClO_4_)_2_. Adapted with permission from Vašak *et al.* [197] (© 1987 Elsevier). **b**) Metal binding topology deduced from the combined interpretation of this and other HMQC-RELAY spectra, displayed as a function of the amino-acid sequence, reproduced with permission from Wagner et al. [231] (© 1987 Springer Basel AG). Roman numerals used for numbering the cadmiums in panel b correspond to the Arabic numerals used in panel a. **c**) HMQC-TOCSY (Δdelay 10 ms, TOCSY mixing period 30 ms) and HSQC spectra tuned for different ^113^Cd-^1^H coupling values, recorded for MSL2 CXC domain. The Cys residues at positions 525, 527, 539, 544, 546, 553, 556, 558 and 561 are sequentially numbered from 1 to 9, and cross-peaks to H^α^protons in the HMQC-TOCSY spectrum are additionally labelled “a”. Conditions: 0.5– 1.5 mM protein in 50 mM potassium phosphate, pH 6.0, recorded at 89.13 MHz and 25 °C; ^113^Cd chemical shifts are reported in ppm downfield from external 1 M Cd (CH_3_CO_2_)_2_. d) Metal binding topology deduced mainly from the connectivities revealed in spectrum c), displayed as a function of the amino-acid sequence; the connections from Cd-C (the broadest of the cadmium resonances) to Cys 539 and Cys 553 were inferred from preliminary structures calculated without constraints to Cd-C. Panels c) and d) adapted with permission from Zheng *et al.* [59] (CCBY licence, © 2012 Zheng et al.). **e**) HMQC-RELAY spectrum (Δ delay 10 ms) of Bud31. Chemical shift assignments for the cysteinyl H^β^ (solid lines) and H^α^(dashed lines) protons are indicated, using a “chainbow” colouring scheme (blue → red, *N*-terminal → C-terminal) to aid visualisation. Bridging cysteines can be identified since they should show corresponding correlations at two distinct cadmium shifts, as exemplified by the H^b^signals linked by the black lines for Cys104, Cys108 and Cys122. In practice, however, not all correlations can usually be seen in a single experiment; in this case, only one ofthe H^β^protons ofCys104 shows correlations to both Cd1 and Cd3, and a combined interpretation using spectra with other fixed delays as well as HMQC-NOESY data was required to complete the determination of metal-binding topology. Conditions: 2.6 mM protein in ^2^H_2_O, recorded at 500 MHz for ^1^H (111.0 MHz for ^113^Cd) and 25 °C with a cryogenically cooled probe. The data comprised 512 x 16 complex time-domain data points (t_1max_ 3.16 ms, t_2max_ 63.9 ms), with 2048 scans per increment and a 1 s recycle delay resulting in a 22 h total acquisition time; ^113^Cd chemical shifts are reported in ppm downfield from external neat CdMe2[221]. **f**) Metal binding topology deduced from the combined interpretation of this and other HMQC-RELAY and HMQC-NOESY spectra, displayed as a function of amino-acid sequence. Bridging cysteines are labelled above the sequence, and their connectivities are shown using bold lines. Panels e) and f) adapted with permission from van Roon *et al.* [49] (CCBY licence, © 2015 van Roon *et al.*), where further experimental details may be found in the supplementary material.

The HMQC-RELAY experiment and variations on it have been used in several subsequent studies, both for metallothioneins (see for instance [197,220,227,228]) and zinc finger proteins involving metal clusters (see for instance [49,59,206,208,229]). Some have used a homonuclear TOCSY mixing step to make the H^β^→ H^a^ transfer step (though this may cause greater intensity losses with larger proteins), either combined with HMQC [59] or HSQC[227,228], and others have used heteronuclear TOCSY mixing to effect both ^113^Cd → H^β^ and interproton transfers simultaneously in the context of a one-dimensional experiment using semi-selective ^113^Cd pulses to excite a single cadmium signal[229]. It is also possible to use an NOE step for interproton transfer (e.g. by using an HMQC-NOESY pulse sequence [49]), although this potentially introduces significant additional ambiguities in the interpretation as there is no longer any certainty that detected signals necessarily originate in the same residue as the Cys H^β^ proton coupled to ^113^Cd. Usually, interpretation is based on combined analysis of several different experiments so as to maximise the number of useful correlations detected; such experiments may either be opti-mised for different ^113^Cd-^1^H coupling sizes, or use different pulse sequence variations, or both. Zangger and Armitage proposed an “accordion” variant of the HMQC experiment in which the Δ delay is incremented in parallel with *t_1_*, thereby covering a range of values for ^3^*J* (^113^Cd,^1^H) in a single experiment [230]; however, to date this sequence does not seem to have been applied during a published structure determination. Given that many of the above experiments require several hours to yield a reasonable signal-to-noise ratio with protein samples, a useful tip for optimisation is to first run test experiments using a sample of ^113^Cd EDTA complex; details may be found in Live et al. [224]. Fig. 19 shows a number ofpublished examples where ^113^Cd-^1^H correlation experiments have been used to determine the metal-binding topology.

**Figure 20:**
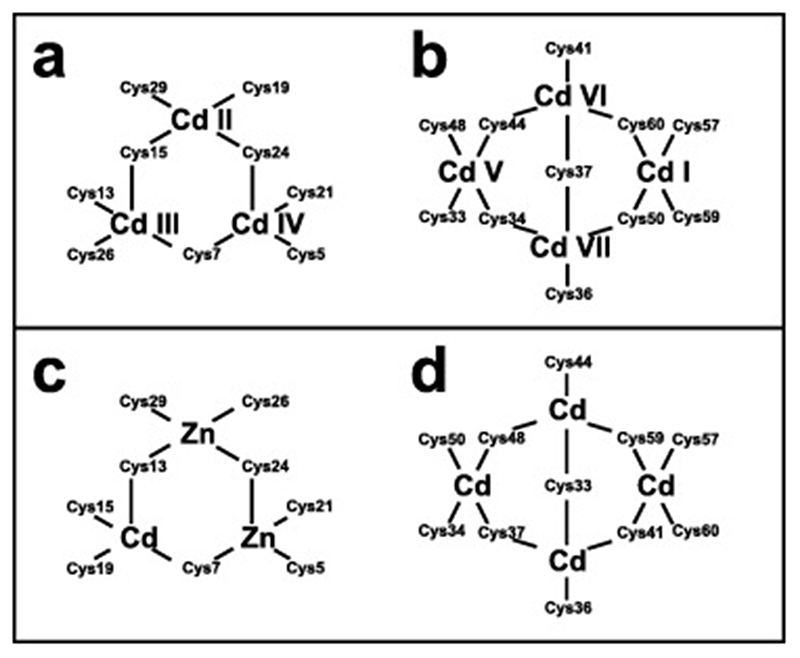
Metal-binding topologies of metallothionein as determined by solution NMR and by the initial X-ray crystallography study. The connectivities shown in **a** and **b** were derived from ^113^Cd-^1^H correlation experiments published by Frey *et al*. [219], while those shown in **c** and **d** are those reported in the originally published crystal structure by Furey *et al.* [235]. Based on Fig. 5 from reference[231] (© 1987 Springer Basel AG).

The first published application of this methodology was to the solution structure determination of metallothionein [219,232–234]. This was quite controversial for a while at the time, since soon after the publication of the metal-binding topology as determined by NMR in 1985, a crystal structure of metallothionein was published in 1986 that showed a completely different metalbinding topology (see Fig. 20) [235]. It took a few years before the resolution of this contradiction was published; it turned out that an erroneous interpretation of anomalous scattering in the diffraction data, which was in turn attributed to pseudosymmetry in the crystal, had led to an incorrect assignment of the space group during the analysis of the X-ray data, and when these errors were corrected the resulting revised structure [236]was highly similar to that derived by solution NMR incorporating the results ofthe ^113^Cd-^1^H correlation experiments [38]. It is probably fair to say that the fact that it was the NMR structure of met- allothionein that proved to be correct, though controversial at the time, contributed in some degree to the widespread acceptance of solution NMR as a valid method of protein structure determination that occurred during this period.

**Figure 21:**
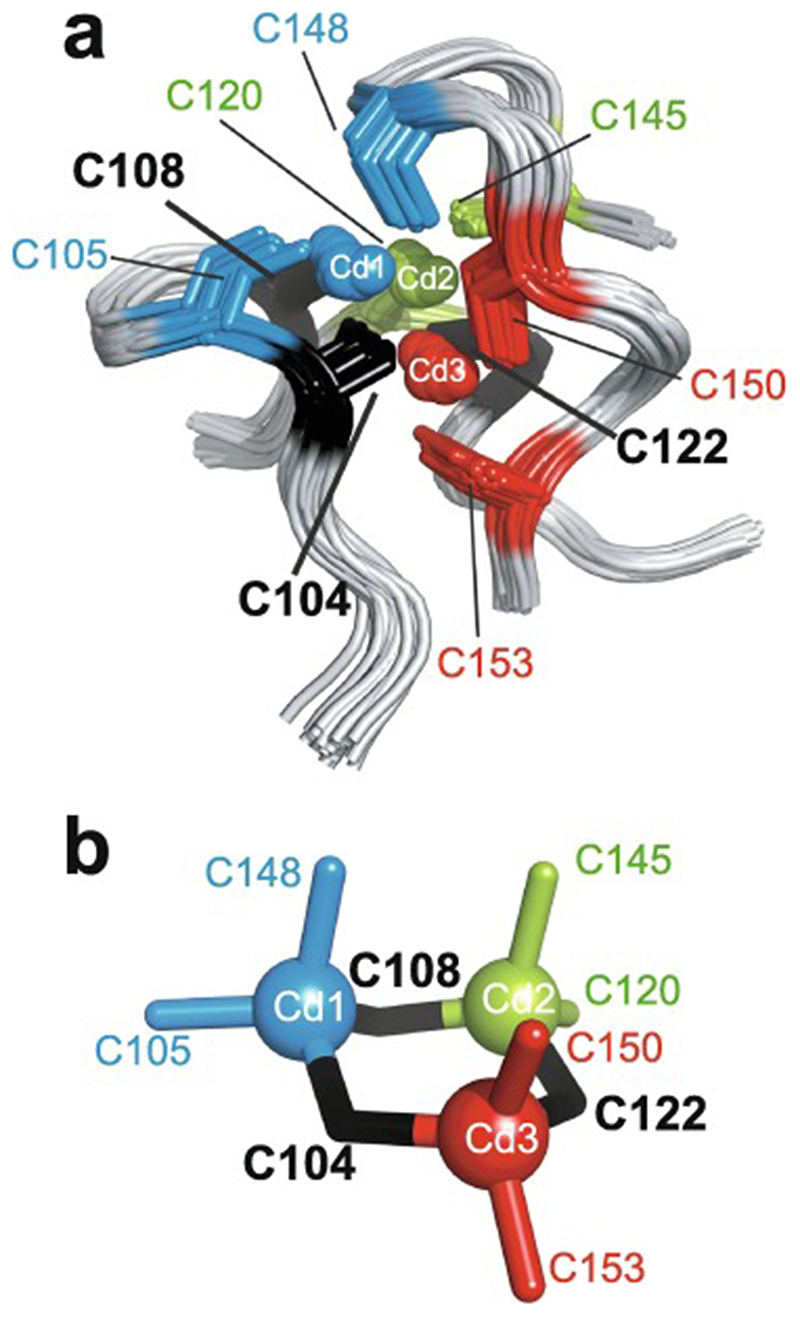
Metal binding cluster ofBud 31. **a**) Ensemble view showing the backbone of residues 101–110,119–126 and 144–154 in cartoon representation together with cysteine sidechains as sticks and cadmiums as small spheres; the ensemble was superposed over the whole ordered region of the protein (residues 12–36,45– 109,118–155). **b**) Schematic of the metal cluster ring in similar orientation to that shown in a), showing only cadmium ions and cysteinyl S^γ^atoms. Colour code is the same as used in Fig.3g and 3 h.

Similar approaches were used in a number of subsequent publications on metallothioneins (see for instance [220,227,233,234,237–241]), and reviews of this area have been published by Vašák [242], Blindauer [228] and Armitage [243]. Applications of ^113^Cd-^1^H correlation experiments to zinc finger proteins are less numerous and appear to date to be restricted to cases involving two- and three-metal clusters (see for instance [49,59,206,207,209,229]). There is no *a priori* reason why the approach should not be applicable to cases where multiple metals are bound at independent sites, but it may be that the preparation of cadmium-substituted samples could be inherently more challenging in at least some such cases, particularly if substitution is co-operative in the case of a cluster but not for independent sites (see Section 5.2.1). It has been suggested that the availability of improved data from higher field spectrometers could make the useof^113^Cd-^1^H correlation-based restraints unnecessary infuture; however, this claim was based on a study of a particularly small metallothionein, MT1 from *Neurospora crassa*, which binds six Cu (I) ions in a single 25-residue mini-domain including 7 cysteines [244], so it seems unlikely to be true in general. Indeed, it is possible that the heteronuclear approach might become more widely used in the future following the advent of cryogenically-cooled probes for ^113^Cd-^1^H double resonance offering improved sensitivity, as was highlighted by the case of the Bud31 study[49]. Fig. 21 shows the relatively precise structural ensemble that results in the region of the metal cluster for Bud31 when explicit metal ions and restraints are used in the structure calculations as discussed above.

While the main structurally useful information extracted by analysis of ^113^Cd-^1^H coupling data is the identification of which cysteine binds to which metal, which requires only the assignment of the two correlated signals in each case, some have also examined the structural significance of the sizes of the *J-*couplings. In 1994, Zerbe *et al*. used data from rat liver metallothionein and *Desulfovibrio gigas* rubredoxin to demonstrate a Karplus dependence of ^3^*J* (^113^Cd,^1^H) on the ^113^Cd-S^γ^-C^β^-^1^H^β^dihedral angle [245,246]. In principle, if sufficiently general this could be used to derive torsion-angle restraints for use in structure calculations, but a subsequent paper on the structure determination of ^113^Cd-substituted GAL4 by Baleja*et al.* [247] showed that the relationship was more complicated, depending also on whether the cysteine was bridging or terminal and probably also on other factors that could distort the local geometry of atoms on the coupling pathway. As far as this author is aware, torsion-angle restraints based on ^113^Cd-^1^H coupling data for zinc finger proteins or metallothioneins have never been used in any published structure calculations.

All of the preceding examples relate to correlations of cysteinyl H^β^ protons to ^113^Cd, but ofcourse many zinc finger proteins involve co-ordination also by histidines. Three-bond couplings of ^113^Cd to either the H^δ2^ or H^ε1^ aromatic protons of the imidazole ring of histidines are likely to be very small, due at least in part to the fact that the corresponding dihedral angle will always be close to zero (assuming that the cadmium binds in-plane with the aromatic ring). Consistent with this, there seem to be very few reports of correlations through these couplings being detected (for examples, see Fig. 5 of [165] and Fig. 7 of [228]).

A somewhat different approach to exploiting ^113^Cd-^1^H couplings is to use heteronuclear filters (here meaning pulse sequence elements, not items of hardware) to identify coupling partners of ^113^Cd during experiments that lack a corresponding ^113^Cd frequency dimension. One of the earliest such methods is the Heteronuclear Spin-Echo Difference (HSED) sequence (Fig. 18b) [226], which exploits the fact that the signs of signals in anti-phase with respect to a coupling to an X nucleus can be reversed by a 180°X-nucleus pulse while other signals remain unaffected (Fig. 18b). This method has been used to detect particularly small ^113^Cd-^1^H couplings, notably including couplings mediated by hydrogen bonds from backbone amide protons to the S^γ^ of metal-binding cysteines. The first such example published was for a cadmium-substituted iron-sulphur protein, rubredoxin from Pyrococcus furiosus [248], but soon afterwards a similar approach was applied during the NMR structure determination of a zinc finger protein, the C-terminal LIM domain from avian cysteine-rich protein (CRP) [165], and it was concluded that both proteins formed similar knuckle-like structures, in each case stabilised by N–H…S^γ^ hydrogen bonds spanning 2 or more residues. The sizes of these couplings were not reported, but the authors suggested the fact that these correlations were large enough to be detectable at all may indicate that such hydrogen bonds may have significant covalent character.

In 1988 Wörgötter *et al.* published a set of heteronuclear filters for one-dimensional experiments and half-filters for individual dimensions of two-dimensional experiments (or indeed multi-dimensional experiments) [249]. Since the widespread introduction during the 1990^0^s of global ^15^N and ^13^C labelling for NMR studies of proteins and nucleic acids, the use of such filters and halffilters in pulse sequences has become a very familiar and widely used part of the NMR “toolkit” (see for instance [250]), but at the time they were first published metallothionein represented a key test system, and was used to demonstrate the concepts and possibilities. Using this approach, it was shown that signals that were not coupled to ^113^Cd in one or more frequency dimensions could be selectively suppressed, leading to very considerable simplification of the spectrum and of interpretation; also, by varying the phase cycles within the filter elements such that they passed only ^1^H-^113^Cd-^113^Cd triple-quantum coherences, it was possible to identify specifically signals from bridging cysteines, provided the cysteine signals possessed non-zero couplings to both attached cadmiums.

Of course, the operation of all of the methods discussed in this section depends on the evolution of ^113^Cd heteronuclear J- couplings into an at least partially anti-phase state during delays in the pulse sequence, and there will always be relaxation losses associated with such delays. Consequently, the larger and more slowly tumbling the molecule, and/or the smaller the heteronu-clear coupling being exploited (and/or the broader the particular ^113^Cd signal involved), the lower will be the sensitivity of the experiment. Nonetheless, when they work, the correlation experi-ments described in this section provide some of the strongest and most direct evidence available by NMR to specify the structures of metallothioneins and zinc finger proteins.

##### 5.2.2.3 Using passive effects of ^113^Cd couplings

One potential issue with ^113^Cd correlation experiments is that the hardware they require may not always be conveniently available. All such methods of course require that the NMR spectrometer used must have an RF channel and probe capable of delivering ^113^Cd pulses, in addition to the ^1^H channel. While on modern spectrometers it is trivial to set the receiver frequency to ^1^H while pulsing on both ^1^H and ^113^Cd, at the time that some of these techniques were being developed in the early 1980^0^s there was a design assumption commonly built into spectrometers that pulsing on an X nucleus would always be associated with receiving at the frequency of X, with the ^1^H channel used exclusively for decoupling. For the experiments involving ^1^H detection in conjunction with both ^1^H and ^113^Cd pulsing described in the previous section, it was therefore necessary to modify the hardware to supply the receiver reference frequency from the ^1^H channel rather than the X nucleus channel; this arrangement was called “inverse detection” because the roles of “decoupler” and “transmitter” channels in such experiments were reversed relative to what was then considered normal operation. Even today, spectrometer hardware can present a significant limitation; at the time of writing, broad-band cryogenically-cooled probes capable of tuning to ^113^Cd are only available at specific field strengths, and hardware capable of ^113^Cd-^13^C–^1^H or ^113^Cd-^15^N-^1^H triple resonance experiments, particularly with cryogenically-cooled probes, is either rare or non-existent.

**Figure 22:**
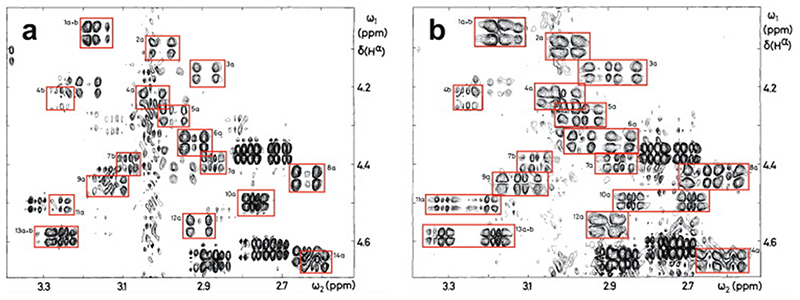
Passive effects of^113^Cd-^1^H couplings seen in phase-sensitive ^1^H–^1^H DQF-COSY spectra ofrabbit metallothionein MT2. The regions shown here contain the majority of the H^a^-H^b^cross-peaks from the 20 cysteine residues (as well as some Asp and Asn residues). Spectrum **a**) was recorded from ^112^Cd7 protein, spectrum **b**) was recorded from ^113^Cd7 protein, consequently spectrum **b**) shows additional splittings or broadenings due to ^113^Cd-^1^H J-couplings that are absent from spectrum **a**). The cysteinyl H^α^-H^β^ cross-peaks are marked with red boxes and numbered arbitrarily (sequential assignments were not yet available at the time the paper containing this figure was published), and positive and negative contour levels are plotted here without distinction. Conditions: 8–12 mM protein in ^2^H_2_O, 20 mM Tris buffer, 20 mM KCl, p^2^H 7.0, recorded at 500 MHz for ^1^H and 24 °C. Adapted with permission from[251] (© 1984 FEBS).

To sidestep these issues and obtain at least some useful information, one approach is to look for passive effects of ^113^Cd couplings, since these are present in spectra regardless of whether a ^113^Cd channel is available. As an alternative to decoupling in such cases, one can compare samples prepared with or without ^113^Cd, in order to establish which signals have additional splittings in the sample containing ^113^Cd. The best way to achieve a “decoupled” sample is to use isotopically pure ^112^Cd for the metal exchange, since if natural abundance cadmium is employed there will be substantial (approx. 10 %) sidebands from the ^113^Cd and ^111^Cd isotopes present (see Table 2), and if the native zinc-bound protein is used then chemical shift differences may complicate the interpretation. Fig. 22 shows an example of such a comparison for cysteine H^α^-H^β^ cross-peaks in phase-sensitive DQF-COSY spectra of rabbit metal-lothionein MT2. Clearly, a full analysis of such a comparison is complicated and would require detailed simulations, particularly to account for second-order effects within the spin systems. However, if the only information required is the identification of those spin-systems that show coupling to ^113^Cd this may be simpler to obtain, although even then spectral crowding and/or limited resolution could potentially defeat the analysis in a few cases.

In the case of ^113^Cd-^13^C or ^113^Cd-^15^N couplings, particularly those to aromatic resonances of histidines, spectral crowding is likely to be less of an issue, depending on what experiments are employed. As an example (the only one this author is aware of), Fig. 13b shows how ^113^Cd-^15^N couplings were detected as splittings in signals in long-range ^15^N-^1^H HMQC spectra of the ZZ domain of murine CREB-binding protein, and were used to help establish which of the histidine ring nitrogens are bound to cadmium in this case (see Section 5.1.2).

## 6 Conclusions; the future

In this review I have attempted to bring together the various factors that are particular to the structure determination of zinc finger proteins by NMR. It is fairly clear that over the past four decades NMR has played a more prominent role in structural studies of zinc finger proteins and domains than it has for many other types of proteins, for a combination of reasons discussed in earlier sections. However, times change, and to some degree structure determination ofrelatively small domains, by whatever technique, has become more of a routine activity than it was in the past. In words often attributed to Nils Bohr “prediction is difficult, particularly about the future”, but it seems relevant to ask: what does the future hold for such studies?

At the time of writing (early 2022), the field of structural biology is currently in the midst of two genuine revolutions: the advent of atomic resolution electron microscopy for biological macromolecules, which started a few years ago (see for instance [252]); and, more recently, the arrival ofaccurate computer predictions of protein structures based on sequence [164]. There can be no doubt that both of these developments are already having a profound and hugely positive impact on the whole world of biological research, and will continue to do so for the foreseeable future.

**Figure 23:**
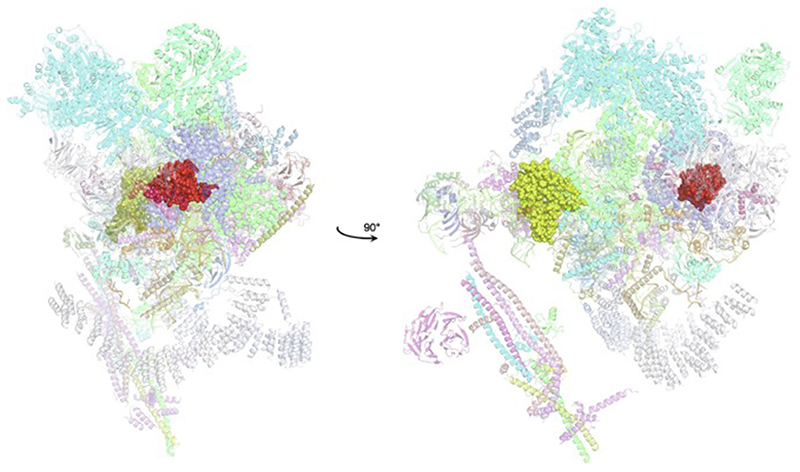
Rds3 and Bud31 in an EM structure of the yeast spliceosome. This structure from the Shi group was one of the first EM structures of an assembled spliceosome complex to be published[258], and corresponds to the so-called B^act^ complex on the splicing pathway, atwhich point the two ends ofthe exon that will be excised from the mRNA have been recognised and the catalytic components assembled and activated, but the splicing reaction itself has not yet started; see reference[258] for further discussion. The structure was determined at 3.5 Å resolution, and contains 38 proteins and 4 RNA chains with a combined molecular mass of ~ 1.6 MDa. In these views, created from PDB 5gm6 using the program PyMol[23,24], Rds3 is shown as red spheres, Bud31 as yellow spheres, and all other chains are shown translucent and colour- coded by chain ID. The intention in showing this figure is only to convey to non-specialist readers the relative size and complexity of the intact spliceosome assembly as compared to the Rds3 and Bud31 components; technical aspects of the structure and its determination, such as figures showing fitting of the final models of the intact assembly and of individual components into the experimental EM density, can be found in the original paper and associated supplementary information file[258].

The ability of EM to reveal detailed structures of highly complicated, complete macromolecular machines such as ribosomes, spliceosomes, proteasomes, anaphase-promoting complex, and many others without the need for crystallisation makes the mechanistic understanding of how such machines function far more accessible than ever before (see for instance [253] and references therein). These systems are typically built from large numbers of individual proteins, so it is possible for many new structures to be revealed in a single study, and the need to determine separate structures of the individual components, as would often have happened in the past, may sometimes be by-passed. In the cases ofthe two example proteins used to illustrate many ofthe points madein this review, Rds3 and Bud31, both are components of the yeast spliceosome, and a number of recent EM structures of spliceo-somes have included one or both of these proteins amongst the assembly components for which co-ordinates were deposited [254–284]; Rds3 also formed part of a spliceosomal sub-complex SF3b that was determined by X-ray crystallography [285]. However, this is not to say that the NMR structure determinations served no purpose. All of these structures were deposited subse-quent to the NMR structures, and close examination of the publica-tions reveals that almost all of the EM structures containing Bud31, and some of those containing Rds3, used the NMR co-ordinates during modelling of the electron density, either directly or indirectly (i.e. using co-ordinates of an earlier structure that itself used, or can be traced back to, the NMR co-ordinates for modelling; for modelling Rds3, later EM structures mainly used the SF3b X-ray structure). It is hard to know for certain how much impact this had, or what quality of structure would have been obtained for these components within the assembly had prior co-ordinates been unavailable, but in the end this probably comes down to a question of resolution; for as long as the resolution accessible for the structure of an isolated component exceeds that accessible for the same component within the intact assembly (which may in turn depend in part on local flexibility in the assembly), the iso-lated structure will continue to be useful. Fig. 23 shows one of the early EM structures of the yeast spliceosome (PDB 5gm6), which contains both Rds3 and Bud31 modelled using the NMR coordinates [286].

The likely impact of the availability of accurate structural predictions is more difficult to know. While the benefits of such predictions for the whole of biological research are undoubtedly immense, many, including this author, are intensely curious to know how it will affect the practice of experimental structure determination as a research activity in the longer term. It is of course vital to remember that protein functions cannot in general be fully understood from a single, static set of coordinates, whether from prediction or experiment. NMR is well-suited to studying dynamic behaviour, equilibria involving minor conformations and intrinsically disordered regions, whereas the input training data used during the development of alphafold2 was largely comprised of rigid, stable globular structures in the PDB database, mainly from crystallography. Consequently, as matters currently stand, alphafold2 cannot cast much light on these important issues of flexibility and dynamics. Nonetheless, even if putting that very important caveat to one side, it is clearly of great interest to see how experimentally determined average solution structures compare with alphafold2 predictions. For both Rds3 and Bud31, structures output by the alphafold2 program are available, and in both cases these are quite similar to the experimental co-ordinates,^2^ except that the predicted structures lack the zinc ions. However, both proteins were almost certainly part of the training set used during development of the alphafold2 program, if not directly in the form of the NMR co-ordinates then still as part of the various EM structures or the X-ray structure of the SF3b sub-complex, and it is hard for those without intimate knowledge of the program to know what significance this may or may not have had for the predictions in these specific cases. For zinc finger proteins more widely, one may reasonably expect that the great majority of those for which no experimental structure is currently available are likely to be homologous to sequences whose structures are known, and in these cir-cumstances alphafold2 is presumably highly likely to yield accurate predictions for the individual domains (though note the caveat discussed at the end of Section 4.5 concerning apparent predictions of relationships *between* finger domains, which are unlikely to be real in the majority of cases). However, in this author’s personal view it would be rather arrogant to assume that we now know everything, and one should not rule out the possibility that there could still exist novel zinc finger (or indeed metallothionein) structures, by which I mean structures only distantly related to those currently known, waiting to be discovered. It seems plausible that alphafold2 might perhaps have more difficulty with such cases than with other novel structures; zinc finger structures are largely defined by their metal-binding topology, and if starting only with sequence information this would have to be deduced entirely from scratch in the case of a truly novel structure. Indeed, alphafold2 would not even “know” a *priori* that such a protein binds zinc. In the original paper on alphafold2, the authors used a zinc site to exemplify the success of the program in predicting binding sites (see Fig. 1c of reference [164]), but it is worth noting that the site shown in the example chosen (PDB 6yj1, M23 peptidase domain of *Staphylococcal* phage 2638A endolysin) is an enzymatic zinc site rather than a structural one, which might perhaps make it somewhat easier to predict in the case of a truly novel structure. In the end, such issues in general probably come down to questions of the degree of homology. As the prediction programs continue to evolve, their ability to detect and use more distant homology relationships to known structures will likely continue to increase, and thus their predictive power will likely improve still further.

That said, it would be unfortunate indeed if the ability to test the truth of structural predictions were in future gradually to become lost through disuse. If an assumption that “the prediction is always right” were to become widespread and lead to a decline in the use of experimental structure determination, we would run the risk of failing to detect cases, however rare, where the prediction is wrong. This review is offered in the hope that it will prove useful to those who continue to seek experimental proof for structures.

## Figures and Tables

**Table 1:** Distance matrix for Cys Cb atoms in structures of the protein Rds3 calculated without (top right) or with (bottom left) all zinc-binding and inter-ligand distance restraints. ^a^.

Cβ-CβDistance	23	26	58	61	30	33	73	76	11	46	49	86
**23**		**5.18**	**4.54**	**6.61**	13.58	17.28	17.95	20.03	18.20	19.10	15.68	16.89
		**0.42**	**0.44**	**2.30**	0.56	0.80	0.68	0.39	0.67	0.70	0.68	0.40
**26**	**5.15**		**6.27**	**6.45**	9.01	12.46	13.44	15.83	17.33	18.86	16.49	15.82
	**0.13**		**0.54**	**1.24**	0.78	0.66	0.83	0.68	0.86	0.72	0.55	0.63
**58**	**3.74**	**5.17**		**5.73**	12.23	15.92	16.36	17.67	14.00	14.60	11.27	12.50
	**0.10**	**0.19**		**1.53**	0.62	0.52	0.61	0.34	0.56	0.50	0.67	0.48
**61**	**6.17**	**5.55**	**4.79**		12.49	14.13	16.64	17.85	13.80	16.61	13.66	13.94
	**0.16**	**0.30**	**0.06**		0.52	1.56	0.70	0.88	1.37	0.89	0.60	0.96
**30**	14.53	9.85	13.09	13.48		**5.87**	**4.47**	**7.20**	16.40	17.63	17.61	13.85
	0.20	0.24	0.20	0.22		**0.77**	**0.44**	**0.36**	0.62	0.50	0.54	0.37
33	18.11	13.13	16.45	15.59	4.95		**5.95**	**7.15**	16.79	19.57	20.10	15.56
	0.34	0.39	0.31	0.41	**0.19**		**1.56**	0.95	0.95	0.73	0.88	0.52
**73**	17.95	13.54	16.76	17.65	**4.34**	**6.65**		**3.95**	18.09	19.00	20.00	15.29
	0.25	0.30	0.24	0.35	**0.30**	**0.29**		**0.52**	0.73	0.70	0.69	0.52
**76**	20.03	15.62	17.97	18.47	**6.15**	**5.74**	**5.09**		16.29	17.15	19.02	13.58
	0.18	0.18	0.18	0.25	**0.20**	**0.32**	**0.11**		0.79	0.64	0.75	0.55
**11**	17.49	16.17	13.76	14.14	15.95	16.76	18.94	16.26		**6.30**	**7.35**	**4.94**
	0.42	0.57	0.40	0.48	0.45	0.42	0.49	0.64		**0.60**	**0.99**	**0.42**
**46**	19.35	18.64	15.76	17.66	17.78	19.49	19.90	17.38	**5.76**		**4.86**	**4.15**
	0.35	0.43	0.33	0.34	0.28	0.25	0.29	0.32	**0.22**		**0.83**	**0.36**
**49**	15.73	16.04	12.20	14.20	17.93	20.14	20.80	19.20	**5.92**	**5.10**		**6.22**
	0.36	0.52	0.39	0.43	0.22	0.17	0.28	0.32	**0.31**	**0.15**		**0.91**
**86**	17.23	15.83	13.57	14.93	14.64	16.08	17.12	14.57	**3.49**	**3.67**	**5.56**	
	0.51	0.56	0.48	0.50	0.16	0.15	0.27	0.34	**0.19**	**0.18**	**0.30**	

a For structures calculated without zinc-binding or inter-ligand restraints, no prior NOE cross-peak assignment information at all was included in the input data. Distances are grouped to show the binding sites that were identified (shown in bold). In each case the results show the mean Cys Cb-Cb distances (Roman) and standard deviations (italic) for the 10 lowest energy structures in an ensemble of 20 (no zinc) or 100 (with zinc) (these data values differ slightly from those published previously in [48] as they have here been re-calculated so as to correspond exactly with the same ensembles shown in Fig. 13). See text for further discussion.

**Table 2:** Atomic and nuclear properties of the stable isotopes of zinc and cadmium. ^a^.

Nucleus	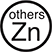	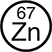	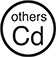	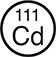	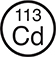
Nuclear spin (*I*)	0	5/2	0	1/2	1/2
Natural Abundance ^b^	95.90 %^c^	4.10%	74.98 %^d^	12.80%	12.22 %
NMR frequency (Ξ) (MHz) [for ^1^H at 500 MHz] ^e^	–	31.3395	–	106.077390	110.965865
Gyromagnetic ratio (γ) (10^7^ rad/T s^-1^)^e^	–	1.6768	–	- 5.6926	- 5.9550
Receptivity vs ^1^H [prop. to γ^3^I(I + 1)] ^e^	–	0.287 %	–	0.965 %	1.10%
Receptivityvs ^13^C [prop. to γ^3^I(I+ 1)] ^e^	–	18.06 %	–	60.57 %	69.32 %
Nuclear quadrupole moment Ԛ (barns; 1b = 10^-28^ m^2^)	0	+ 0.15^f^	0	0	0
Atomic radius (Å) ^g^	1.35	1.35	1.55	1.55	1.55
Metal – sulphur bond length in MT (Å) ^h^	2.34 ± 0.03	2.34 ± 0.03	2.54 ± 0.02	2.54 ± 0.02	2.54 ± 0.02

a Using a very strict definition, “^3^Cd is not a stable isotope as it undergoes extremely slow βdecay (half-life 8.04 ×10^15^ years)[186].

b Isotopic abundance data taken from Rosman[187].

c The figure of 95.90% is a sum over the natural abundances of all the various other stable isotopes of zinc, all of which have nuclear spin quantum number of 0. Individually they comprise: ^64^Zn, 48.63%; ^66^Zn, 27.90%; ^68^Zn, 18.75%; ^70^Zn, 0.62%.

d The figure of 74.98 % is a sum over the natural abundances of all the various other stable isotopes of cadmium, all of which have nuclear spin quantum number of 0. Individually they comprise: ^106^Cd, 1.25 %; ^108^Cd, 0.89 %; ^110^Cd, 12.49 %; ^112^Cd, 24.13 %; ^114^Cd, 28.73 %; ^116^Cd, 7.49 %.

e Data taken from Harris [188], appendices 1 and 2; receptivity data as shown here is re-calculated assuming 100 % isotopic abundance in all cases, and NMR frequencies are re-calculated for Ξ ^1^H = 500.000000.

f Value taken from Stone [189].

g The atomic radii are taken from [190], and were reported for natural abundance material; they are here assumed to be the same for each isotope.

h Bond lengths given here are those reported byGui etal. [191], and were measured by EXAFS. They were reported for natural abundance material and are here assumed to be the same for each isotope of the same element within the error limits given in the original publication.

**Table 3:** Examples of methods used to prepare cadmium-substituted zinc finger proteins for NMR studies.

Protein	Type ^a^	Exchange reaction / conditions ^b^	Reference
Rabbit metallothionein (MT1 and MT2) ^c^	2 clusters (C_9_M_3_ and C_11_M_4_)	Zn,CdMT2 from rabbit liver homogenate incubated with 70 μM CdCl_2_ for 15 min at room temp., then purified by gel-filtration and ion exchange. Some preparations using this method had inhomogeneous metal-binding topology (see Fig. 16a).	Otvos and Armitage[198] (see also Suzuki *et al.* [199])
Rabbit metallothionein (MT2)	2 clusters (C9M3 and C_11_M_4_)	Stepwise addition of 0.5 M CdCl2 into purified Zn7-MT2 (0.5–2 mM) up to stoichiometric ratio (7:1), with removal of free Zn at each step using Chelex 100 resin.	Otvos *et al.* [200]
Rabbit metallothionein (MT2)	2 clusters (C_9_M_3_ and C_11_M_4_)	Apo-MT2 (0.5 mg.L^-1^) in 0.1 M HCl mixed with 7–7.5 equiv. of CdCl_2_ solution, then adjusted to pH 6.4 with 0.5 M MES or pH 8.6 with Trizma. Mixture and all solutions degassed, excess Cd removed by dialysis.	Vašák *et al.* [201] (see also [197,202–204])
GAL4 (1–65)	C_6_M_2_ cluster	Extensive dialysis of Zn_2_-GAL4 against excess CdCl_2_	Baleja *et al.* [36]
GAL4 (7–49)	C_6_M_2_ cluster	Adding a 5-fold excess of Cd(II) to the Zn(II) protein.	Gadhavi *et al.* [205]
GAL4 (1–62)	C_6_M_2_ cluster	Adding Cd(II) to apoprotein at pH 8.0 in presence of excess BME. or Incubate Zn2- GAL4 with 3-fold molar excess of Cd(II) and excess BME for 12 h at 22 °C (most rapid at pH 5.0). Dialysis against metal-free buffer removes Zn(II) and Cd(II), then repeat whole process to yield Cd2-GAL4.	Gardner *et al.* [206] and Pan and Coleman[73]
LAC9 DNA-binding domain (LAC9 85–144 or 85–288)	C_6_M_2_ cluster	Incubate 2-fold molar excess of Cd(II) with Zn2-LAC9 and excess BME for 15 h at room temp. followed by removal of free Zn(II) by a size-exclusion column, then repeating the entire procedure (first pass yields Cd_0.9_Zn_1.1_-LAC9).	Gardner *et al.* [207] and Pan et al.[208]
Glucocorticoid receptor DNA-binding domain (GR 440–525)	C_4_, C_4_	Incubate 3-fold excess Cd(II) with Zn_2_-GR for at least 12 h at room temperature, followed by dialysis to remove free Cd(II) and Zn(II).	Pan *et al.* [209]
Sp1-resembling synthetic peptide	C_2_H_2_	Addition of Cd^2+^ to apo-protein ^d^	Razmiafshari *et al.* [210]
TFIIIA finger 3 (mutated to increase metal affinity – see paper for details)	C_2_H_2_	Addition of Cd^2+^ to apo-protein ^d^	Krepkiy *et al.* [211]
HIV nucleocapsid protein	CCHC	Cells [of E. coli containing overexpressed zinc-bound protein] sonicated for 3 min in 50 mM potassium acetate, 100mM KCl,5 mMBME,2 mM CdC2, 0.03 mg/mL PMSF at pH 5.0.	Fitzgerald and Coleman [212]
CBP ZZ domain (CBP 1700–1751)	C_4_, C_2_H_2_	Refolding urea-denatured protein into buffer containing 10 lM ^113^CdCl_2_.	Legge *et al.* [176]
CNOT4(1–63)andp44(321–395)	C_4_,C_4_	Addition of 40 mM Cd-EDTA into Zn2-CNOT4 (>6-fold excess of Cd^2+^ over protein) or Zn2-p44 (3-fold excess of Cd^2+^ over protein) at 27 °C, monitoring exchange by repeatedly acquiring ^15^N-HSQC spectra.	Houben *et al.* [72] (see also Hanzawa et al.[213])
MSL2 CXC domain (MSL2 517–572)	C_9_M_3_ cluster	“For ^113^Cd labelling, ZnCl_2_ in M9 media was substituted with 10 nM ^113^Cd acetate. E. coli growth and protein yields were normal in ^113^Cd-containing media. ”^e^	Zheng *et al.* [59]
Bud31	C_9_M_3_ cluster	10-fold dilution of apo-protein into 25-fold excess of CdCl2 and subsequent incubation for 1 day at 0 °C, then excess CdCl2 removed by buffer exchange. Mass spectrometry used to check every preparation (if incomplete substitution was detected, the whole process repeated)	van Roon *et al.* [49]

a M here refers to metal, either Zn or Cd.

b Details given here summarise descriptions, which are sometimes incomplete, in the references cited.

c Metallothionein MT1 and MT2 are isoforms of metallothionein with slightly different amino acid sequences.

d This is a single C_2_H_2_ finger peptide with relatively low metal-binding affinity, so binding is straightforward.

e Culturing cells in growth medium containing ^113^Cd(II), as in this example, leads to relatively large volumes ofcadmium-contaminated waste, which would require careful risk assessment before disposal.

## Glossary

ADD: ATRX-DNMT3-DNMT3L
AMBER: Assisted model building with energy refinement
APLF: Aprataxin and PNK–like factor
ARIA: Ambiguous restraints for iterative assignment
ATP: Adenosine triphosphate
ATRX: Alpha thalassemia/mental retardation syndrome X-linked
BME: b-mercaptoethanol
BMRB: BioMagResBank (https://bmrb.io/)
BUD: Genes associated with budding in Saccharomyces cerevisiae
C2H2: Zinc finger that co-ordinates zinc through two Cys residues and two His residues
C3H: Zinc finger that co-ordinates zinc through three Cys residues and one His residue (note the position of the His is not intended to be specified by this notation, and can vary)
C4: Zinc finger that co-ordinates zinc through four Cys residues
CBP: Cyclic-AMP response element binding protein
CD: Circular dichrosim
CNOT: Ccr4-Not (carbon catabolite repression 4 – negative on TATA-less) tran scription complex subunit 4
CNS: Crystallography and NMR system
COSY: Correlation spectroscopy
CP-1: Consensus peptide 1 CREB cAMP (cyclic adenosine monophosphate) response element-binding (protein)
CRISPER: Clustered regularly interspaced short palindromic repeats
CRP: Cysteine rich protein
CSA: Chemical shift anisotropy
DBD: DNA binding domain
DEAF-1: Deformed epidermal autoregulatory factor 1
DNA: Deoxyribonucleic acid
DNMT3: DNA (cytosine-5)-methyltransferase 3
DNMT3L: DNA (cytosine-5)-methyltransferase 3-like
DQF: Double-quantum filter
DTT: 1,4-Dithiothreitol
DYANA: Dynamics algorithm for NMR applications
EAA: Early endosome antigen
EM: Electron microscopy
EDTA: ethylenediaminetetraacetic acid
EDXRF: Energy dispersive X-ray fluorescence spectroscopy
EHV: Equine herpes virus
EMSA: Electrophoretic mobility shift assay
ERDBD: Oestrogen (U.S. spelling, Estrogen) receptor DBD
ESI: Electrospray ionisation
EXAFS: Extended X-ray absorption fine-structure
FYVE: Protein domain named after four proteins in which it is found Fab 1, YOTB, Vac1, and EEA1
GAL4: One of a number of regulatory genes required for the growth of yeast on galactose
GATA: GATA (i.e. Gua-Ade-Thy-Ade sequence of DNA)
GRDBD: Glucocorticoid DBD
HEPES: (4-(2-Hydroxyethyl)-1-piperazineethanesulphonic acid)
HIV: Human immunodeficiency virus
HMQC: Heteronuclear multiple quantum correlation
HSAB: Hard-soft acid-base
HSED: Heteronuclear spin-echo difference
HSQC: Heteronuclear single quantum correlation
ICP-OES: Inductively coupled plasma – optical emission spectroscopy
LAC9: Lactose regulatory protein 9
LIM: Protein domain named after three proteins in which it is foundLin-11, Isl-1 and Mec-3
MBP-1: Major histocompatibility complex enhancer binding protein 1
MES: 2-(N-morpholino)ethanesulphonic acid
Miz-1: c-Myc-interacting zinc finger protein-1
MOPS: 3-(N-morpholino)propanesulphonic acid
MYND: Myeloid-Nervy-DEAF1 domain
mRNA: messenger RNA
MSL2: MSL (male-specific lethal) complex subunit 2
MT: Metallothionein
MTF-1: metal-responsive element binding transcription factor-1
NMR: Nuclear magnetic resonance
NOE: Nuclear Overhauser effect
NOESY: NOE spectroscopy
o.d.: outer diameter
PAR: Poly(ADP-ribose)
PARP: Poly(ADP-ribose) polymerase
PDB: Protein data bank
PHD: Plant homeodomain
PIPES: piperazine-N,N^0^-bis(2-ethanesulphonic acid)
PIXE: Micro-beam proton-induced X-ray emission (also abbreviated micro-PIXE)
PMSF: Phenylmethylsuphonyl fluoride
PONDR: Predictor of natural disordered regions
RDC: Residual dipolar coupling
RDS3: Regulator of drug sensitivity 3
RF: Radiofrequency
RING: Really interesting new gene
RNA: Ribonucleic acid
RSGI: Riken Structural Genomics Initiative
Sp1: Specificity protein 1
SWI5: One of a cascade of “switch” proteins involved in transcription regulation leading to differentiation of mating type in yeast cells
TAZ: Transcription Adaptor Zinc finger
TCEP: (tris(2-carboxyethyl)phosphine)
TFIIIA: Transcription factor IIIA (so named as it was one of three factors, A, B and C, involved in the specific regulation of Xenopus 5S RNA genes that are transcribed by RNA polymerase III [287]).
TOCSY: Total correlation spectroscopy
Tris: Tris(hydroxymethyl)aminomethane
UV: Ultra-violet

## Footnotes

1 Of the remainder, 202 were excluded because they reported no bonding information in the molecular descriptions, while 31 were excluded because no zinc or cadmium that they contained was bound by at least two cysteines; these latter cases were mostly enzymes. The earliest depositions found dated from 2002, implying that many earlier studies are not included in this set, either because they were not deposited at BMRB (which was created in 1996) or because at that time the depositions did not include bonding information. Note also that any structures of zinc fingers that do not include at least one zinc or cadmium ion in the deposited coordinates are not included in this set; no effort was made to identify such cases systematically. Nonetheless, overall this selection of 501 cases constitutes a useful set to provide a substantial and reasonably complete overview for the purposes of this review.
